# On the Design of *Chlorella vulgaris* Composition for Potential Food Uses via Manipulation of Cultivation Conditions

**DOI:** 10.3390/md24040124

**Published:** 2026-03-26

**Authors:** Ana S. Pinto, Joana Oliveira, Ana F. Esteves, Susana Casal, Gustavo Mil-Homens, Francisco X. Malcata, José C. M. Pires, Tânia G. Tavares

**Affiliations:** 1 LEPABE (Laboratory for Process Engineering, Environment, Biotechnology and Energy), ALiCE (Associate Laboratory in Chemical Engineering), Faculty of Engineering, University of Porto, Rua Dr. Roberto Frias s/n, 4200-465 Porto, Portugal; up202008182@edu.fe.up.pt (A.S.P.); up201904660@edu.fe.up.pt (J.O.); afcesteves@fe.up.pt (A.F.E.); up202106070@edu.fe.up.pt (G.M.-H.); fmalcata@fe.up.pt (F.X.M.); jcpires@fe.up.pt (J.C.M.P.); 2 LAQV-REQUIMTE (Associated Laboratory for Green Chemistry-Network of Chemistry and Technology), Faculty of Pharmacy, University of Porto, Rua de Jorge de Viterbo Ferreira 228, 4050-313 Porto, Portugal; sucasal@ff.up.pt

**Keywords:** microalgae, health foods, N:P ratio, temperature, optimization, biomass productivity

## Abstract

Interest in microalgae-based technologies has emerged in recent years as a response to environmental challenges and the global food crisis, for providing alternative and sustainable food products. This study used temperature variations between 18 and 32 °C and nitrogen-to-phosphorus (N:P) ratios between 1.9 and 42.6 to model and optimize growth and composition of *Chlorella vulgaris*, a nutritionally interesting species. Lower temperatures appear ideal for this strain. An increase in average biomass productivity was observed with decreasing temperature, leading to a maximum of 122.27 mg_dw_ L^−1^ d^−1^ at 18 °C on the fourth day of cultivation. The maximum productivities for total proteins, fatty acids, carbohydrates, and pigments were, respectively, 26.9 mg L^−1^ d^−1^, 26.4 mg L^−1^ d^−1^, 16.0 mg L^−1^ d^−1^, and 2.41 mg L^−1^ d^−1^, all referring to 18 °C. The fatty acid, carotenoid, and amino acid profiles were also ascertained; several indicators suggested that cultivation of these microalgae under the aforementioned optimal conditions holds potential for the food industry. The high proportion of polyunsaturated fatty acids—including two essential fatty acids; the high production of lutein, and the presence of several essential amino acids are among the favorable indicators. Overall, the information generated by this study is helpful to support future pilot studies aimed at the commercial production of microalgae-derived products.

## 1. Introduction

Microalgae are unicellular photosynthetic organisms, present in terrestrial and aquatic ecosystems. Collectively, they release around 50% of the world’s oxygen (O_2_) available in the atmosphere [1]. Besides consuming gaseous carbon dioxide (CO_2_) and common water pollutants, such as nitrogen and phosphorus compounds, and thus being frequently used in bioremediation solutions, microalgae are valuable in several markets, such as food, feed, energy, and chemicals (including pharmaceutics and cosmetics) [2]. The attributes mentioned so far are common to terrestrial plants. However, microalgae exhibit (i) higher areal biomass productivity (estimated to be ca. tenfold), (ii) no competition for farmland, a growingly scarce resource, and (iii) the ability, for many species, to grow on seawater and wastewater [3,4,5]. Therefore, they effectively contribute to addressing several sustainable development goals (SDGs); more specifically, the autotrophic cultivation of microalgae as a food source targets SDG-2 (zero hunger), SDG-12 (responsible consumption and production), and SDG-13 (climate action) [6].

Nowadays, microalgal biotechnology constitutes a highly active and growing research field, driven by attempts to provide robust responses to the increasing consumer demand for microalgae-based products [7]. In particular, *Chlorella vulgaris* (*C. vulgaris* for short), a species of green microalgae [8] that can adapt to both freshwater and seawater [9,10,11], has recently received expanding attention [12], primarily due to its interesting chemical composition in (i) key nutrients, such as proteins (ca. 51–58% dry weight, reaching higher values than conventional human food sources [13,14,15]), carbohydrates (12–23%) and lipids (10–22%), as well as in (ii) bioactive compounds carrying multiple health benefits. These include pigments, polysaccharides, vitamins, and polyunsaturated fatty acids (PUFAs), which are all considered to be high-value products. Besides pharmaceutics, they can be applied within the food industry and have contributed to the growing portfolio of commercially available supplements and functional foods [12,13,16,17].

The specific biochemical composition of microalgal biomass, which directly determines its economic value, is influenced not only by the species but also highly by cultivation conditions, such as culture medium type and abundance of carbon sources, temperature, salinity, pH, light intensity, wavelength, and photoperiod [15,16]. This study focuses on two environmental variables: the initial nitrogen to phosphorus (N:P) ratio (relative molarities of sodium nitrate (NaNO_3_) and monopotassium phosphate (KH_2_PO_4_)) and the temperature (T). They were chosen chiefly for their proven influence upon biomass composition and relevance in industrial cultivation settings. Several authors have worked previously with either the N:P ratio [18,19,20,21,22,23,24,25,26], temperature [27,28,29,30,31,32], or both [33,34,35] to induce different metabolic responses in *Chlorella* species or similar green microalgae. Besides conventional optimization studies, a review by Esteves et al. [2] covered a new wave of attention (active from 2019 onwards) dedicated to the strategies of stress induction and two-stage cultivation. While results are diverse and often too complex for effective comparisons, there is some consensus around the response of biochemical profiles to variations in cultivation conditions, which can be used to define final product specifications rationally based on causal relationships.

Both nitrogen and phosphorus are growth-limiting macronutrients for microalgae. Nitrogen is essential for protein synthesis, since all amino acid residues structurally contain an amino group (NH_2_). Proteins and enzymes are at the base of an organism’s structure and metabolic regulation, being intrinsically associated with growth. Phosphorus is necessary for the synthesis of nucleic acids (DNA and RNA), ATP (adenosine triphosphate, the cell’s molecular energy mediator [36]), and the phospholipids in the cell membrane [1,22,37]; hence, it is essential for photosynthesis, signaling pathways, and vital metabolic processes. Feeding these nutrients to the culture at different concentrations and/or ratios is a proven way to manipulate metabolic responses [2,38]. High concentrations of N and P stimulate protein production [24,39,40], while starvations lead to lipid and carbohydrate accumulation [41]—with possible conversion of carbohydrates into lipids from N limitation to complete depletion [24]. Regarding the profile, lipids with energy storage functions (neutral, like those richer in saturated fatty acids (SFAs)) are also favored over those with structural functions (polar lipids, usually richer in monounsaturated fatty acids (MUFAs) and PUFAs) [38,42]. Interestingly, P supplementation in cultures under N starvation boosts lipid productivity but causes cell damage when in excess [43,44]. Finally, no nutrient limitation favors chlorophyll, a compound produced during the exponential growth stage [24,25,45]. However, carotenoid contents, especially β-carotene and lutein, have been reported to increase under N stress [46,47] and, in some instances, nutrient repletion, which can be explained by the increased synthesis of proteins of the light-harvesting complex that bind to carotenoids [24,48].

Understanding the response of microalgae to temperature oscillations is also of paramount importance, since the mass cultivation of these organisms relies mainly on open pond systems [49], where the controllability of this parameter is inherently poor. Temperature fluctuations below and above each species’ optimum values impact not only biomass productivity, but also gene expression, metabolism, and composition [2]. While there are often misalignments and discrepancies [2,30,42], it is possible to generally relate cold regimes to (i) slower growth [50], (ii) higher carbohydrate production for structural protection, and (iii) preference for unsaturated fatty acid (UFA) accumulation over SFAs meant for increased membrane fluidity [27,50,51]. Meanwhile, stress at higher temperatures leads to (i) a carbon flux shift toward lipogenesis [30,52,53], (ii) the opposite lipid profile tendencies with a larger SFA fraction [42,53], (iii) accumulation of antioxidant molecules, such as carotenoids, meant to oppose cell damage induced by reactive oxygen species (ROS) [30,54,55], and (iv) collapse of the photosynthetic apparatus and permanent enzyme damage if above the tolerance interval for each species [52,56,57].

**Figure 1:**
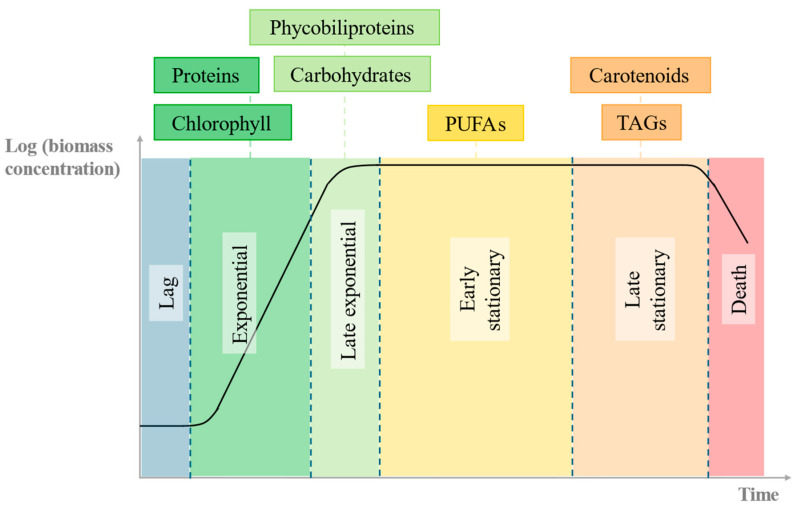
Schematic representation of the growth cycle of microorganisms, and some of the most relevant biomolecules produced by microalgae that tend to accumulate at each growth phase. Adapted from Gifuni et al. [59] and Esteves et al. [2,16].

Additionally, there are considerable changes in *C. vulgaris* physiology and morphology across the typical growth stages observed in a batch cultivation experiment (lag, exponential growth, stationary, and death stages [2,58]), reflecting metabolic adaptations to the gradual nutrient depletion in the growth medium [2,58,59]. Figure 1 illustrates some of the macromolecules that cells typically prioritize in their carbon and energy fluxes throughout the growth cycle.

While a significant amount of research relates specific cultivation parameters to the production of certain target compounds in the case of *C. vulgaris*, the information is still dispersed among many small-scale, often one-parameter studies. These are usually difficult to compare due to inevitable non-uniformities in specific conditions, and the realization that conclusions reached at bench scale are seldom translatable directly to pilot or industrial scale cultivation. Additionally, most authors analyze the biomass composition only once after the established cultivation period, and even when other time points are monitored, no previous studies (to the best of our knowledge) convey information on the effect that the growth stage during which harvesting occurs can be expected to have upon the observed composition.

This work aims to address the foregoing gaps of knowledge: it consists of an integrated optimization study on two variables, complemented by the time evolution of biochemical profiles, so as to pick up the optimal harvesting time in terms of the highest accumulation of the target compounds. Integrated databases such as this one, complemented with defined nutraceutical goals, can additionally support the provision of better-quality products by the industry of food supplementation and healthy foods, thus facilitating shifts in focus from only bulk-biomass productivity to enhanced nutritious profiles.

In this study, photoautotrophic *C. vulgaris* was cultivated in 12 batch experiments in closed bioreactors, varying temperature and N:P ratio (both isolated and combined) according to the points of a central composite design (CCD), a common design of experiments (DoE). The culture growth and nutrient uptake were monitored, and biomass fractions were harvested at strategic growth stages. The most relevant compounds—viz. total fatty acids (total FA), FA profile, total pigments, carotenoid profile, total carbohydrates (TC), total proteins (TP), and amino acid profile were then extracted and quantified using appropriate analytical procedures. Response surface methodologies (RSM) were finally applied to the experimental data, aiming to find predictable trends and optimum points.

## 2. Results and Discussion

### 2.1. Biomass Growth and Nutrient Consumption

Table 1 discriminates the conditions corresponding to each of the 12 experimental runs, numbered Experiment 1 to Experiment 12 (E1 to E12), according to their position in the rotary CCD. To obtain these values, the two continuous independent factors studied, the temperature (18–32 °C range) and the N:P ratio (in the form of its logarithm of base three, to cover the desired range), were coded into the variables *x*_1_ and *x*_2_, and assumed the levels 0, −1 or +1 and −$\sqrt{2}$, or +$\sqrt{2}$ (parameter α). As is common practice with experimental design studies, biological replication (in this case, four replicas) was performed on the central point only.

**Figure 2:**
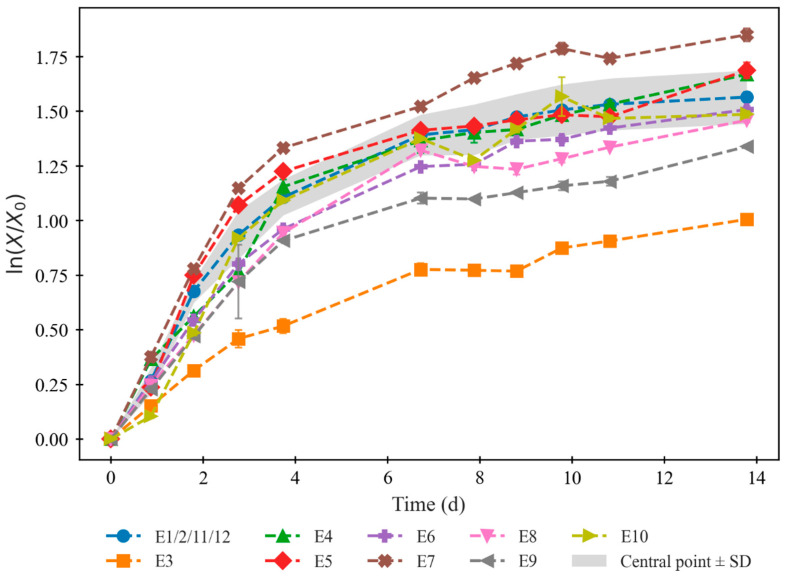
Growth curves for the 14-day cultivation of *C. vulgaris* under the conditions defined by experimental runs 1 through 12. The biological replicas of the central point (E1, E2, E11, and E12) are represented by their average, surrounded by a shaded area defined by their standard deviations, for better comparison of each curve’s behavior to that of the central point.

Figure 2 depicts the growth curves for the set of assays defined by E1 to E12. They exhibit the normalized natural logarithm of biomass concentration values along the cultivation (see Supplementary Materials for the raw data and curves of biomass concentration). In all of them, an early-exponential growth stage lasting for the first four days is visible, followed by a deceleration of growth that can be attributed to the late-exponential stage. Days 7 through 14 are consistently characterized by slower growth, but OD readings identified no death stages. These trends confirm and validate the initial assumption of days 4, 7, and 14 as strategic harvesting times (besides day 0 as the control/starting point), since they mark the limits of sequential phases.

**Figure 3:**
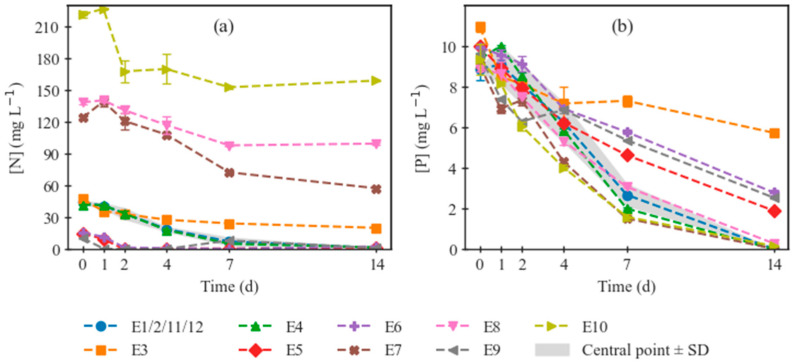
Nitrogen (NO_3_-N) concentrations (**a**) and phosphorus (PO_4_-P) concentrations (**b**) in the culture medium along 14-day cultivations of *C. vulgaris* under the conditions defined by experimental runs 1 through 12. The biological replicas of the central point (E1, E2, E11, and E12) are represented by their average, surrounded by a shaded area defined by their standard deviations, for better comparison of each curve’s behavior to that of the central point.

Figure 3 compiles the nutrient curves, i.e., the evolution of NO_3_-N (Figure 3a) and PO_4_-P (Figure 3b) in the medium, thus informing on the consumption of these compounds by the microalgae. While comparison between the curves on (Figure 3b) is essentially indicative of the culture metabolic performance—and can, in most cases, be related to the growth curves on Figure 2, it is noteworthy that the nitrogen curves on (Figure 3a) are more complex to evaluate qualitatively, since the initial concentrations are entirely disparate, to fulfil the molar ratios defined by the DoE in Table 1. It should be noted that P was not varied in this study to conserve some simplicity and thus control over the causes within the limited number of experimental points, since it would be challenging to know if a certain observed effect would be due to N abundance or P deficiency, or vice versa. Complementing the visual information, Table 2 compiles the quantitative parameters for biomass growth and nutrient consumption.

According to RSM, the quantitative parameters can be analyzed as response variables in regression models (Equation (1) Section 3). These tools and data treatment allow the detection of trends and possible relationships between variables that can go unnoticed when observing simple result listings. The first results were tentatively fitted by a model as *μ* values, with *x*_1_ and *x*_2_ as dimensionless independent variables. Even though the initial *R*^2^ of the regression, 0.75, was satisfactory to some degree, after backward elimination of the non-significant terms, leaving only *x*_1_ (linear temperature level), the correlation coefficient was below 50%, thus invalidating the model. In fact, since this parameter is computed with only the first four optical density (OD) measurements, representing the early exponential stage, it is possible that this small fraction of the cultivation period is not affected by the independent variables to the point that the response shows clear tendencies. There can also be factors unaccounted for that shape growth rate in a relevant way, such as the initial biomass concentration or composition.

**Figure 4:**
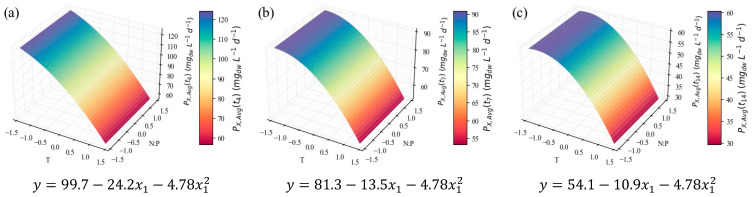
Surface response plots for the average biomass productivity ($P_{X,avg}$, mg_dw_ L^−1^ d^−1^) of *C. vulgaris* cultivations varying temperature and N:P ratios at the end of *t*_4_ (**a**), *t*_7_ (**b**), *t*_14_ (**c**), and respective regression equations. All independent variables and regression coefficients are presented in coded units.

The average biomass productivity results ($P_{X,avg}$) were more promising. Since these were calculated at three points in time (*t*_4_, *t*_7_, and *t*_14_), time was inserted into the models as a categorical independent variable. This meant that its linear isolated term and its linear interactions with x_1_ and x_2_ were considered in the regression; however, they were not considered the same way as the continuous variables that defined the CCD; rather, they were considered at each discrete time point. This allows for inclusion of this parameter in the response optimization, prone to inform on the optimal harvesting time to maximize the desired response. This strategy was applied to all further results that depend on time. The $P_{X,avg}$ models are presented as three separate surface plots and their corresponding equations (Figure 4), representing the average productivity at the end of the early-exponential (Figure 4a), late-exponential (Figure 4b), and stationary (Figure 4c) growth stages. These results unfold a strong relationship between temperature and biomass productivity, described by a downward-oriented parabola within the tested domain. As the productivity is averaged along a larger section of the microalgal growth cycle (*t*_4_ to *t*_14_), the parabola is shifted downwards and along the positive direction of the *x*_1_ axis, along with the position of its vertex and symmetry axis. Table 3 presents the leading indicators of model performance and its overall optimal point. It can be considered an excellent fit for explaining 93% of the variation (*R*^2^ = 0.93). The close values of adjusted and predicted *R*^2^ indicate good predictive ability and limited overfitting. $P_{X,avg}$ can be maximized at the end of the early-exponential phase (*t*_4_) and at the minimum temperature level ($x_{1}=-\sqrt{2}$, that is, a temperature value of 18 °C), which is consistent with the negative $\beta_{1}$ and $\beta_{11}$ coefficients.

The role of temperature, as evidenced by the model, is consistent with the data in Table 2, and the *μ*-values even follow the trends therein. For instance, the growth and cell productivity during the exponential stage are clearly stronger in E4, E5, and E7 assays, in which lower temperatures were applied, and weaker in E3, E6, and E8, all characterized by higher temperatures. *C. vulgaris*’ preference for temperatures between 18 and 25 °C was previously reported by Serra-Maia et al. [60], who modeled growth and mortality rates and predicted an optimal *μ* at 23.3 °C. The enhanced enzymatic kinetics explained the slight increase the authors observed from 18 °C to the optimum value, in accelerating photosynthesis and cell division. However, from 25 to 30 °C and above, there was a sharp decrease in growth rate, explained by oxidative damage and protein degradation. There is also evidence that temperatures above 30 °C jeopardize CO_2_ solubility in the growth medium; therefore, its availability for consumption is compromised [61,62]. It is important to note, however, that this behavior is strain-dependent; other authors report optimal temperatures for *C. vulgaris* in the range of 25–30 °C [63,64], with the possibility of survival at higher temperatures, particularly following more extended acclimation periods [65]. However, in industrial settings, fast temperature fluctuations, with no time for acclimation, are expected in photobioreactors or open ponds exposed to outside conditions [60]. This suggests that the strain used in the present work should only be used for production in temperature-controlled conditions, or regions with a colder climate.

On the other hand, the N:P ratio variable was considered non-significant for the $P_{X,avg}$ models (Figure 4, Table 3, Table 4 and Table 5); and its contribution to the values in Table 2 is not apparent, except for the E9 experiment, in which the lowest N concentration coincided with the lowest growth rate. In one of the most thorough studies on the impact of different N:P molar ratios (from 2 to 67), using the same *C. vulgaris* strain and similar conditions, including growth medium, it was concluded that the initial nitrogen and phosphorus concentrations do not notably impact biomass growth during the exponential growth phase. The authors suggest shifts in the microalgal flux rates, including nutrient consumption rates, in response to the prevailing environmental conditions, in attempts to keep the growth patterns [22]. The authors admitted, however, the possibility of lower N concentrations hindering growth, which can explain the E9 observation in the present study (with an initial nitrogen concentration of 10.3 ± 0.1 mg L^−1^, close to the lower limit of the interval tested by Salgado et al. [22], 9.7 ± 0.2 mg L^−1^). Other literature sources [66,67] corroborate the low impact of the N:P ratio upon microalgal growth rate, within reasonable limits.

In view of the above, nutrient removal parameters can also be analyzed in the light of Salgado et al.’s work [22]. The *RE* values close to 100% indicate that an N:P ratio of 9, with a P concentration anchored around 10 mg L^−1^ (the typical composition of the OECD growth medium), is adequate for the uptake capacity of *C. vulgaris*. Higher N concentrations lower the NO_3_-N removal efficiency but increase the NO_3_-N *RR*. Conversely, lower N concentrations (ratios of 3 and below) decrease the NO_3_-N *RR*, thus negatively impacting the P uptake. These observations all agree with the aforementioned study [22]. Phosphorus limitation would probably also limit nitrogen uptake, but the present work did not include such a variable.

### 2.2. Biochemical Composition—Total Analyses

**Figure 5:**
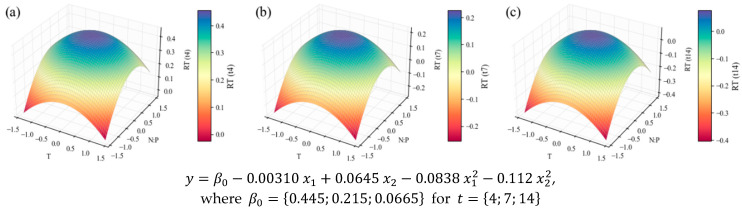
Surface response plots for the relative tendency of the TP content (% *w*/*w*) of *C. vulgaris* cultivations varying temperature and N:P ratios at the end of *t*_4_ (**a**), *t*_7_ (**b**), and *t*_14_ (**c**), with respective regression equations below. All independent variables and regression coefficients are presented in coded units.

The biomass harvested on days 0, 4, 7, and 14 of the 12 experiments was subjected to analyses to assess its general composition. The average content values are presented in the Supplementary Material. The model equations and surface plots for the *RT* (relative tendency; see Section 3) of the content in TP, at the three points of interest, are visible in Figure 5.

**Figure 6:**
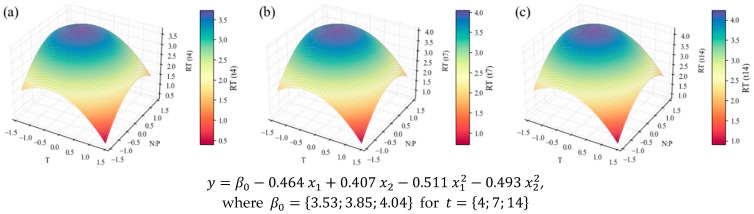
Surface response plots for the relative tendency of the TP concentration (mg L^−1^) of *C. vulgaris* cultivations varying temperature and N:P ratios at the end of *t*_4_ (**a**), *t*_7_ (**b**), and *t*_14_ (**c**), respective equations below. All independent variables and regression coefficients are presented in coded units.

Once in possession of the volumetric biomass concentration in the bioreactor at each point in time (Table S1 and Figure S7, Supplementary Material), it is interesting to multiply it by the percentual contents to obtain the maximum concentration (in mg L^−1^) of each nutrient in the medium. The models and surface plots were produced after normalizing each result by the *t*_0_ values. These results, as applied to the TP data, are presented in Figure 6.

**Figure 7:**
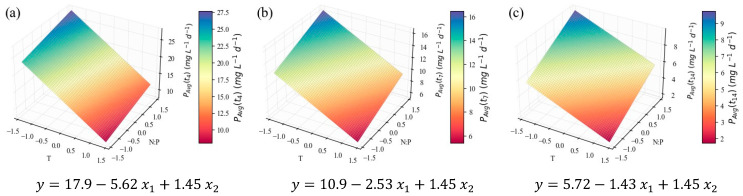
Surface response plots for the average TP productivity (mg L^−1^ d^−1^) of *C. vulgaris* cultivations varying temperature and N:P ratios at the end of *t*_4_ (**a**), *t*_7_ (**b**), and *t*_14_ (**c**), with respective regression equations below. All independent variables and regression coefficients are presented in coded units.

Finally, based on the compound concentrations, it is helpful to calculate their average productivity at the end of each growth stage. The models representing the results for TP, in mg L^−1^ d^−1^, are depicted in Figure 7.

All models related to TP show the influence of the N:P ratio and temperature variables. The first one, regarding protein content (Figure 5), is poorly fitted, according to the statistical parameters in Table 4; hence, no substantial conclusions can be taken from it. However, when contents are weighed with the biomass concentration values, resulting in volumetric concentrations, the correlation (Table 4) becomes satisfactory (*R*^2^ = 0.78)—taking into account the inherent variability of biological systems; and the linear and quadratic factors of the independent variables (Table 5) are all significant (p≤0.1), with coefficients considerably high in the context of the response variable units. This results in a model with an accentuated curvature (Figure 6), which reaches its optimum point inside the domain—even if with a lower temperature level and a higher N:P level than the central point: $\left(x_{1},\;x_{2}\right)=\left(-0.443,\;0.414\right)$, corresponding to temperature and N:P values of 21.9 °C and 17.1, respectively.

Although the models under scrutiny provide information on the quantities of protein that could be extracted from the suspension if harvested at each point, it is even more interesting to analyze the model for TP average productivity (Figure 7). The coefficients of determination also indicate a good fit (*R*^2^ = 0.76, Table 4). Nevertheless, only the linear effects are relevant for this parameter (Table 5), meaning that the response is once again maximized at the limits of the domain. Based on the negative value for *β*_1_ and the positive value for *β*_2_, the model suggests protein productivity is the highest at the lowest temperature tested, i.e., 18 °C, the highest N:P ratio, i.e., 42.6, and at the end of the fourth day of cultivation. Not enough literature is available to confirm whether the linear or the quadratic hypotheses are closer to reality. However, the general tendencies can be commented on.

The positive correlation (*β*_2_) between the N:P ratio and TP production was somewhat expected. Nitrogen is an essential component of amino acids, so it is logical to state that protein accumulation should increase with the availability of inorganic nitrogen (in this case, in the form of NO_3_^−^) in the growth medium—eventually plateauing when the maximum production capacity of microalgae is attained. Although quantitative comparison is challenging due to the different conditions of the studies, this trend was confirmed more than once for this species. Rodrigues-Sousa et al. [68] improved the protein content of a *C. vulgaris* strain from 12.6 ± 0.8% (*w*/*w*) (when cultivated in Bold’s basal medium, under 32.4 mg N L^−1^) to 21.9 ± 0.2% (*w*/*w*) (using secondary effluents from the dairy industry, with 122.8 mg N L^−1^). Notably, there were various nitrogen sources in the wastewaters (nitrite, nitrate, and ammonium). Another study [69], using urea as the sole nitrogen source, improved protein content of an already performant strain from 22.5 ± 0.7% (*w*/*w*) to 55.7 ± 1.7% (*w*/*w*) by doubling the urea concentration. The biomass productivity was also enhanced by almost twofold, and the protein productivity by more than threefold.

The effect of temperature upon protein production has not been so commonly studied in *C. vulgaris*, and the existing literature is inadequate for direct comparison with the present work. Xu et al. [27] tested low (4 °C) and high (35 °C) temperature regimes, as well as an alternating regime with low and high temperatures for 12 h each, and reported the 35 °C regime to stimulate protein production, up to 24.2% of the cell dry weight. It remains to be known whether intermediate conditions would be even more beneficial. Another study [70] tested a 20–45 °C interval and obtained the ideal crude protein productivity at 35 °C and 40 °C—yet, a thermotolerant *C. vulgaris* strain was used under mixotrophic cultivation. A more comprehensive literature review, comprising other microalgae species [30,50,51,53], supports the conclusion that bulk protein production is generally favored at, or around, ideal growth conditions [16], which proved to be strain-specific. The results support that the biomass productivity models presented before point to the ideal temperature for this strain being located on or below the lower boundary (18 °C) of the chosen interval; the same was found for protein productivity.

So far, only the effects of temperature and N:P ratio, which can be deduced from the shape of the surface plots, have been mentioned. However, for each model, it is also possible to draw comparisons between the subsequent harvesting times and comment on the effect of the growth stage. TP intracellular contents visibly decrease (in Figure 5 and in Table S2 of the Supplementary Material) at later stages of the cultivation, while at the exponential stage (*t*_4_) the biomass is richer in proteins. This was observed previously for *C. vulgaris* [71] and other species [72,73], confirming the association of higher protein contents with higher rates of metabolic activity, typically found in the exponential growth stage. The concentration models in Figure 6 do not seem to present this effect, since the biomass concentration increases along the duration of the cultivation, counterbalancing the protein content decrease. However, in Figure 7, it is evident that the TP productivity is much higher at earlier stages.

**Figure 8:**
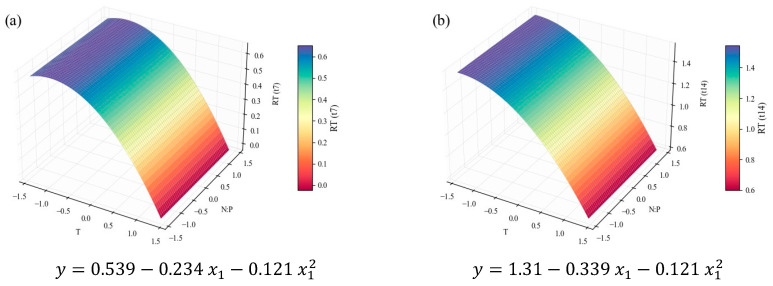
Surface response plots for the relative tendency of the total FA content (% *w*/*w*) of *C. vulgaris* cultivations varying temperature and N:P ratios at the end of *t*_7_ (**a**) and *t*_14_ (**b**), with respective regression equations below. All independent variables and regression coefficients are presented in coded units.

**Figure 9:**
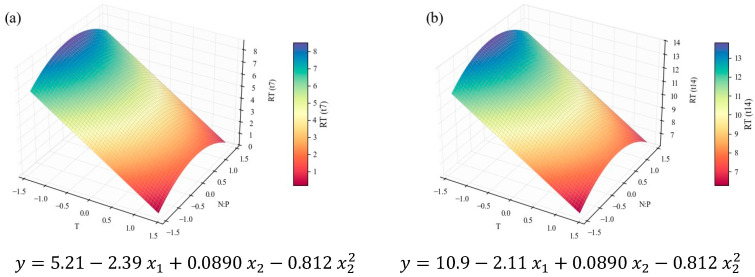
Surface response plots for the relative tendency of the total FA concentration (mg L^−1^) of *C. vulgaris* cultivations varying temperature and N:P ratios at the end of *t*_7_ (**a**) and *t*_14_ (**b**), with respective regression equations below. All independent variables and regression coefficients are presented in coded units.

**Figure 10:**
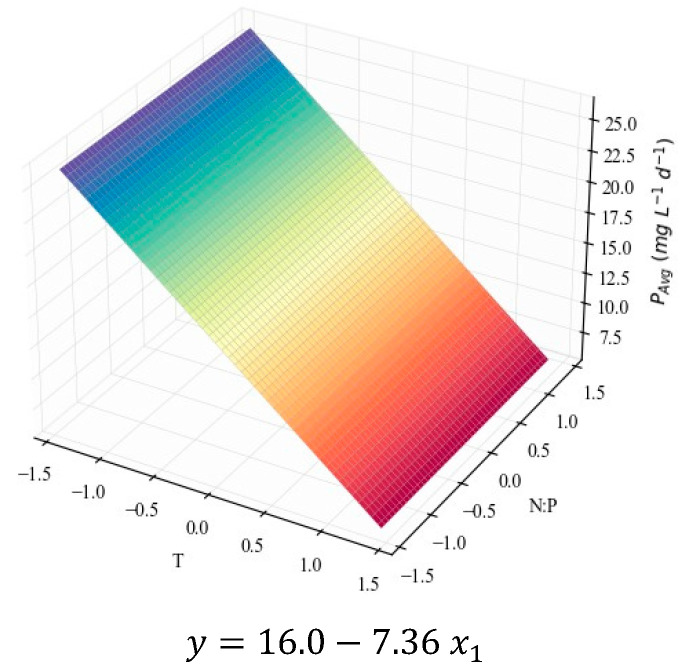
Surface response plots for the average total FA productivity (mg L^−1^ d^−1^) of *C. vulgaris* cultivations varying temperature and N:P ratios, with respective regression equation below. All independent variables and regression coefficients are presented in coded units.

Another major group of macromolecules in microalgae is lipids. In this study, the total FA content was approximated by the sum of all compounds detected by GC analysis of the FA profile. It must be noted that the FA content does not equate to the total lipid content of the microalga, but rather to the fraction that can be subjected to transesterification, which makes it useful for biodiesel production. The following figures represent how these results can be modeled by temperature and N:P variations, according to the planned DoE. Similar to the workflow adopted for TP, Figure 8 represents the *RT* (dimensionless) of the total FA cellular content (% *w*/*w*), Figure 9 the *RT* of the total FA concentration (mg L^−1^) in the culture, and Figure 10 the time-averaged total FA productivity. FAME protocols could not be applied to *t*_4_, but it is expected that, in practice, the responses would be similar to those pertaining to *t*_7_.

Figure 8 and Figure 9 are supported by strong correlations, with *R*^2^ values of 0.86 and 0.92, respectively, while Figure 10 is less so, with an *R*^2^ below the 0.70 level (Table 4). The first two inform on the fact that total FA content in the cells was visibly higher in the stationary stage, suggesting a response to the decrease in nutrient availability. The increase in triacylglycerols (TAGs) content from exponential to stationary growth phases is widely reported [74]. For instance, Klin et al. [75] investigated the cultivation of 15 green microalgae and reported that most of them showed a significant lipid accumulation during the stationary phase.

Regarding continuous variables, the critical observation of all the total FA models together indicates that only the effect of temperature was truly relevant for shaping FA accumulation in this system. Furthermore, the same effect has already been observed for growth and protein accumulation: lower temperatures favor the production of total FA. Converti et al. [28] reported similar patterns: a decrease in the lipid content of *C. vulgaris* from 14.71% (*w*/*w*) at 25 °C to 5.90% (*w*/*w*) at 30 °C. However, most studies point to a different tendency: lipid biosynthesis appears more prevalent at temperatures above the optimum values, as per a stress response [16,27,30,52,53], and the SFA fraction of the FAME profile (analyzed further below) should increase as well to harden the cellular structure [16,53]. Some dependence on N:P ratio was also expected, since lipogenesis is favored under nitrogen limitation or depletion [76], mainly in terms of neutral lipids (richer in SFAs and MUFAs), playing energy storage roles [16,24,38]. Ördög et al. [33] confirmed the mentioned tendencies (both in temperature and N concentration) for three *Chlorella* strains.

As mentioned before, FA content does not equate to the total lipid content of the microalgae. In that order, FA can serve various functions in the cell, depending on the group of macromolecules they are a part of—TAGs, phospholipids, or glycolipids, for instance. Therefore, possible inferences on the cell’s metabolic activities based on FA variation are always indirect.

**Figure 11:**
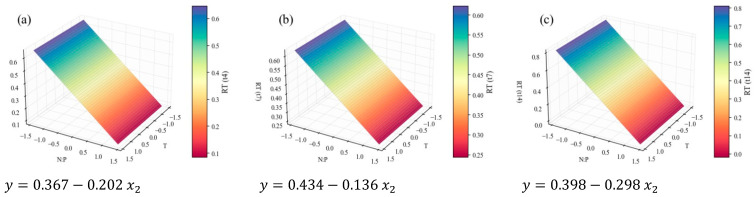
Surface response plots for the relative tendency of the total carbohydrate content (% *w*/*w*) of *C. vulgaris* cultivations varying temperature and N:P ratios at the end of *t*_4_ (**a**), *t*_7_ (**b**), and *t*_14_ (**c**), with respective regression equations below. All independent variables and regression coefficients are presented in coded units. Note: the x and y axes are switched relative to the other plots for better visibility.

**Figure 12:**
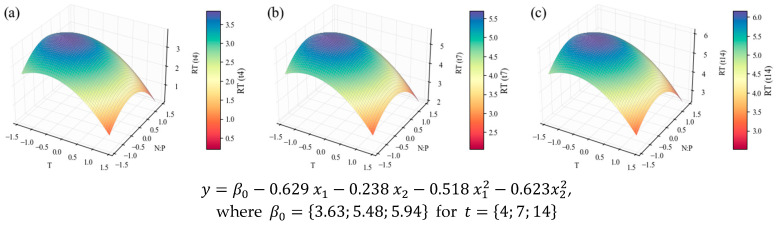
Surface response plots for the relative tendency of the total carbohydrate concentration (mg L^−1^) of *C. vulgaris* cultivations varying temperature and N:P ratios at the end of *t*_4_ (**a**), *t*_7_ (**b**), and *t*_14_ (**c**), with respective regression equations below. All independent variables and regression coefficients are presented in coded units.

**Figure 13:**
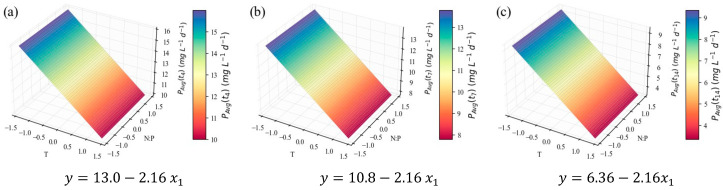
Surface response plots for the average total carbohydrate productivity (mg L^−1^ d^−1^) of *C. vulgaris* cultivations varying temperature and N:P ratios at the end of *t*_4_ (**a**), *t*_7_ (**b**), and *t*_14_ (**c**), with respective regression equations below. All independent variables and regression coefficients are presented in coded units.

TCs were also extracted and quantified for all experiments of the CCD and their growth stages. The information was organized in regression models, and the *RT* of the TC cellular content, their concentration, and TC productivity can be found in Figure 11, Figure 12, and Figure 13, respectively. Unlike previous observations for the other compounds, the carbohydrate model revealing the percentual content tendencies (Figure 11) depends linearly on N:P ratio as the only significant effect, even though its lower *R*^2^ (0.61) suggests involvement of more factors. As the results are combined with the biomass data, highly affected by temperature (as stressed before), the temperature effect surges (Figure 12, *R*^2^ = 0.79) and eventually overrides N:P (Figure 13, *R*^2^ = 0.70). Therefore, conclusions on the carbohydrate trends must be approached with caution.

The lowest N:P ratio tested here can be considered an N-limited regime, with a concentration of 8.8 mg L^−1^; while the standard OECD medium holds 41.2 mg N L^−1^. Although carbohydrate content is rarely a focus among microalgal studies, consensus exists on the accumulation of these compounds over N starvation, in an intermediate carbon flux switch from protein synthesis to eventual lipid accumulation under complete nitrogen depletion [16]. This is the case of recent studies on *Chloroidium saccharophillum* [24] and *Scenedesmus obliquus* [77], while *Chlorella* sp. AE10 showed higher contents under phosphorus starvation [20]. Ikaran et al. [78] also observed this trend for *C. vulgaris*, and a complementary gene expression study revealed that it was not due to the upregulation of carbohydrate metabolism genes, but instead to carbon and nitrogen fixation and triglyceride synthesis genes.

While the present results do not underscore a clear influence of temperature, this could be attributed to the narrow window of observation chosen; it is possible that below the optimum growth temperature (reaching cold stress), a higher carbohydrate content might be recorded, due to the structure-protective effects of these molecules [16]. For instance, Chauhan et al. [30] managed a steady increase from 34.5% (*w*/*w*) to 44.6% (*w*/*w*) of TC, when lowering the cultivation temperature of *Micractinium pusillum* from 35 to 15 °C, and Xu et al. [27] obtained a TC content of 41.3% (*w*/*w*) with *C. vulgaris* under 4 °C, as opposed to 26.3% (*w*/*w*) under 35 °C.

**Figure 14:**
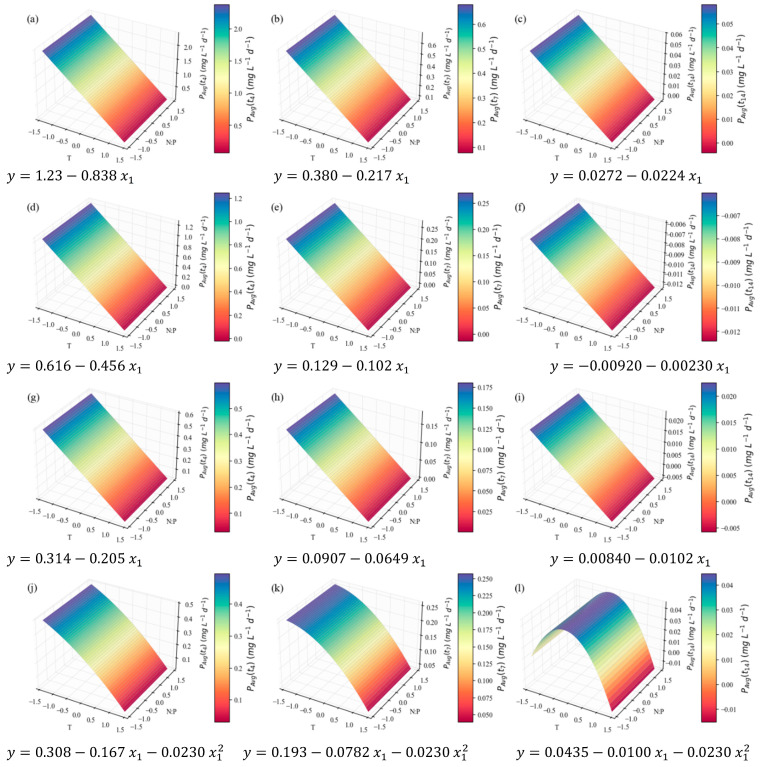
Surface response plots for the total pigments (**a**–**c**), chlorophyll a (**d**–**f**), chlorophyll b (**g**–**i**), and carotenoids (**j**–**l**) productivity (mg L^−1^ d^−1^) of *C. vulgaris* cultivations varying temperature and N:P ratios at the end of *t*_4_, *t*_7_, and *t*_14_ (subsequent columns), with respective regression equations below. All independent variables and regression coefficients are presented in coded units.

Finally, the microalgal composition in the major pigments was duly analyzed. Chlorophylls and carotenoids are pigments of the photosynthetic membranes, crucial for light absorption and photoprotection. Although they belong to the group of isoprenoid lipids [79], their trends should be addressed separately. After extraction, they were quantified via colorimetric methods; in view of their different absorbance spectra, chlorophyll a, b, and carotenoids were measured individually and then added for the total pigments estimation. For a more straightforward and accurate result treatment (due to the small magnitude of several values), only the final productivity (mg L^−1^ d^−1^) of these compounds is presented here. However, it was calculated in the same way from the initial % *w*/*w* content results (see the Supplementary Material, Tables S5–S8). Figure 14 presents the models that better fit the productivity in total pigments, chlorophyll a, chlorophyll b, and carotenoids (in subsequent rows).

It is worth noting that all models representing pigment productivity provide excellent fits, with coefficients of determination above 0.92, and good predictive ability. In this case, the general inverse relationship between temperature and the response variable coincides with the results of the major macromolecule groups—a trait to be expected owing to the high prevalence of the biomass concentration values in productivity calculations. The same argument explains the visibly higher productivity for shorter cultivation times. However, a slight shift is noticeable with carotenoids (Figure 14j–l)—for which the maximization of the curve seems to occur at higher temperatures, especially when the whole cultivation period is encompassed (harvesting at day 14). Carotenoid production, mainly of secondary carotenoids, can indeed be enhanced at temperatures above the ideal ones and at the stationary stage, since they have antioxidant functions and can help fight oxidative stress caused by ROS. In the previously mentioned study [30], an increase and eventual maximum in the carotenoid content of *M. pusillum* from 15 to 35 °C was found. Ma et al. [51] reported distinct behaviors among primary and secondary carotenoids, with the latter increasing at higher temperatures and the former showing a preference for ideal growth temperatures. This is generally also the case for chlorophylls [30,32], which are primary metabolites.

Table 4 depicts data that permits comparison of all models presented thus far, regarding data fitting criteria and optimal points. When leveling out all the points where the different response variables are optimized, the only consensus seems to be that the lowest temperature of the interval (18 °C) and harvesting on the 4th day of cultivation maximize the productivity of biomass and of all the extracted compounds. However, this study had the N:P ratio as a dimension as well, so the TP $P_{Avg}$ optimal point, where N:P is at its maximum, will be used for further considerations—namely, discrimination of compound profiles. In fact, when considering the application of *C. vulgaris* for the food industry (which could be the case in cultivation setups similar to the current one), under controlled conditions and an aseptic environment, TP could be the most interesting compound to maximize, thus supporting the potential of these microalgae as an alternative protein source.

In Table 5, all regression coefficients and their corresponding significances can be found, thus allowing for comparison of the studied factor effects upon the different response variables. As a general observation, it should be mentioned that the $x_{1}x_{2}$ factor, i.e., the linear interaction between the two continuous independent variables, was not significant for any of the models. This means that the influence of temperature on the growth and biochemical composition of our microalgae was not modeled by the N:P ratio, or vice versa.

As a consequence of the chosen result treatment, it can be perceived that the *β*_1_ coefficients for TP, total FA, and TC always increase in absolute value from the ‘Content *RT*’ to the ‘Concentration *RT*’ models, and finally to the productivity models. This is due to the strong influence of temperature upon biomass growth and productivity, which determines that all the other results dependent on biomass values (which is the case of volumetric concentrations and productivities, but not of the initial contents) are influenced by it as well.

### 2.3. Biochemical Composition—Profiles

Besides colorimetric analyses, a fraction of the biomass was subjected to gas chromatography (GC) and high-performance liquid chromatography (HPLC) analyses; the information about each sample composition in terms of FA, carotenoids, and amino acids was duly extracted. This can help assess the value of the microalgae being produced for various markets. In particular, they are crucial specifications when developing microalgal products for human nutrition.

For the sake of bookkeeping, the individual correlations of each molecule with variations in T and N:P ratio will not be explored here. Still, the values each compound assumes for E1 through E12 experiments are presented as Supplementary Material (Tables S9–S40), and statistically significant comparisons were performed. In this section, the focus will be on the observed ranges for each compound throughout the entire study. Additionally, the values observed at E7 will be highlighted, since this is the experimental point that combines a lower temperature (20 °C) with a higher N:P ratio (27) and, thus, can represent the previously chosen optimal point: $\left(x_{1},\;x_{2},t\right)=(-\sqrt{2}$ ^†^$,\;\sqrt{2}$ ^†^,4).

#### 2.3.1. FA Profile

**Figure 15:**
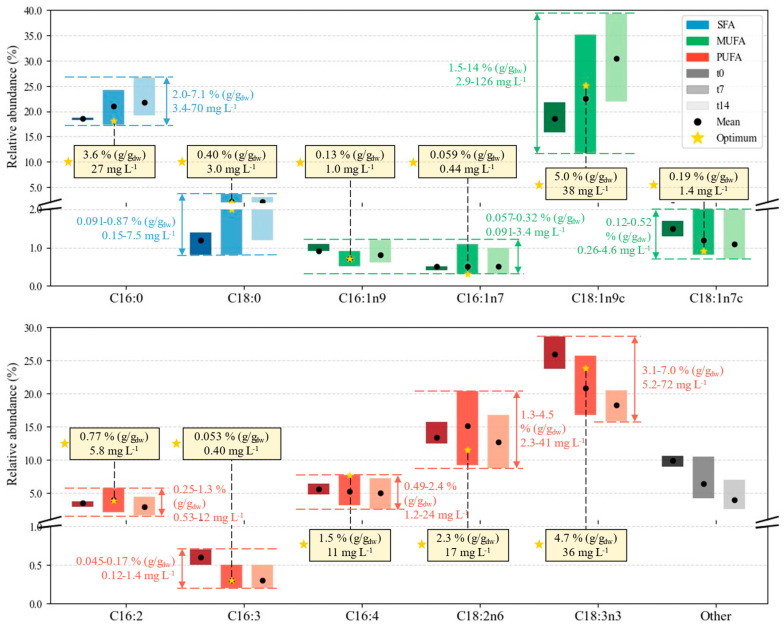
Variations of the FA profile of *C. vulgaris* across the entire experiment. Each bar spans the entire range of percentages (relative to the total FA content) observed in the 12 experiments. Adjacent bars of different shades represent time points 0, 7, and 14. SFA, MUFA, and PUFA categories are grouped by colors, and grey bars represent other/non-identified FAs in the spectrum. ●—mean value; ★—value at optimum point (signaled at *t*_7_ because the FA profile was not assessed in the *t*_4_ samples, but the approximation was considered valid since more variation of lipidic content is expected in the stationary stage).

Figure 15 provides a schematic representation of the FA profile. The percentages of each FA relative to the total FA content were computed for each sample. It is also valuable to group fatty acids according to the number of double bonds in their structure: SFA (0), MUFA (1), and PUFA (≥2) [80,81]. The bars in Figure 15 provide visual comparisons of the fractions of each FA in the total profile; for reference, their sum (i.e., the total FA content) ranges from 11 to 36% (*w*/*w*). Depending on the biomass quantities, this can mean fatty acid concentrations of 1.85–357 mg L^−1^ in the culture. The colored labels next to the bars feature the interval of cellular contents and cultured concentrations of each FA, while yellow labels highlight the specific values at the E7 point. The maximum productivity for FA, reached at *t*_7_ in cultivation E5 (20 °C, N:P = 3), was 31 ± 2 mg L^−1^ d^−1^.

The profile displayed in Figure 15 makes a few patterns apparent. First, the relative size and positioning of the bars reveal that C18:1n9c (oleic acid), a MUFA, is the fatty acid generally more prominent, and that it can be pushed to higher levels (almost up to 40%) via manipulation of cultivation conditions. Moreover, its portion tends to increase with cultivation time. The second largest fraction is shared between the SFA C16:0 (palmitic acid) and the PUFA C18:3n3 (alpha-linoleic acid, or ALA). While C16:0 shares the oleic acid tendency to increase at later growth stages, ALA exhibits the opposite relationship with cultivation time. ALA is followed by C18:2n6 (linoleic acid, or LA) in abundance among the PUFAs analyzed. The observations of the major components of each FA category, the general dependence of cultivation time, and the dominance of fatty acids with 16 and 18 carbon chain lengths are all consistent with data by Kheibari et al. [82]. Several other compounds fall below the 5% mark: C18:0 (stearic acid), an SFA; C16:1n9, C16:1n7, C18:1n7c (MUFAs); and C16:2, C16:3 (PUFAs). Despite having less influence on the gross profile definition, all of them are marked by broader bars for *t*_7_ and *t*_14_, emphasizing that T and N:P can influence their variability.

The higher abundance of C18:1n9c and C16:0 agrees with the literature for the same microalgal species, and within the expected range: (i) Rodrigues-Sousa et al. [68] reported 26.5–39.9% and 25–30.3%, respectively, when varying N contents from dairy effluents; (ii) Converti et al. [28] studied temperature and N contents and reached ca. 60% in palmitic acid and 38% in oleic acid. Ördog et al. [33] confirmed this tendency for three *Chlorella* strains over a range of temperatures and N concentrations, with C16:0 ranging from 22.1 to 24.0% and C18:1n9c from 21.1 to 21.4%. Moreover, hierarchical cluster analysis unfolded these two FAs as the most susceptible to variations in cultivation conditions.

Cumulatively, PUFAs are the most significant fraction of FA, followed by MUFAs and SFAs. This has also been observed previously for this species [33,68,83]. Given their importance as primary structural constituents of the cell membrane, and the substantial benefits of PUFAs for human health (including the regulation of blood pressure, glucose levels, inflammatory reactions, and the nervous system [84]), this is strongly indicative of the potential of cultivating this microalgae for food and nutraceutical products [12,85]. Interestingly, the two most prevalent PUFAs are essential fatty acids: LA is an essential ω-6 and ALA an essential ω-3. As per the definition of any essential nutrient, they cannot be synthesized de novo sufficiently fast for a regular physiological role; hence, they must be included in the diet.

#### 2.3.2. Carotenoid Profile

**Figure 16:**
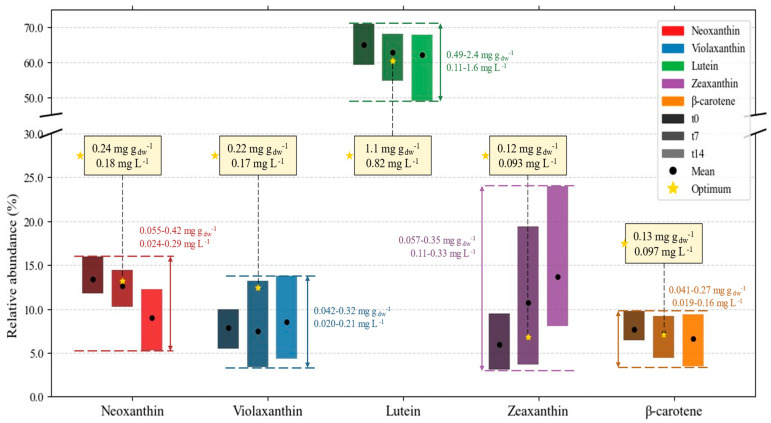
Variations of the carotenoid profile of *C. vulgaris* across the entire experiment. Each bar spans the entire range of percentages (relative to the sum of the 5 presented carotenoids) observed in the 12 experiments. Adjacent bars of different shades represent time points 0, 7, and 14. ●—mean value; ★—value at the optimum (signaled at *t*_7_ because the carotenoid profile was not assessed in the *t*_4_ samples, but the approximation was considered valid since more variation of lipidic content is expected in the stationary stage).

In a way similar to how the data were treated for the FA profile, Figure 16 displays the variation of the five carotenoids analyzed by HPLC: neoxanthin, violaxanthin, lutein, zeaxanthin, and β-carotene. Their sum amounts to intracellular contents of 0.83–3.7 mg g_dw_^−1^ and culture concentrations of 0.19–2.3 mg L^−1^. The maximum carotenoid productivity obtained was 0.26 ± 0.09 mg L^−1^ d^−1^ at *t*_7_ of E3 (32 °C, N:P = 9).

An immediate comment on the data in Figure 16 is the relative abundance of lutein, spanning from around 50 to 70% of the content considered to be carotenoids. Several authors confirm that *C. vulgaris* is a promising source of lutein [55,86,87], so that it is often studied separately in microalgae [87,88]. The first reason for this is that microalgae not only have a higher lutein content (3.4–7.6 mg g_dw_^−1^) than marigold flowers (0.22–0.98 mg g_dw_^−1^), the current primary source of its commercial production, but are also a cost-effective alternative to these flowers, overriding setbacks such as the need for arable land and seasonal limitation [87]. The contents reported in this study (0.49–2.4 mg g_dw_^−1^) are slightly lower, but still meet this requirement. The second reason lies in the added value of this compound. Even though mammals are unable of its de novo synthesis, no carotenoid is considered an essential nutrient to date, as it neither plays a direct role in vital metabolic pathways nor does its absence exclusively indicate deficiencies or likelihood of chronic diseases. Therefore, there are no formal recommendations for their daily intakes. However, there is a debate on whether lutein and zeaxanthin should be recognized as conditionally essential [89,90]. Besides being used as food colorants, these carotenoids are emerging as supplements or nutraceuticals, due to their role in reducing oxidative damage in the retina, treating ocular diseases such as cataracts or age-related macular degeneration (which affects 196 million people globally), and preventing cancer. Another example of a carotenoid used for specific therapy is β-carotene, which mitigates the effects of light in erythropoietic protoporphyria patients [91,92].

Even though the relative abundance bars do not allow for these conclusions, the more detailed data in the Supplementary Material (Tables S20–S24) reveal a decline in the contents and productivity of all five carotenoids from day 7 to day 14—that is, in the stationary growth stage. Pinto et al. [55] observed similar declines in neoxanthin, lutein, and β-carotene over 14 days of cultivation of the same strain. The authors reported decreases in violaxanthin as well, albeit less steeply, and no clear dependence on time for zeaxanthin. Another recent study on *C. vulgaris* [93] highlights that different isomers of the carotenoids observed here can evolve differently along the growth stage. Ultimately, it can be observed that all carotenoids included in this study are primary carotenoids, i.e., associated with growth and normal metabolic functions. Others, such as astaxanthin and canthaxanthin, would more easily be expected to increase at later stages of cultivation [94].

It should still be mentioned that, similarly to what was observed for fatty acids, each carotenoid content range increases at the end of the exponential and the stationary stages—meaning that the tested variables are relevant for shaping the composition. The extent of this variability is greater for lutein and zeaxanthin, as previously pointed out by Hynstova et al. [95], underscoring the interest in manipulating these two compounds via cultivation conditions. However, these authors also mention that lutein and zeaxanthin are stereoisomers, and the possibility of interference in quantification by one another might account for some of this variation.

#### 2.3.3. Amino Acid Profile

**Figure 17:**
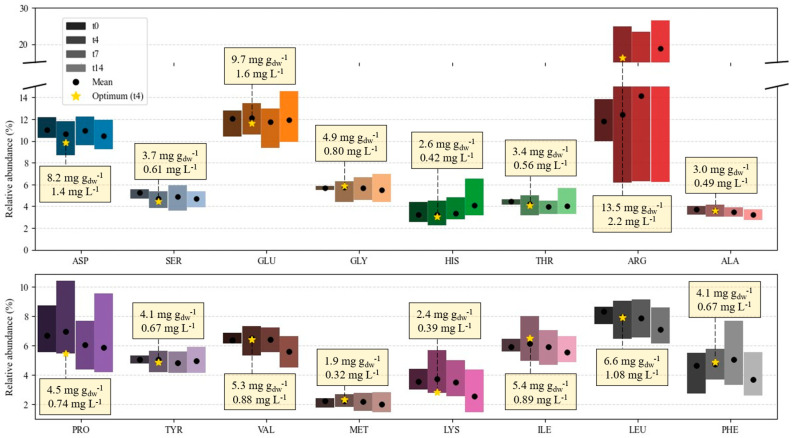
Variations of the amino acid profile of *C. vulgaris* across the entire experiment. Each bar spans the range of percentages (relative to the sum of the 16 amino acids) observed in the 12 experiments. Adjacent bars of different shades represent time points 0, 4, 7, and 14. ●—mean value; ★—value at optimum.

The amino acid profile, based on the HPLC analyses, is represented in Figure 17. The total amino acid content represents 29–138.4 mg g_dw_^−1^ and can be found in concentrations of 14–93 mg L^−1^ in suspension. The microalgae reached maximum amino acid productivities of 11.4 mg L^−1^ d^−1^ by the end of the seventh day in E12 (one of the central point replicates of the DoE).

Essential amino acids (EAAs) are defined as those that must be provided in the diet, since their carbon skeletons are not (or insufficiently) synthesized de novo. EAA deficiencies, with a reduced rate of protein synthesis in cells and tissues as a direct consequence, are syndromes that cause severe health problems, such as low appetite and vomiting, low nutrient absorption, reduced oxygen transport, and emotional disorders. They are a highly impactful nutritional problem, affecting more than half of home-bound elderly in the United States. HIS, ILE, LEU, LYS, MET, PHE, THR, TYR, and VAL, which constitute EAAs for all animals, are all present in the profile of this *C. vulgaris* strain, as perceived by inspection of Figure 17 [96]. This richness and relatively even distribution in EAAs underscore the potential of this microalgae as a functional food product.

The content in some of the most prominent amino acids (ASP, PRO, VAL, LYS, ILE, LEU) clearly decreases at the day 7 and day 14 observations. On the contrary, ARG contents tend to increase on average, although the total variability is similar from days 4 to 14. This variability can mean more than 25% of the total amino acids are ARG content. Although not considered an EAA for adult humans, its absence in the diet after nine days comes at the cost of up to 90% spermatogenesis and sperm viability [97]. It is an important amino acid—often taken orally to stimulate the production of NO (a molecule with important functions in the circulatory system) and known to enhance fertility and improve metabolic profiles [96,98]. Furthermore, birds, fish, and some mammals (e.g., cats) are incapable of de novo ARG synthesis, thus opening possible market opportunities for feed applications of ARG-rich products [96]. Even though ARG is recognized as one of the major amino acids in *Chlorella* and *Chlorella*-derived products [98], most studies involving marine microalgae highlight GLU and ASP as the main profile components [99]. They also occur at high proportions in our study, from about 10 to 15%. Glutamate is important for the organoleptic properties of the product, being the chief contributor to the umami taste, and both this amino acid and aspartate benefit muscular function as well [98,99]. Among the remaining amino acids, some may be mentioned for their commercial interest, whether due to pharmaceutical applications (e.g., LYS is used to treat herpes simplex) or because they may be necessary for animal feeding and supplementation. For instance, birds have limited to null synthesis of PRO from various pathways (from arginine, glutamate, and glutamine), because they lack the required enzymes. Poultry and young pigs need dietary GLY, since the utilization rate of this amino acid surpasses that of its synthesis. Fish need both PRO and GLY supplementation [97,100].

## 3. Materials and Methods

### 3.1. Microalgae

The microalgae *C. vulgaris* CCAP 211/11B used in this study was provided by Culture Collection of Algae and Protozoa (CCAP, Scotland, UK). It was inoculated under axenic conditions in Erlenmeyer flasks and grown in the modified OECD (Organization for Economic Cooperation and Development) growth medium, with the following composition (per liter): 250 mg NaNO_3_, 12 mg MgCl_2_·6H_2_O, 18 mg CaCl_2_·2H_2_O, 15 mg MgSO_4_·7H_2_O, 45 mg KH_2_PO_4_, 0.08 mg FeCl_3_·6H_2_O, 0.1 mg Na_2_EDTA·2H_2_O, 0.185 mg H_3_BO_3_, 0.415 mg MnCl_2_·4H_2_O, 3 × 10^−3^ mg ZnCl_2_, 1.5 × 10^−3^ mg CoCl_2_·6H_2_O, 0.01 × 10^−3^ mg CuCl_2_·2H_2_O, 7 × 10^−3^ mg Na_2_MoO_4_·2H_2_O, and 500 mg NaHCO_3_ [3,55]. The stock culture was kept at room temperature under a continuous light supply of 50 μmol m^−2^ s^−1^ and stirred at 120 rpm using a Unimax 1010 orbital shaker (Heidolph, Schwabach, Germany).

### 3.2. Inoculation and Culture Conditions

Inoculum biomass was cultivated in 1 L Schott borosilicate glass bottles inside a Panasonic MLR-352 PE (PHC, Breda, The Netherlands) climate chamber, at 25.0 °C, under a continuous light supply of 132 µmol m^−2^ s^−1^, provided by a horizontal LED lamp. Homogenization and CO_2_ supply were both guaranteed by continuous pumping of atmospheric air at 1.0 vvm with air pumps (Sicce Airlight 3300, Pozzoleone, Italy), filter-sterilized through 0.22 µm nylon membrane filters (Specanalitica, Cascais, Portugal).

Experimental batch cultivations started at ca. 0.20 ± 0.02 g_dw_ L^−1^, and were carried out in 7 L benchtop bioreactors (Fermentec FMT DSA-D series, Cheongju-si, Republic of Korea) [101], using a working volume of 3 L. The cultures were grown for 14 days under continuous light at ca. 55 µmol m^−2^ s^−1^, automatic temperature control via a cooling jacket and a heating system, pH control (which was maintained within 7–7.5 range), stirring at 150 rpm by 6-flat-blade Rushton turbines (corresponding to a 0.39 m s^−1^ tip speed), and 1.0 vvm aeration with filter-sterilized atmospheric air using a ring sparger. Nitrogen and phosphorus availability were adjusted according to the experimental design by varying NaNO_3_ concentration in the growth medium. All other nutrients were provided in the quantities given by the modified OECD growth medium.

### 3.3. Design of Experiments and Response Surface Methodology

The experimental responses of *C. vulgaris* growth, biomass productivity, and production of bioactive compounds, along with its growth stages, were subject to RSM. This approach consists of fitting a second-order polynomial equation to the experimental data, which should describe their behavior and allow for predictions, including finding optimal points on the continuous response surfaces generated. This model can be generically defined by Equation (1), where *y* is the predicted response variable, $\beta_{0}$, $\beta_{i}$, $\beta_{ij},$ and $\beta_{ii}$ are regression coefficients obtained by least squares regression, with indices referring to the independent factors, $x_{i}$ are coded values of the independent variables, and ε is the random error of the estimated response; $x_{i}$ can be computed from the true values of the independent variables ($Z_{i}$) according to Equation (2), where $Z_{0}$ is the value of $Z_{i}$ at the centre point and ${∆Z}_{i}$ corresponds to half of the range typical of the RSM design at play [34,102]:

(1) $$y=\beta_{0}+\sum_{i=1}^{k}\beta_{i}x_{i}+\sum_{i=1}^{k-1}\sum_{j=i+1}^{k}\beta_{ij}x_{i}x_{j}+\sum_{i=1}^{k}\beta_{ii}x_{i}^{2}+\varepsilon$$

(2) $$x_{i}=\frac{Z_{i}-Z_{0}}{∆Z_{i}},\;i=1,2,3,\ldots ,\;N$$

The conditions defining the experimental points of the present study were based on a rotary CCD (see Table 1). The central point was repeated four times to assess the intrinsic variability.

The JMP (Version 18.2.1, SAS Institute; Cary, NC, USA) and Minitab (Version 22.3.1.0, Minitab Inc., State College, PA, USA) software were used to process the results of RSM. The models were subjected to analysis of variance, and the parameters were considered statistically significant at a *p*-value below 0.05, associated with a 95% confidence level. The non-significant parameters were removed, and the adjusted model performance was evaluated considering coefficients of determination (*R*^2^), the adjusted *R*^2^, the predicted *R*^2^, and the root mean square error (RMSE). Finally, the optimal point, i.e., the response maximization and corresponding conditions, was calculated.

### 3.4. Biomass Growth Monitoring

The culture OD at 680 nm was measured daily in a UV-1800 Shimadzu spectrophotometer (Shimadzu Europe, Duisburg, Germany), with duplicate readings. Using a calibration curve, OD readings were then converted to biomass concentrations (*X*, in mg_dw_ L^−1^). All relevant calibration curves can be found in the Supplementary Material (Figures S2–S6). Microalgal growth rates (*μ*, d^−1^) were calculated during the exponential phase using Equation (3), where $X_{f,exp}$ and $X_{0}$ are the microalgal cell concentrations (mg_dw_ L^−1^) in the final ($t_{f,exp}$, d) and initial instant ($t_{0}$, d) of the exponential growth phase, respectively. Another growth parameter, the average biomass productivity ($P_{X,avg}$, mg_dw_ L^−1^ d^−1^) was also calculated via Equation (4), where $X_{0}$ and $X_{f}$ are the biomass concentrations (mg_dw_ L^−1^) at the beginning ($t_{0}$, d) and at the end ($t_{f}$, d) of each experiment:

(3) $$\ln\frac{X_{f,exp}}{X_{0}}=\mu \left(t_{f,exp}-t_{0}\right)$$

(4) $$P_{X,avg}=\frac{X_{f}-X_{0}}{t_{f}-t_{0}}$$

### 3.5. Nutrient Consumption Monitoring

Under axenic conditions, 5 mL samples of each experiment were collected on days 0, 1, 2, 4, 7, and 14; these points were chosen to represent the nutrient removal curve [3]. The samples were centrifuged in a GZ-1580R centrifuge (Gyrozen, Daejeon, Republic of Korea) at 3075× *g* for 10 min, at 4 °C. Before analysis, the supernatant was filtered through 0.22 µm cellulose acetate membrane syringe filters (Whatman plc, Maidstone, UK). Nitrate–nitrogen (NO_3_-N) measurements followed the procedure proposed by Collos et al. [103], while phosphate–phosphorus (PO_4_-P) analyses were performed according to the protocol formerly described by Lee et al. [104]. The absorbance values were converted to nutrient concentrations using previously prepared calibration curves. Nitrogen and phosphorus removal by microalgae was then quantified by parameters removal efficiency (*RE*, %) (Equation (5)) and average removal rate (*RR*, mg L^−1^ d^−1^) (Equation (6)). In these equations, *S*_0_ and *S_f_* (in mg L^−1^) represent the nutrient concentrations at the beginning and end of each experiment, respectively, and (*t_f_* − *t*_0_) represents its duration in days (d):

(5) $$RE=\frac{S_{0}-S_{f}}{S_{0}}\times 100$$

(6) $$RR=\frac{S_{0}-S_{f}}{t_{f}-t_{0}}$$

### 3.6. Harvesting and Lyophilization

On days 4, 7, and 14 (*t*_4_, *t*_7_, and *t*_14_) of each experiment, a specific culture volume was harvested: 0.5 L, 1.5 L, and 1 L, respectively. Additionally, on inoculation days (*t*_0_), some inoculum culture was harvested as an anchor comparison point. The suspensions were centrifuged in a GZ-1580R centrifuge at 3745× *g* for 10 min, at 4 °C. The pellet was lyophilized using a 6K Benchtop freeze dryer (VirTis, New York, NY, USA) under vacuum conditions and duly stored.

### 3.7. Extraction and Analyses: General Biochemical Composition

Lyophilized biomass was subjected to comprehensive biochemical profiling, according to the protocols already optimized by Esteves et al. [16]. All extractions described below were performed in duplicate.

Protein quantification was based on Lowry et al. [105]. The absorbance at 500 nm and 750 nm was measured in polystyrene cuvettes, and the protein content was assessed via bovine serum albumin (BSA) calibration curves.

A protocol adapted from Clément-Larosière et al. [106] was used to quantify photosynthetic pigments. The absorbance of the extracts was read at 665 nm, 652 nm, and 470 nm with a glass cuvette, and the total contents of carotenoids and chlorophylls (a, b and a + b) were estimated by the Lightenthaler equations [79].

Carbohydrate extraction followed a methodology well described in the literature [107,108], based on sulfuric acid (H_2_SO_4_) digestion. The absorbance was read at 490 nm in a glass cuvette. The calibration curve used a standard glucose solution.

Every result from the colorimetric methods was converted into concentrations (% *w*/*w*), and the content of each nutrient in the biomass was also ascertained. To more effectively treat the results, they were normalized by the inoculum culture values, according to Equation (7), and only the dimensionless relative tendency (*RT*) values at *t*_4_, *t*_7_, and *t*_14_ were used for fitting by the models. Values around 0 mean no change from the characteristics of the inoculum, while positive and negative values denote increases and decreases, respectively:

(7) $$RT=\frac{Content\;\left(t\right)-Content\;\left(t_{0}\right)}{Content\;\left(t_{0}\right)}$$

Biomass concentration data allows for calculating the concentrations (in mg L^−1^) of each product in the medium at the selected time points. These quantities can be normalized in the same way (Equation (7)) or used to calculate average productivities ($P_{avg}$) at the end of each growth stage, as shown by Equation (8):

(8) $$P_{avg}=\frac{Concentration\;(t)-Concentration{\;(t}_{0})}{t-t_{0}}$$

### 3.8. Extraction and Analyses: Profiles

Simultaneous cell disruption and carotenoid extraction followed the procedure previously used by Pinto et al. [55], based on bead-beating in a Precellys^®^ Evolution Touch homogenizer (Bertin Technologies, Montigny-le-Bretonneux, France) using acetone as single solvent [109]. A known volume of trans-β-Apo-8′-apo-carotenal (170 mg L^−1^; ExtraSynthase, Genay, France) was used as an internal standard. All extractions were performed in triplicate. Samples were centrifuged using a GZ-1580R centrifuge at 3075× *g* for 10 min at 5 °C. The supernatant was filtered through 0.45 µm PTFE filters (VWR) and dried under a gentle nitrogen stream using an SBHCONC/1 Sample Concentrator (Stuart, Stone, UK). The dry residue was resuspended in a mixture of acetone and ethyl acetate (9:1, *v*/*v*) and injected into a HPLC system, a Waters Alliance 2695 (Waters, Milford, MA, USA), using a 4 × 250 mm Purospher Star RP-18e (5 μm) column (Merck, Darmstadt, Germany) as described by Pinto et al. [55]. Spectral data were collected over the range of 250 to 750 nm for all peaks. The pigments were identified by retention time and spectra comparison with authenticated HPLC-grade standards, and quantified via calibration curves (see Supplementary Material, Figure S5).

Total amino acid profile quantifications followed the method by Cohen and Deantonis [110], with some modifications. Briefly, 20 mg of dry biomass and 3 mL of hydrochloric acid solution (HCl, 6 M) containing 0.5% (*w*/*v*) phenol were inserted into a 10 mL glass hydrolysis tube. The tubes were saturated with nitrogen gas and sealed. Hydrolysis proceeded at 110 °C for 24 h. A 0.2 mL portion of the hydrolysate was neutralized with NaOH 6 M and borate buffer (0.1 M), filling the remaining volume up to 1 mL, in Eppendorf tubes. The samples were centrifuged for 1 min at 16,200× *g* in a Sorvall Legend Microlitre centrifuge (ThermoFisher Scientific, MA, USA), and 5 μL of the supernatant was combined with the components of an AccQ-Tag derivatization kit (Waters^TM^, Milford, MA, USA). The mixture was incubated at 55 °C for 10 min. All hydrolyses were performed in duplicate, and all samples obtained were derivatized in duplicate, too. After derivatization, 5 µL of each sample was injected into a Waters Alliance 2695 fitted with an AccQ-Tag Amino Acids C18 Column (4 µm, 150 × 3.9 mm) (Waters, Wexford, Ireland). The fluorescence detector was set to *λ_Ex_* = 250 nm and *λ_Em_* = 395 nm (PMT = 100). Spectra were scanned in the range of *λ* = 210–600 nm. Chromatographic separation was performed at 37.0 °C and a 1 mL min^−1^ flow rate, using a combination of three eluents: a patented aqueous buffer (eluent A, Waters, Milford, MA, USA), milli-Q water (phase B), and acetonitrile (eluent C). The following elution was found optimal: 0 min (A, 100%); 0.5 min (A, 99 percent; B, 1 percent); 18 min (A, 95%; B, 5%); 19 min (A, 91%; B, 9%); 28 min (A, 83%; B, 17%); 35 min (B, 60%; C, 40%); and 38 min (A, 100%) for a total analysis duration of 47 min. The Amino Acid Food and Feed Standard Kit (Waters, Milford, MA, USA) was used for calibration. It contains 21 amino acids, namely alanine (ALA), α-aminobutyric acid (AABA), arginine (ARG), aspartic acid (ASP), cystein (CYS), cysteic acid (CYA), glutamic acid (GLU), L-glycine (GLY), L-histidine (HIS), L-isoleucine (ILE), L-leucine (LEU), lysine (LYS), methionine (MET), methionine sulfone (MetO), phenylalanine (PHE), L-proline (PRO), L-serine (SER), taurine (TAU), L-threonine (THR), L-tyrosine (TYR), and valine (VAL). Seventeen of those amino acids were quantified (CYS was not detected in all assays performed) using a five-point calibration curve (ranging from 5 to 250 μM of each analyte) with D-norleucine serving as internal standard (IS) [55].

Lipid characterization followed a GC method, pre-tested and selected for better compatibility with the samples and equipment and adapted from Pagels et al. [111]. Shortly, 30 mg of lyophilized biomass was mixed with 50 µL of the antioxidant BHT (butylated hydroxytoluene, 0.2% in methanol), 100 µL of triundecanoin as internal standard, and 2 mL of acetyl chloride (5:95 (*v*/*v*) in methanol). The mixture was incubated for 1 h at 80 °C, with agitation at 20 min intervals. After cooling to room temperature, 1 mL of a 1% NaCl solution and 1 mL of GC-grade hexane were added, followed by centrifugation (3220× *g*, 5 min) in a Heraeus Sepatech Labofuge AE centrifuge (Heraeus Sepatech GmbH, Osterode, Germany). A volume of 900 µL of the upper phase was recovered and dehumidified with anhydrous sodium sulphate (Na_2_SO_4_). The supernatant was finally transferred to injection vials. Samples were analyzed in triplicate. The chromatography analysis of the fatty acid profile was run in an Agilent Technologies 7890A chromatograph equipped with FID detection and an Agilent Select FAME column (50 m × 250 µm) for 32 min. The injector and detector temperatures were kept at 250 °C and 270 °C, respectively. The temperature program was as follows: initial column temperature of 160 °C, increased to 190 °C at a rate of 2 °C min^−1^, and further increased to 240 °C at 25 °C min^−1^. The final temperature was kept for 10 min. Each sample with a volume of 1 µL was injected at a split ratio of 1:50, and helium was used as a carrier gas.

### 3.9. Statistical Analysis

The quantitative responses studied were growth rate, biomass productivity, nutrient (phosphate and nitrate) removal efficiency, mass removal extent and rate, carotenoid, total amino acid and lipid profiles, total FA, TP, total pigments, and TC, as well as the evolution of these contents across the different stages of 14-day microalgal cultivations. All results are presented as mean values, with their corresponding standard deviations (SD), in the cases where *n* ≥ 3, or standard errors (SE) where *n* ≤ 2. Data treatment was performed with Minitab and Excel. Statistical differences were determined where *n* ≥ 3 by two-way analysis of variance (ANOVA) with interaction, followed by Tukey’s HSD (honestly significant difference) test at *p* < 0.05. Values sharing the same letter are not significantly different. The plots in the Section 2 were created using the Seaborn (Version 0.13.2) and Matplotlib (Version 3.10.0) libraries in Python (Version 3.10.0).

## 4. Conclusions

The main output of this study is a versatile and multifaceted database on the response of *C. vulgaris* CCAP 211/11B to variations in temperature and N:P ratio, defined by a CCD. Twelve 14-day experiments were conducted, and fractions of the biomass were harvested on days 0, 4, 7, and 14.

Biomass productivity was optimal (122.27 mg_dw_ L^−1^ d^−1^) at day 4, the end of the early-exponential stage at the lowest temperature level. The N:P level apparently showed no influence upon this response. The intracellular content in TP depended on both T and N:P factors, and the productivity in TP attained its maximum (26.9 mg L^−1^ d^−1^) by day 4, at the lowest temperature and highest N:P ratio. This confirms the consensus that proteins are typically prioritized at near-optimal conditions for growth. It was also anticipated that excess nitrogen would result in higher protein production, since it is a crucial nutrient for these macromolecules. Total FA and TC productivities exhibit inverse relationships with temperature (maximum values of 26.4 mg L^−1^ d^−1^ and 16.0 mg L^−1^ d^−1^, respectively), which can be attributed to the impact of the steep changes in biomass concentrations masking the trends of composition changes.

Finally, a brief analysis of the FA, the carotenoid, and the amino acid profiles was conducted. In all of them, increased variability was apparent along the cultivation period, although the specific influence of temperature and N:P was not dissected in the present work. The fact that PUFAs had the largest share of the FA profile—and, among them, that two essential fatty acids (ALA and LA) shared the most significant portion—should be outlined. Lutein was undoubtedly dominant among the analyzed carotenoids; among the amino acids, several of them were essential nutrients, and ARG stood out by having an up to 25% section of the profile, depending on the cultivation conditions.

## Figures and Tables

**Table 1:** Results of the experimental planning using central composite design, varying temperature, and N:P ratio as independent factors. The variables *Z*_1_ and *Z*_2_ were transformed into levels x_1_ and x_2_.

		Temperature		N:P Ratio		
Experiment	Positioning in CCD	Level (*x*_1_)	Value (*Z*_1_)	Level (*x*_2_)	log_3_ (N:P) (*Z*_2_)	Value
E1	Central (1st replica)	0	25.0 °C	0	2.00	9.00
E2	Central (2nd replica)	0	25.0 °C	0	2.00	9.00
E3	Axial (right)	+α	32.0 °C	0	2.00	9.00
E4	Axial (left)	−α	18.0 °C	0	2.00	9.00
E5	Factorial (bottom left)	−1	20.0 °C	−1	1.00	3.00
E6	Factorial (bottom right)	+1	30.0 °C	−1	1.00	3.00
E7	Factorial (upper left)	−1	20.0 °C	+1	3.00	27.0
E8	Factorial (upper right)	+1	30.0 °C	+1	3.00	27.0
E9	Axial (bottom)	0	25.0 °C	−α	0.59	1.90
E10	Axial (upper)	0	25.0 °C	+α	3.41	42.6
E11	Central (3rd replica)	0	25.0 °C	0	2.00	9.00
E12	Central (4th replica)	0	25.0 °C	0	2.00	9.00

**Table 2:** Quantitative parameters regarding growth and nutrient consumption for the 14-day cultivation of *C. vulgaris* under the conditions defined by experimental runs 1 through 12. *µ*: microalgal growth rate; *P_X,Avg_*: average biomass productivity; *RE*: removal efficiency; *RR*: average removal rate.

Growth and Biomass Productivity					Nutrient Consumption			
	*µ* (d^−1^)	*P_X,Avg_* (mg_dw_ L^−1^ d^−1^)			NO_3_-N|Nitrogen		PO_4_-P|Phosphorus	
		*t*_0_ → *t*_4_	*t*_0_ → *t*_7_	*t*_0_ → *t*_14_	*RE* (%)	*RR* (mg L^−1^ d^−1^)	*RE *(%)	*RR* (mg L^−1^ d^−1^)
E1	0.296 ± 0.004	99.0 ± 1.0	87.6 ± 0.5	54.7 ± 0.3	97.9 ± 0.2	3.17 ± 0.04	100.0 ± 0.1	0.76 ± 0.05
E2	0.334 ± 0.001	107.2 ± 0.3	85.4 ± 0.3	52.5 ± 0.2	96.9 ± 0.3	3.14 ± 0.05	99.9 ± 0.1	0.76 ± 0.05
E3	0.145 ± 0.002	46.0 ± 2.0	43.0 ± 1.0	31.2 ± 0.3	58.0 ± 1.0	1.94 ± 0.09	48.0 ± 2.0	0.37 ± 0.03
E4	0.281 ± 0.005	119.0 ± 3.0	94.0 ± 4.0	68.0 ± 2.0	96.1 ± 0.1	2.87 ± 0.01	100.1 ± 0.1	0.67 ± 0.02
E5	0.350 ± 0.001	117.7 ± 0.9	84.0 ± 0.3	58.0 ± 2.0	90.0 ± 2.0	0.96 ± 0.04	81.1 ± 0.4	0.58 ± 0.01
E6	0.267 ± 0.003	77.0 ± 1.0	65.3 ± 0.5	45.5 ± 0.8	83.6 ± 0.2	0.91 ± 0.02	72.0 ± 1.0	0.51 ± 0.01
E7	0.423 ± 0.001	122.3 ± 0.4	88.0 ± 2.0	64.0 ± 1.0	54.0 ± 2.0	4.70 ± 0.20	99.7 ± 0.1	0.64 ± 0.01
E8	0.254 ± 0.001	72.4 ± 0.2	70.9 ± 0.4	41.4 ± 0.4	28.0 ± 2.0	2.80 ± 0.20	96.8 ± 0.1	0.61 ± 0.01
E9	0.250 ± 0.040	93.6 ± 0.5	69.0 ± 2.0	47.1 ± 0.2	90.9 ± 0.2	0.67 ± 0.01	73.5 ± 0.4	0.50 ± 0.01
E10	0.341 ± 0.001	100.3 ± 1.0	83.4 ± 0.2	46.2 ± 0.6	28.0 ± 1.0	4.40 ± 0.30	98.4 ± 0.2	0.66 ± 0.05
E11	0.317 ± 0.001	110.9 ± 0.4	92.0 ± 1.0	59.0 ± 3.0	97.7 ± 0.1	2.90 ± 0.04	100.2 ± 0.1	0.64 ± 0.02
E12	0.266 ± 0.002	93.0 ± 1.0	75.0 ± 1.0	43.0 ± 1.0	97.1 ± 0.1	3.03 ± 0.08	100.1 ± 0.2	0.62 ± 0.04

**Table 3:** Parameters that evaluate the model for average biomass productivity and point of response optimization. Obtained with Minitab software (Version 22.3.1.0). RSME: root mean square error; $R^{2}$: coefficient of determination; $R_{Adj}^{2}$: adjusted $R^{2}$; $R_{Pred}^{2}$: predicted $R^{2}$.

Model Summary				Response Optimization		
RMSE	$R^{2}$	$R_{Adj}^{2}$	$R_{Pred}^{2}$	y-Value Fit (mg_dw_ L^−1^ d^−1^)	Setting	
7.0	0.93	0.92	0.89	122.27	$x_{1}$$\;=\;-\sqrt{2}$ ^†^	t = 4

^†^ Optimum lies on the boundary of the design region.

**Table 4:** Compilation of parameters used to evaluate the models and points of response optimization.

		Model Summary				Response Optimization	
		RMSE	$R^{2}$	$R_{Adj}^{2}$	$R_{Pred}^{2}$	y Fit	Setting
TP	Content RT	0.16	0.61	0.54	0.45	0.45	$\left(x_{1},x_{2},t\right)=\left(-0.014,0.30,4\right)$
	Concentration RT	0.91	0.78	0.74	0.69	4.2	$\left(x_{1},x_{2},t\right)=\left(-0.443,0.414,14\right)$
	$P_{Avg}$ (mg L^−1^ d^−1^)	3.5	0.76	0.71	0.64	27	$\left(x_{1},x_{2},t\right)=(-\sqrt{2}$ ^†^$,\sqrt{2}$ ^†^,4)
Total FA	Content RT	0.25	0.86	0.83	0.73	1.54	$\left(x_{1},t\right)=\left(-\sqrt{2},14\right)$
	Concentration RT	1.6	0.92	0.89	0.82	14	$\left(x_{1},x_{2},t\right)=(-\sqrt{2}$ ^†^,0.043,14)
	$P_{Avg}$ (mg L^−1^ d^−1^)	4.5	0.68	0.66	0.58	26	$x_{1}=-\sqrt{2}$ ^†^
TC	Content RT	0.20	0.61	0.54	0.40	0.82	$(x_{2},t)=(-\sqrt{2}$ ^†^,14)
	Concentration RT	1.3	0.79	0.75	0.68	6.2	$\left(x_{1},x_{2},t\right)=\left(-0.614,-0168,14\right)$
	$P_{Avg}$ (mg L^−1^ d^−1^)	2.3	0.70	0.67	0.62	16	$(x_{1},t)=(-\sqrt{2}$ ^†^,4)
Total pigments	$P_{Avg}$ (mg L^−1^ d^−1^)	0.15	0.95	0.94	0.93	2.4	$(x_{1},t)=(-\sqrt{2}$ ^†^,4)
Chlorophyll a		0.083	0.94	0.93	0.92	1.3	$(x_{1},t)=(-\sqrt{2}$ ^†^,4)
Chlorophyll b		0.044	0.93	0.92	0.91	0.60	$(x_{1},t)=(-\sqrt{2}$ ^†^,4)
Carotenoids		0.043	0.92	0.90	0.86	0.50	$(x_{1},t)=(-\sqrt{2}$ ^†^,4)

^†^ Optimum lies on the boundary of the design region. TP—Total Proteins; Total FA—Total Fatty Acids; TC—Total Carbohydrates.

**Table 5:** Compilation of *β* regression coefficients considered significant (p≤0.1) from all models, after backward elimination with hierarchy enforced, in coded units for better comparison of factor effects. Results presented in the form: *β* estimate (*p*-value).

			Factors					
Models			Independent	$x_{1}$ (T Level)	$x_{2}$ (N:P Level)	$x_{1}^{2}$	$x_{2}^{2}$	$x_{1}x_{2}$
$P_{X,avg}$ (mg_dw_ L^−1^ d^−1^)		*t*_4_	99.7 (≤0.1)	−24.2 (≤0.1)	n.s.	−4.78 (0.01)	n.s.	n.s.
		*t*_7_	81.3 (≤0.1)	−13.5 (≤0.1)				
		*t*_14_	54.1 (≤0.1)	−10.9 (≤0.1)				
TP	Content *RT*	*t*_4_	0.445 (≤0.001)	−0.00310 (0.91100) *	0.0645 (0.0250)	−0.0838 (0.0100)	−0.112 (0.001)	
		*t*_7_	0.215 (≤0.001)					
		*t*_14_	0.0665 (≤0.0001)					
	Concentration *RT*	*t*_4_	3.53 (≤0.01)	−0.464 (0.096)	0.407 (0.015)	−0.511 (0.007)	−0.493 (0.009)	
		*t*_7_	3.85 (≤0.01)					
		*t*_14_	4.94 (≤0.01)					
	$P_{Avg}$ (mg L^−1^ d^−1^)	*t*_4_	17.9 (≤0.1)	−5.62 (≤0.01)	1.42 (0.06)	n.s.	n.s.	
		*t*_7_	10.9 (≤0.1)	−2.50 (≤0.01)				
		*t*_14_	5.75 (≤0.01)	−1.43 (≤0.01)				
Total FA	Content *RT*	*t*_7_	0.539 (≤0.001)	−0.234 (0.002)	n.s.	−0.121 (0.078)	n.s.	
		*t*_14_	1.31 (≤0.01)	−0.339 (0.002)				
	Concentration *RT*	*t*_7_	5.21 (≤0.01)	−2.39 (0.001)	0.0890 (0.7940) *	n.s.	−0.812 (0.072)	
		*t*_14_	10.9 (≤0.01)	−2.11 (0.001)				
	$P_{Avg}$ (mg L^−1^ d^−1^)	*t*_7_	16.0 (≤0.001)	−7.36 (≤0.01)	n.s.	n.s.	n.s.	
		*t*_14_						
TC	Content *RT*	*t*_4_	0.367 (≤0.001)	n.s.	−0.202 (≤0.001)	n.s.	n.s.	
		*t*_7_	0.434 (≤0.001)		−0.136 (≤0.001)			
		*t*_14_	0.398 (≤0.001)		−0.298 (≤0.001)			
	Concentration *RT*	*t*_4_	3.63 (≤0.01)	−0.629 (0.008)	−0.238 (0.296) *	−0.518 (0.043)	−0.623 (0.016)	
		*t*_7_	5.48 (≤0.01)					
		*t*_14_	5.94 (≤0.01)					
	$P_{Avg}$ (mg L^−1^ d^−1^)	*t*_4_	13.0 (≤0.1)	−2.16 (≤0.01)	n.s.	n.s.	n.s.	
		*t*_7_	10.8 (≤0.1)					
		*t*_14_	6.36 (≤0.01)					
Total pigments	*P_AVG_* (mg L^−1^ d^−1^)	*t*_4_	1.23 (≤0.01)	−0.838 (≤0.001)	n.s.	n.s.	n.s.	
		*t*_7_	0.380 (≤0.001)	−0.217 (≤0.001)				
		*t*_14_	0.0272 (≤0.0001)	−0.0224 (≤0.0001)				
Chlorophyll a		*t*_4_	0.616 (≤0.001)	−0.456 (≤0.001)	n.s.	n.s.	n.s.	
		*t*_7_	0.129 (≤0.001)	−0.102 (≤0.001)				
		*t*_14_	−0.0092 (≤0.0001)	−0.0023 (≤0.0001)				
Chlorophyll b		*t*_4_	0.314 (≤0.001)	−0.205 (≤0.001)	n.s.	n.s.	n.s.	
		*t*_7_	0.0907 (≤0.0001)	−0.0649 (≤0.0001)				
		*t*_14_	0.0084 (≤0.0001)	−0.0102 (≤0.0001)				
Carotenoids		*t*_4_	0.308 (≤0.001)	−0.167 (≤0.001)	n.s.	−0.0230 (0.060)	n.s.	
		*t*_7_	0.193 (≤0.001)	−0.0782 (≤0.0001)				
		*t*_14_	0.0435 (≤0.0001)	−0.0100 (≤0.0001)				

* Coefficients that were not removed despite p>0.1, according to the principle of marginality. n.s.: non-significant factor (p>0.1). TP—Total Proteins; Total FA—Total Fatty Acids; TC—Total Carbohydrates.

## Data Availability

All data generated or analysed during this study are included in this published article and its Supplementary Information.

## Supplementary Materials

The following supporting information can be downloaded at: https://www.mdpi.com/article/10.3390/md24040124/s1. Figure S1: Pictures of the experimental setup used for microalgal cultivation. (a) One of the fermenters of the Fermentec FMT DSA-D series, with a *C. vulgaris* suspension and a LED string setup; (b) user interface of the program Fermentec HMI System; (c) full view of the fermenter and monitor; Figure S2: Calibration curve of OD versus biomass concentration (mg L^−1^), displaying the equation of the fitted regression line and the coefficient of determination (*R*^2^). LOD: limit of detection; LOQ: limit of quantification; Figure S3: Calibration curves of absorbance (220 nm) versus nitrate concentration (mg L^−1^) and absorbance (820 nm) versus phosphate concentration (mg L^−1^), both displaying the equation of fitted regression line and the coefficient of determination (*R*^2^). LOD: limit of detection; LOQ: limit of quantification; Figure S4: Calibration curves of absorbance (500 nm and 750 nm, for low and high concentration ranges, respectively) versus BSA concentration (µg mL^−1^) and absorbance (490 nm) versus glucose concentration (mg L^−1^), both displaying the equation of fitted regression line and the coefficient of determination (*R*^2^). LOD: limit of detection; LOQ: limit of quantification; Figure S5: Calibration curves for carotenoids, of absorbance (450 nm) versus carotenoid concentration (mg mL^−1^), all displaying the equation of fitted regression line and the coefficient of determination (*R*^2^). LOD: limit of detection; LOQ: limit of quantification; Figure S6: Calibration curve of peak area ratio versus concentration of each studied amino acid (µM), displaying the equation of fitted regression line and the coefficient of determination (*R*^2^). LOD: limit of detection; LOQ: limit of quantification; Figure S7: Curves of biomass concentration along time for the 14-day cultivation of *C. vulgaris* under the conditions defined by experimental runs 1 through 12. The biological replicas of the central point (E1, E2, E11 and E12) are represented by their average, surrounded by a shaded area defined by their standard deviations; Table S1: Biomass concentration values, *X* (mg_dw_ L^−1^), at days 0, 4, 7 and 14, for the 12 points of the experimental design, as well as input levels and uncoded values of temperature and N:P ratio; Table S2: Total protein content (% *w*/*w*), at days 0, 4, 7 and 14, and total protein average productivity, *P_Avg_* (mg L^−1^ d^−1^) at the end of days 4, 7 and 14, for the 12 points of the experimental design; Table S3: Total fatty acids content (% *w*/*w*) at days 0, 4, 7 and 14, and total fatty acids average productivity, *P_Avg_* (mg L^−1^ d^−1^) at the end of days 4, 7 and 14, for the 12 points of the experimental design; Table S4: Total carbohydrate content (% *w*/*w*) at days 0, 4, 7 and 14, and total carbohydrate average productivity, *P_Avg_* (mg L^−1^ d^−1^) at the end of days 4, 7 and 14, for the 12 points of the experimental design; Table S5: Total pigment content (% *w*/*w*) at days 0, 4, 7 and 14, and total pigment average productivity, *P_Avg_* (mg L^−1^ d^−1^) at the end of days 4, 7 and 14; Table S6: Chlorophyll a content (% *w*/*w*) at days 0, 4, 7 and 14, and chlorophyll a average productivity, *P_Avg_* (mg L^−1^ d^−1^) at the end of days 4, 7 and 14; Table S7: Chlorophyll b content (% *w*/*w*) at days 0, 4, 7 and 14, and chlorophyll b average productivity, *P_Avg_* (mg L^−1^ d^−1^) at the end of days 4, 7 and 14; Table S8: Total carotenoid content (% *w*/*w*) at days 0, 4, 7 and 14, and total carotenoid average productivity, *P_Avg_* (mg L^−1^ d^−1^) at the end of days 4, 7 and 14; Table S9: C16:0 (Palmitic acid) content (% *w*/*w*) at days 0, 7 and 14, and C16:0 average productivity, *P_Avg_* (mg L^−1^ d^−1^), at the end of days 7 and 14; Table S10: C18:0 (Steraic acid) content (% *w*/*w*) at days 0, 7 and 14, and C18:0 average productivity, *P_Avg_* (mg L^−1^ d^−1^), at the end of days 7 and 14; Table S11: C16:1n9 (Palmitoleic acid) content (% *w*/*w*) at days 0, 7 and 14, and C16:1n9 average productivity, *P_Avg_* (mg L^−1^ d^−1^), at the end of days 7 and 14; Table S12: C16:1n7 (Palmitoleic acid) content (% *w*/*w*) at days 0, 7 and 14, and C16:1n7 average productivity, *P_Avg_* (mg L^−1^ d^−1^), at the end of days 7 and 14; Table S13: C18:1n9c (Oleic acid) content (% *w*/*w*) at days 0, 7 and 14, and C18:1n9c average productivity, *P_Avg_* (mg L^−1^ d^−1^), at the end of days 7 and 14; Table S14: C18:1n7c content (% *w*/*w*) at days 0, 7 and 14, and C18:1n7c average productivity, *P_Avg_* (mg L^−1^ d^−1^), at the end of days 7 and 14; Table S15: C16:2 content (% *w*/*w*) at days 0, 7 and 14, and C16:2 average productivity, *P_Avg_* (mg L^−1^ d^−1^), at the end of days 7 and 14; Table S16: C16:3 content (% *w*/*w*) at days 0, 7 and 14, and C16:3 average productivity, *P_Avg_* (mg L^−1^ d^−1^), at the end of days 7 and 14; Table S17: C16:4 content (% *w*/*w*) at days 0, 7 and 14, and C16:4 average productivity, *P_Avg_* (mg L^−1^ d^−1^), at the end of days 7 and 14; Table S18: C18:2cc (Linoleic acid) content (% *w*/*w*) at days 0, 7 and 14, and C18:2cc average productivity, *P_Avg_* (mg L^−1^ d^−1^), at the end of days 7 and 14; Table S19: C18:3n3 (α-linoleic acid) content (% *w*/*w*) at days 0, 7 and 14, and C18:3n3 average productivity, *P_Avg_* (mg L^−1^ d^−1^), at the end of days 7 and 14; Table S20: Neoxanthin content (mg g_dw_^−1^) at days 0, 7 and 14, and neoxanthin average productivity, *P_Avg_* (mg L^−1^ d^−1^), at the end of days 7 and 14; Table S21: Violaxanthin content (mg g_dw_^−1^) at days 0, 7 and 14, and violaxanthin average productivity, *P_Avg_* (mg L^−1^ d^−1^), at the end of days 7 and 14; Table S22: Lutein content (mg g_dw_^−1^) at days 0, 7 and 14, and lutein average productivity, *P_Avg_* (mg L^−1^ d^−1^), at the end of days 7 and 14; Table S23: Zeaxanthin content (mg g_dw_^−1^) at days 0, 7 and 14, and zeaxanthin average productivity, *P_Avg_* (mg L^−1^ d^−1^), at the end of days 7 and 14; Table S24: β-carotene content (mg g_dw_^−1^) at days 0, 7 and 14, and β-carotene average productivity, *P_Avg_* (mg L^−1^ d^−1^), at the end of days 7 and 14; Table S25: ALA content (mg g_dw_^−^^1^), at days 0, 4, 7 and 14, and ALA average productivity, *P_Avg_* (mg L^−1^ d^−1^), at the end of days 4, 7 and 14; Table S26: ARG content (mg g_dw_^−^^1^), at days 0, 4, 7 and 14, and ARG average productivity, *P_Avg_* (mg L^−1^ d^−1^), at the end of days 4, 7 and 14; Table S27: ASP content (mg g_dw_^−^^1^), at days 0, 4, 7 and 14, and ASP average productivity, *P_Avg_* (mg L^−1^ d^−1^), at the end of days 4, 7 and 14; Table S28: GLU content (mg g_dw_^−^^1^), at days 0, 4, 7 and 14, and GLU average productivity, *P_Avg_* (mg L^−1^ d^−1^), at the end of days 4, 7 and 14; Table S29: GLY content (mg g_dw_^−^^1^), at days 0, 4, 7 and 14, and GLY average productivity, *P_Avg_* (mg L^−1^ d^−1^), at the end of days 4, 7 and 14; Table S30: HIS content (mg g_dw_^−^^1^), at days 0, 4, 7 and 14, and HIS average productivity, *P_Avg_* (mg L^−1^ d^−1^), at the end of days 4, 7 and 14; Table S31: ILE content (mg g_dw_^−^^1^), at days 0, 4, 7 and 14, and ILE average productivity, *P_Avg_* (mg L^−1^ d^−1^), at the end of days 4, 7 and 14; Table S32: LEU content (mg g_dw_^−^^1^), at days 0, 4, 7 and 14, and LEU average productivity, *P_Avg_* (mg L^−1^ d^−1^), at the end of days 4, 7 and 14; Table S33: LYS content (mg g_dw_^−^^1^), at days 0, 4, 7 and 14, and LYS average productivity, *P_Avg_* (mg L^−1^ d^−1^), at the end of days 4, 7 and 14; Table S34: MET content (mg g_dw_^−^^1^), at days 0, 4, 7 and 14, and MET average productivity, *P_Avg_* (mg L^−1^ d^−1^), at the end of days 4, 7 and 14; Table S35: PHE content (mg g_dw_^−^^1^), at days 0, 4, 7 and 14, and PHE average productivity, *P_Avg_* (mg L^−1^ d^−1^), at the end of days 4, 7 and 14; Table S36: PRO content (mg g_dw_^−^^1^), at days 0, 4, 7 and 14, and PRO average productivity, *P_Avg_* (mg L^−1^ d^−1^), at the end of days 4, 7 and 14; Table S37: SER content (mg g_dw_^−^^1^), at days 0, 4, 7 and 14, and SER average productivity, *P_Avg_* (mg L^−1^ d^−1^), at the end of days 4, 7 and 14; Table S38: THR content (mg g_dw_^−^^1^), at days 0, 4, 7 and 14, and THR average productivity, *P_Avg_* (mg L^−1^ d^−1^), at the end of days 4, 7 and 14; Table S39: TYR content (mg g_dw_^−^^1^), at days 0, 4, 7 and 14, and TYR average productivity, *P_Avg_* (mg L^−1^ d^−1^), at the end of days 4, 7 and 14; Table S40: VAL content (mg g_dw_^−1^), at days 0, 4, 7 and 14, and VAL average productivity, *P_Avg_* (mg L^−1^ d^−1^), at the end of days 4, 7 and 14.

## Abbreviations and Nomenclature:

The following abbreviations are used in this manuscript:

ALA	Alpha-linoleic acid	
ANN	Artificial Neural Network	
ANOVA	Analysis of Variance	
ATP	Adenosine Triphosphate	
CAGR	Compound Annual Growth Rate	
CCAP	Culture Collection of Algae and Protozoa	
CCD	Central Composite Design	
DNA	Deoxyribonucleic acid	
DoE	Design of Experiments	
EAA	Essential Amino Acid	
EPA	Eicosapentaenoic acid	
FA	Fatty Acid	
FAME	Fatty Acid Methyl Ester	
GC	Gas Chromatography	
GM	Growth Medium	
HPLC	High-Performance Liquid Chromatography	
HSD	Honestly Significant Difference	
LA	Linoleic acid	
LOD	Limit of Detection	
LOQ	Limit of Quantification	
MUFA	Monounsaturated Fatty Acid	
OD	Optical Density	
OECD	Organization for Economic Cooperation and Development	
PUFA	Polyunsaturated Fatty Acid	
RMSE	Root Mean Square Error	
RNA	Ribonucleic acid	
ROS	Reactive Oxygen Species	
RSM	Response Surface Methodology	
SD	Standard Deviation	
SDG	Sustainable Development Goals	
SE	Standard Error	
SFA	Saturated Fatty Acid	
T	Temperature	
TAG	Triacylglycerol	
TC	Total Carbohydrates	
TP	Total Proteins	
UFA	Unsaturated Fatty Acid	
UN	United Nations	
Symbols		
*x*_1_	Independent variable 1 (level of temperature)	
*x*_2_	Independent variable 2 (level of N:P molar ratio)	
*Z*_1_	Variable representing temperature	°C
*Z*_2_	Variable representing N:P molar ratio	
β	Regression coefficient	
y	Response variable	
*α*	CCD parameter	
E(X)	Experiment number X	
*R*^2^	Coefficient of determination	%
*X*	Biomass concentration	mg_dw_ L^−1^
*μ*	Growth rate	d^−1^
*t*	Time	d
*P*	Productivity	mg L^−1^ d^−1^
*RE*	Removal Efficiency	%
*RR*	Average Removal Rate	mg L^−1^ d^−1^
*S*	Substrate/Nutrient	mg L^−1^
*λ*	Wavelength	nm
*RT*	Relative Tendency	
Indexes		
*i*; *j*	Variable counter	
0	Initial	
*f*	Final	
*exp*	Exponential	
*dw*	Dry weight	
*X*	Biomass	
*Avg*	Average	
*Adj*	Adjusted	
*Pred*	Predicted	
