# Supplementary material for: On the Design of Chlorella vulgaris Composition for Potential Food Uses via Manipulation of Cultivation Conditions
Source: Mar Drugs. 2026 Mar 26;24(4):124. doi: 10.3390/md24040124 (PMC13117707; doi:10.3390/md24040124)
Supplement: Supplementary file 1 [file marinedrugs-24-00124-s001.zip › marinedrugs-4168100-supplementary.pdf]

Supplementary Material of

On the design of *Chlorella vulgaris* composition for potential food uses via manipulation of cultivation conditions

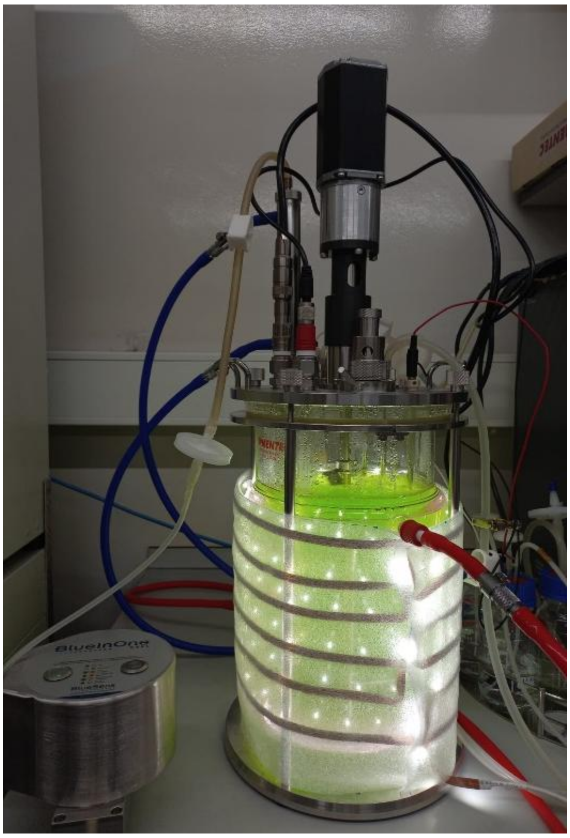

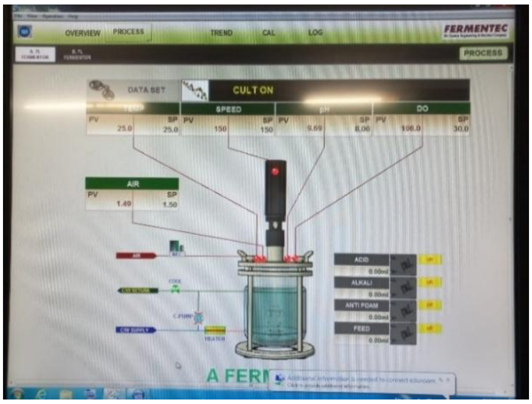

(b)

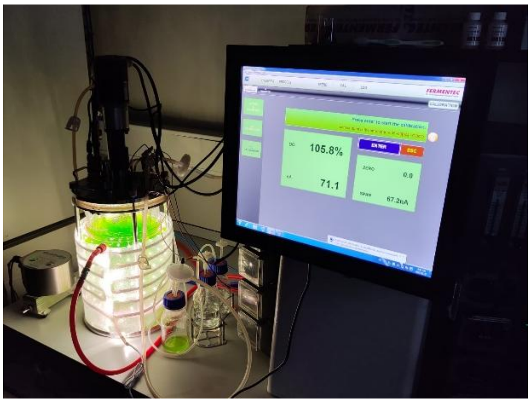

(a) (c)

Figure S1: Pictures of the experimental setup used for microalgal cultivation. (**a**) One of the fermenters of the Fermentec FMT DSA-D series, with a *C. vulgaris* suspension and a LED string setup; (**b**) user interface of the program Fermentec HMI System; (**c**) full view of the fermenter and monitor.

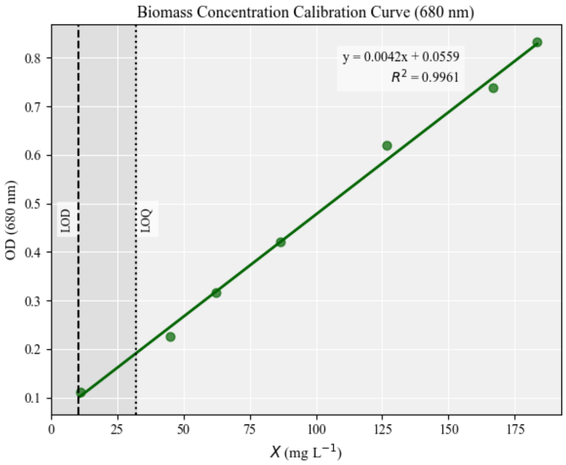

Figure S2: Calibration curve of OD versus biomass concentration (mg L^{-1}), displaying the equation of the fitted regression line and the coefficient of determination (R^2). LOD: limit of detection; LOQ: limit of quantification.

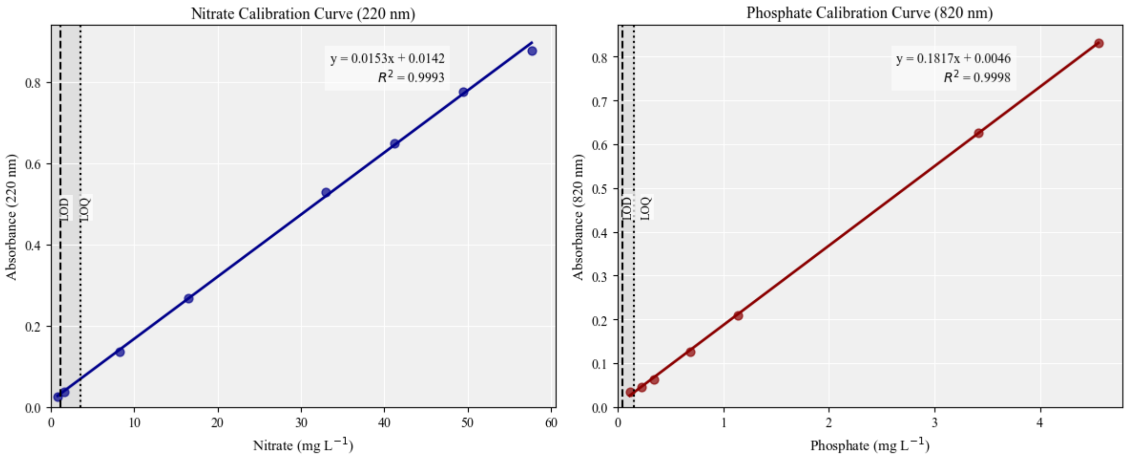

Figure S3: Calibration curves of absorbance (220 nm) versus nitrate concentration (mg L^{-1}) and absorbance (820 nm) versus phosphate concentration (mg L^{-1}), both displaying the equation of fitted regression line and the coefficient of determination (R^2). LOD: limit of detection; LOQ: limit of quantification.

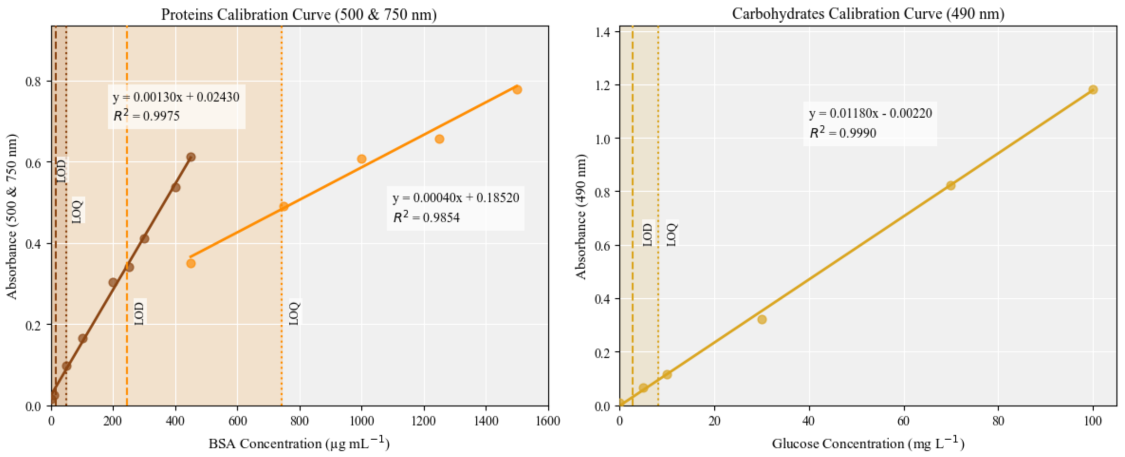

Figure S4: Calibration curves of absorbance (500 nm and 750 nm, for low and high concentration ranges, respectively) versus BSA concentration (µg mL^{-1}) and absorbance (490 nm) versus glucose concentration (mg L^{-1}), both displaying the equation of fitted regression line and the coefficient of determination (R^2). LOD: limit of detection; LOQ: limit of quantification.

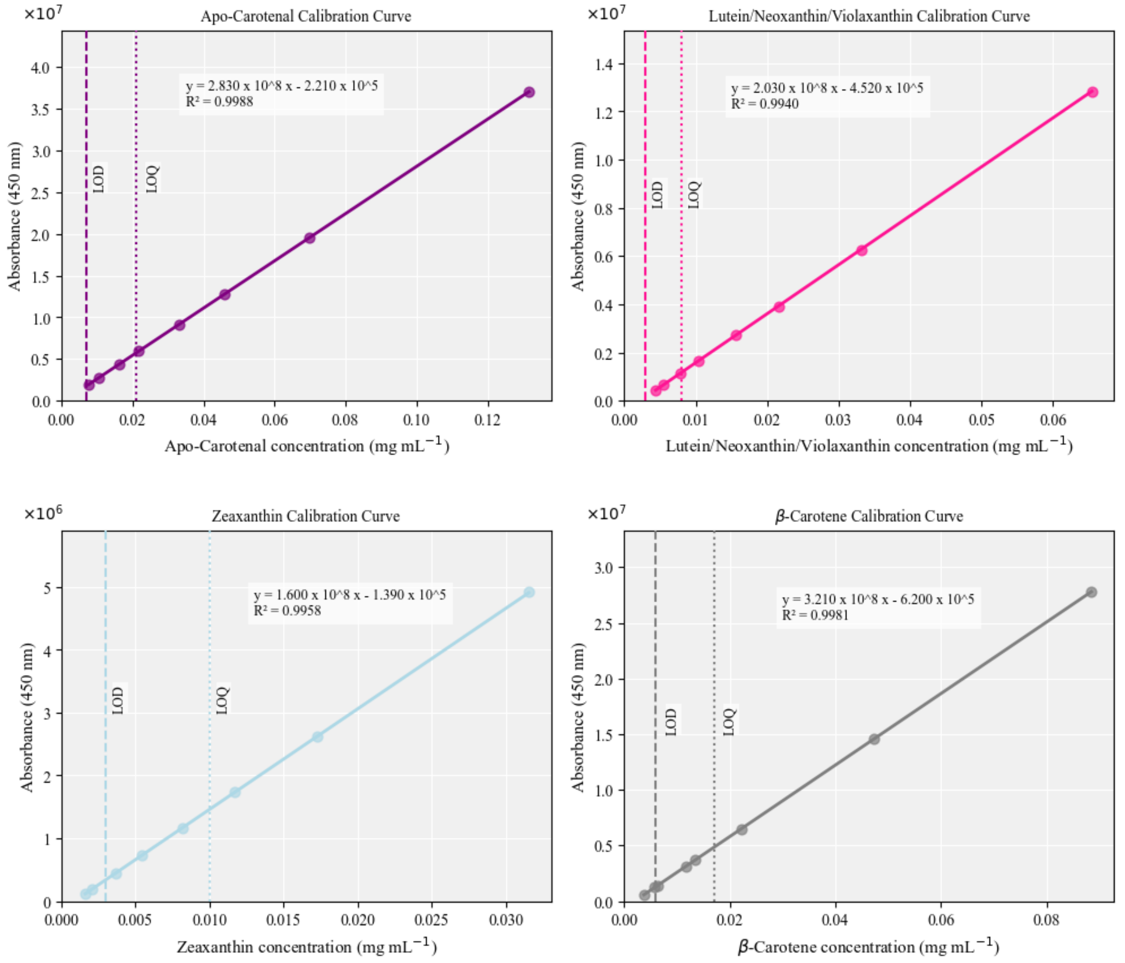

Figure S5: Calibration curves for carotenoids, of absorbance (450 nm) versus carotenoid concentration (mg mL^{-1}), all displaying the equation of fitted regression line and the coefficient of determination (R^2). LOD: limit of detection; LOQ: limit of quantification.

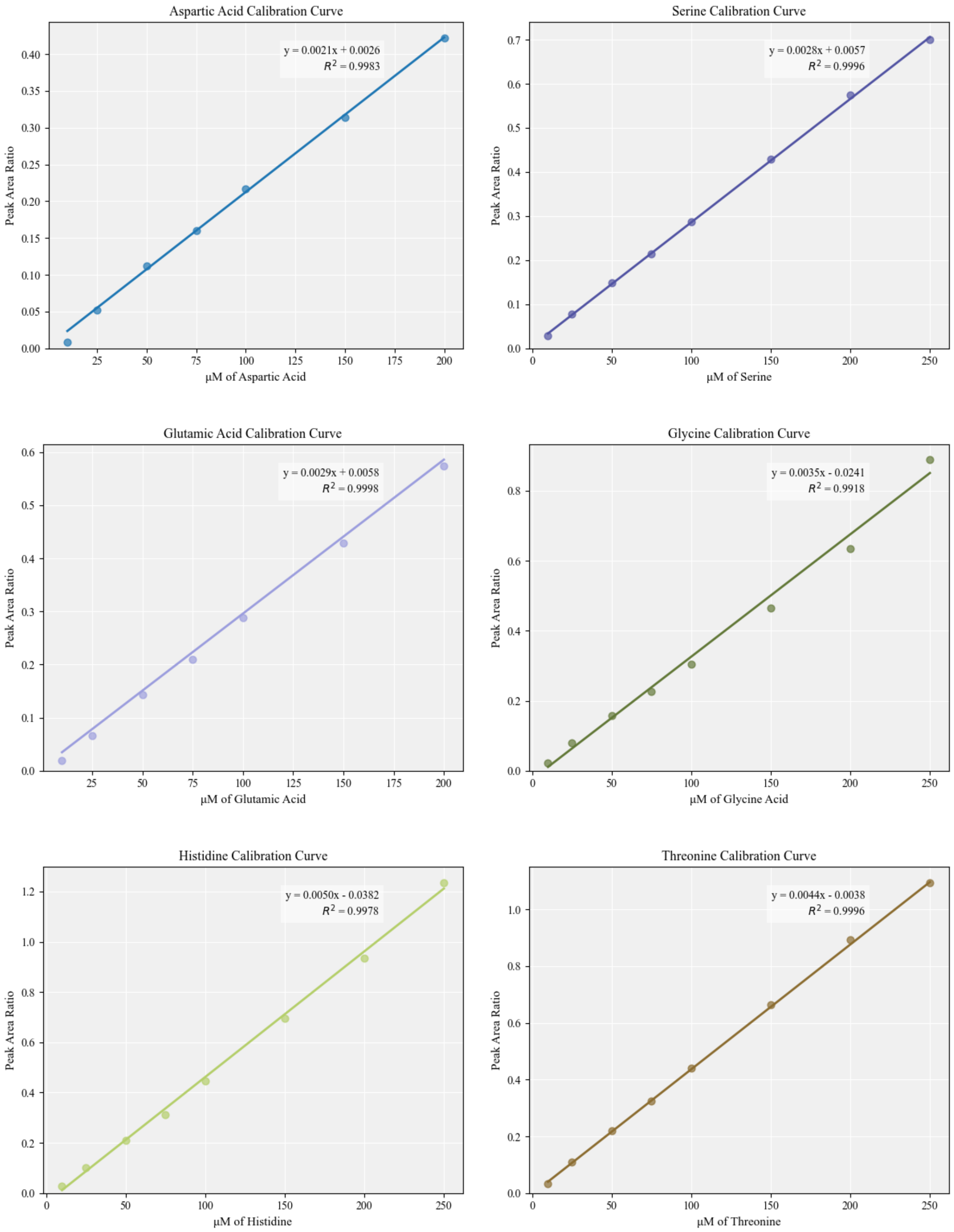
Aspartic Acid Calibration Curve
y = 0.0021x + 0.0026
R^2 = 0.9983
Peak Area Ratio
µM of Aspartic Acid
Serine Calibration Curve
y = 0.0028x + 0.0057
R^2 = 0.9996
Peak Area Ratio
µM of Serine
Glutamic Acid Calibration Curve
y = 0.0029x + 0.0058
R^2 = 0.9998
Peak Area Ratio
µM of Glutamic Acid
Glycine Calibration Curve
y = 0.0035x - 0.0241
R^2 = 0.9918
Peak Area Ratio
µM of Glycine Acid
Histidine Calibration Curve
y = 0.0050x - 0.0382
R^2 = 0.9978
Peak Area Ratio
µM of Histidine
Threonine Calibration Curve
y = 0.0044x - 0.0038
R^2 = 0.9996
Peak Area Ratio
µM of Threonine

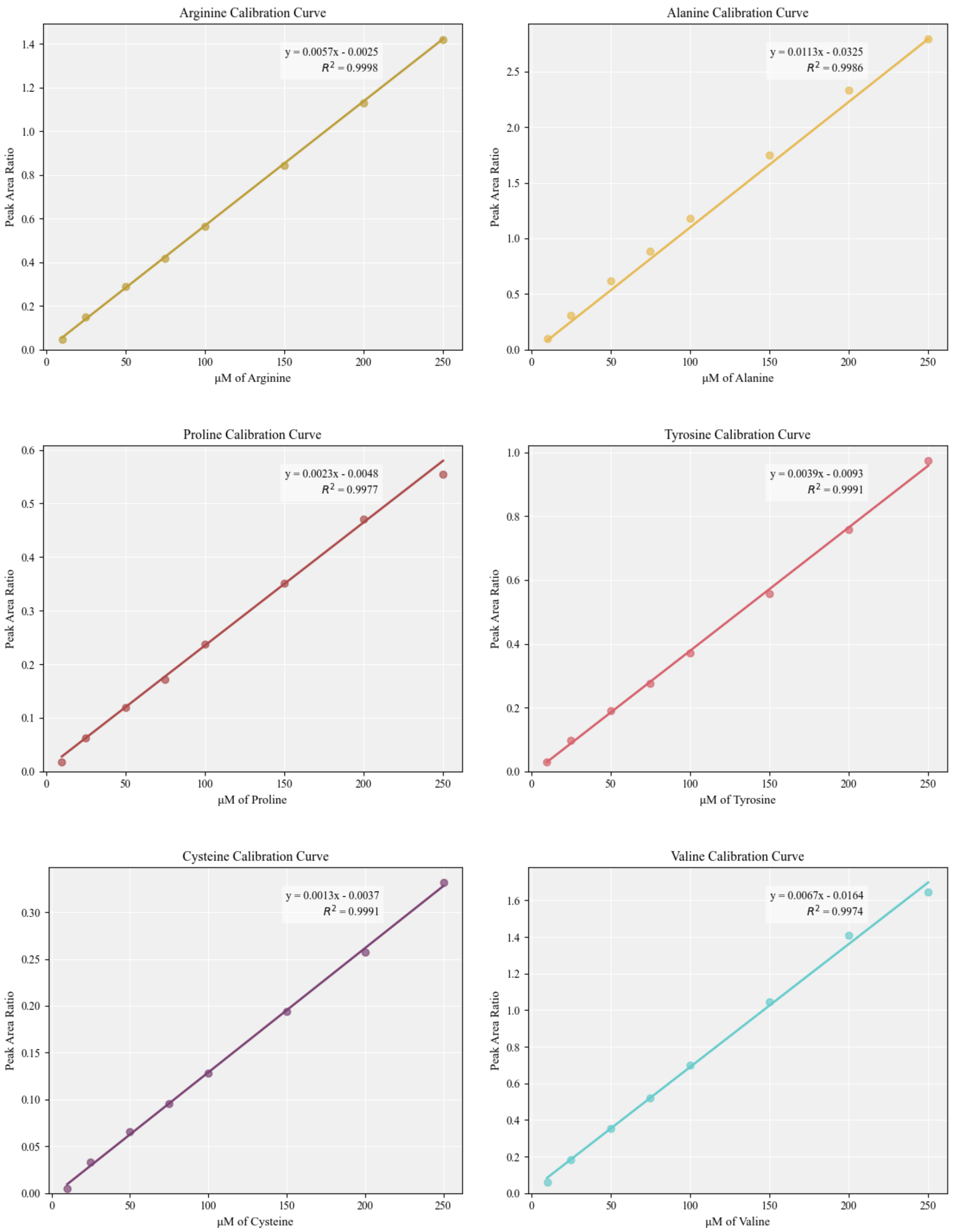
Arginine Calibration Curve
y = 0.0057x - 0.0025
R^2 = 0.9998
Peak Area Ratio
µM of Arginine
Alanine Calibration Curve
y = 0.0113x - 0.0325
R^2 = 0.9986
Peak Area Ratio
µM of Alanine
Proline Calibration Curve
y = 0.0023x - 0.0048
R^2 = 0.9977
Peak Area Ratio
µM of Proline
Tyrosine Calibration Curve
y = 0.0039x - 0.0093
R^2 = 0.9991
Peak Area Ratio
µM of Tyrosine
Cysteine Calibration Curve
y = 0.0013x - 0.0037
R^2 = 0.9991
Peak Area Ratio
µM of Cysteine
Valine Calibration Curve
y = 0.0067x - 0.0164
R^2 = 0.9974
Peak Area Ratio
µM of Valine

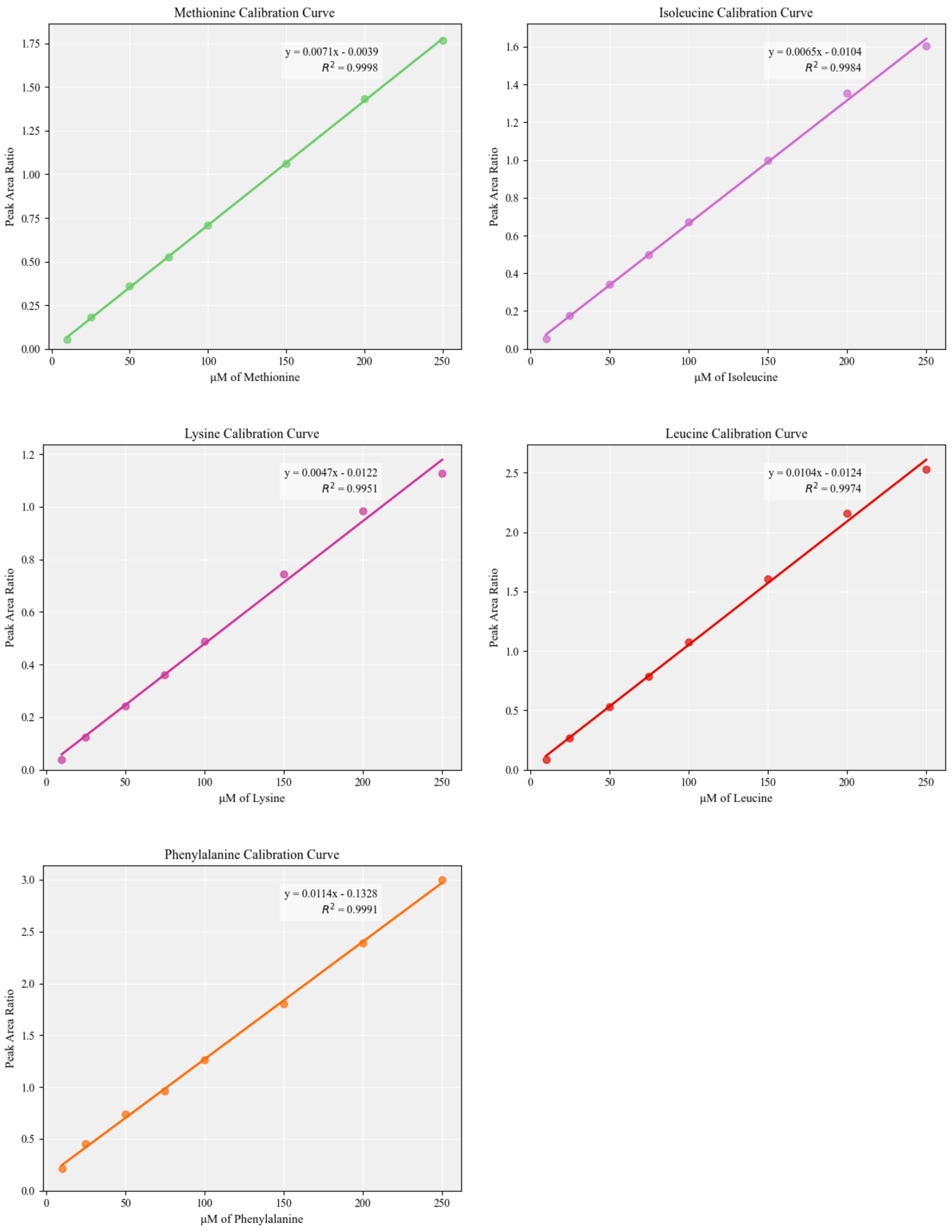

Figure S6: Calibration curve of peak area ratio versus concentration of each studied amino acid (µM), displaying the equation of fitted regression line and the coefficient of determination (R^2). LOD: limit of detection; LOQ: limit of quantification.

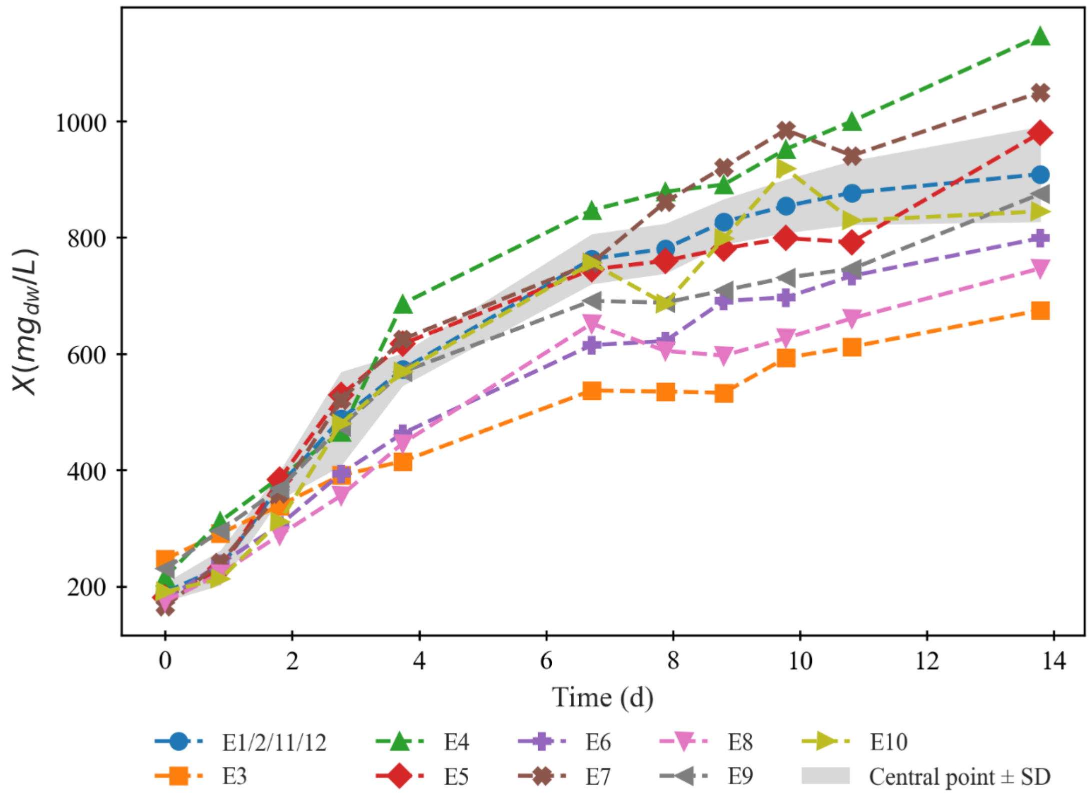

Figure S7: Curves of biomass concentration along time for the 14-d cultivation of *C. vulgaris* under the conditions defined by experimental runs 1 through 12. The biological replicas of the central point (E1, E2, E11 and E12) are represented by their average, surrounded by a shaded area defined by their standard deviations.

Table S1: Biomass concentration values, X (mg_{dw} L^{-1}), at days 0, 4, 7 and 14, for the 12 points of the experimental design, as well as input levels and uncoded values of temperature and N:P ratio.

	Temperature		N:P ratio		X (mg_{dw} L^{-1})			
	Level (x_1)	Value	Level (x_2)	Value	t_0	t_4	t_7	t_{14}
E1	0	25°C	0	9.0	180 ± 1	554 ± 8	772 ± 6	933 ± 7
E2	0	25°C	0	9.0	170 ± 1	574 ± 2	747 ± 2	892 ± 3
E3	+α	32°C	0	9.0	247 ± 2	410 ± 10	540 ± 10	675 ± 3
E4	−α	18°C	0	9.0	216 ± 2	690 ± 10	850 ± 40	1150 ± 40
E5	−1	20°C	−1	3.0	182 ± 1	617 ± 5	745 ± 3	980 ± 30
E6	+1	30°C	−1	3.0	177 ± 1	463 ± 6	615 ± 5	800 ± 20
E7	−1	20°C	+1	27.0	165 ± 1	625 ± 1	760 ± 20	1050 ± 30
E8	+1	30°C	+1	27.0	174 ± 1	446 ± 1	652 ± 4	747 ± 9
E9	0	25°C	−α	1.9	230 ± 1	569 ± 3	690 ± 20	875 ± 3
E10	0	25°C	+α	42.6	191 ± 1	569 ± 5	756 ± 2	840 ± 10
E11	0	25°C	0	9.0	207.0 ± 1	618 ± 3	820 ± 10	1020 ± 60
E12	0	25°C	0	9.0	204 ± 1	547 ± 7	710 ± 10	790 ± 30

Each value is presented in the form: mean ± standard error (SE). SE were calculated with the original replicas.

Table S2: Total protein content (% w/w), at days 0, 4, 7 and 14, and total protein average productivity, P_{Avg} (mg L^{-1} d^{-1}) at the end of days 4, 7 and 14, for the 12 points of the experimental design.

	Total protein content (% w/w)				Total protein P_{Avg} (mg L^{-1} d^{-1})		
	t_0	t_4	t_7	t_{14}	$t_0 \rightarrow t_4$	$t_0 \rightarrow t_7$	$t_0 \rightarrow t_{14}$
E1	12.3 ± 0.7	20.0 ± 1.0	18.1 ± 0.4	13.0 ± 0.6	23.0 ± 1.0	17.5 ± 0.5	7.2 ± 0.4
E2	12.3 ± 0.7	21.4 ± 0.1	18.8 ± 0.8	15.1 ± 0.6	27.1 ± 0.3	17.7 ± 0.9	8.3 ± 0.4
E3	12.0 ± 1.0	14.0 ± 4.0	8.0 ± 2.0	13.0 ± 0.3	8.0 ± 4.0	2.0 ± 2.0	4.3 ± 0.3
E4	16.0 ± 3.0	20.0 ± 1.0	17.8 ± 0.9	14.0 ± 1.0	26.0 ± 3.0	17.0 ± 2.0	9.0 ± 1.0
E5	13.2 ± 0.1	17.0 ± 1.0	10.2 ± 0.8	10.3 ± 0.3	22.0 ± 2.0	7.7 ± 0.8	5.6 ± 0.3
E6	13.2 ± 0.1	18.0 ± 0.7	13.9 ± 0.5	9.0 ± 0.3	16.2 ± 0.9	9.3 ± 0.4	3.6 ± 0.2
E7	13.2 ± 0.5	19.0 ± 1.0	13.8 ± 0.5	12.7 ± 0.2	25.0 ± 2.0	12.2 ± 0.7	8.0 ± 0.3
E8	13.2 ± 0.5	14.6 ± 0.5	15.8 ± 0.9	12.6 ± 0.6	11.2 ± 0.6	11.8 ± 0.8	5.2 ± 0.4
E9	11.6 ± 0.1	12.2 ± 0.3	8.5 ± 0.7	7.0 ± 2.0	11.7 ± 0.9	4.8 ± 0.9	3.0 ± 1.0
E10	13.2 ± 0.6	17.1 ± 0.2	14.4 ± 0.1	13.0 ± 1.0	19.1 ± 0.3	12.4 ± 0.1	5.8 ± 0.6
E11	8.9 ± 0.6	11.0 ± 0.1	10.8 ± 0.6	9.0 ± 1.0	13.3 ± 0.4	10.6 ± 0.7	5.3 ± 0.9
E12	8.9 ± 0.6	10.8 ± 0.6	10.0 ± 1.0	8.8 ± 0.1	11.1 ± 0.9	8.0 ± 1.0	3.7 ± 0.2

Each value is presented in the form: mean ± standard error (SE). SE were calculated with the original replicas for the content (% w/w) values, and using error propagation formulas for productivity (mg L^{-1} d^{-1}) values.

Table S3: Total fatty acids content (% w/w) at days 0, 4, 7 and 14, and total fatty acids average productivity, P_{Avg} (mg L^{-1} d^{-1}) at the end of days 4, 7 and 14, for the 12 points of the experimental design.

	Total fatty acids content (% w/w)			Total fatty acids P_{Avg} (mg L^{-1} d^{-1})	
	t_0	t_7	t_{14}	$t_0 \rightarrow t_7$	$t_0 \rightarrow t_{14}$
E1	11 ± 1	16 ± 2	31 ± 4	15 ± 2	19 ± 1
E2	11 ± 1	16 ± 1	29 ± 3	14 ± 1	17 ± 1
E3	13 ± 1	13 ± 1	15 ± 1	5 ± 1	5 ± 1
E4	13 ± 1	n.d.	29 ± 2	n.d.	22 ± 1
E5	14 ± 1	31 ± 2	36 ± 3	31 ± 2	24 ± 1
E6	14 ± 1	17 ± 1	28 ± 1	12 ± 1	15 ± 1
E7	14 ± 1	20 ± 2	34 ± 4	19 ± 3	24 ± 1
E8	14 ± 1	18 ± 1	25 ± 3	14 ± 1	12 ± 1
E9	15 ± 1	20 ± 2	36 ± 3	9 ± 1	15 ± 1
E10	15 ± 1	19 ± 1	28 ± 1	18 ± 2	15 ± 1

n.d.: non determined due to experimental issues.

Table S4: Total carbohydrate content (% w/w) at days 0, 4, 7 and 14, and total carbohydrate average productivity, P_{Avg} (mg L^{-1} d^{-1}) at the end of days 4, 7 and 14, for the 12 points of the experimental design.

	Total carbohydrate content (% w/w)				Total carbohydrate P_{Avg} (mg L^{-1} d^{-1})		
	t_0	t_4	t_7	t_{14}	$t_0 \rightarrow t_4$	$t_0 \rightarrow t_7$	$t_0 \rightarrow t_{14}$
E1	7.6 ± 0.6	12.0 ± 1.0	13.6 ± 0.2	9.3 ± 0.3	14.0 ± 2.0	13.5 ± 0.3	5.3 ± 0.2
E2	7.6 ± 0.6	11.3 ± 0.2	12.3 ± 0.4	11.0 ± 0.6	13.7 ± 0.3	11.7 ± 0.5	6.2 ± 0.4
E3	7.6 ± 0.1	10.7 ± 0.2	9.8 ± 0.1	8.2 ± 0.3	7.1 ± 0.3	5.0 ± 0.2	2.7 ± 0.2
E4	9.0 ± 1.0	11.2 ± 0.1	13.3 ± 0.2	16.1 ± 0.7	14.3 ± 0.8	13.7 ± 0.8	12.0 ± 0.7
E5	8.2 ± 0.5	13.4 ± 0.1	13.2 ± 0.1	11.9 ± 0.9	18.4 ± 0.3	12.5 ± 0.2	7.5 ± 0.7
E6	8.2 ± 0.5	n.d.	15.6 ± 0.9	13.9 ± 0.4	n.d.	12.1 ± 0.8	7.1 ± 0.3
E7	11.7 ± 0.6	12.2 ± 0.8	11.1 ± 0.3	10.7 ± 0.4	15.0 ± 1.0	9.6 ± 0.5	6.7 ± 0.4
E8	11.7 ± 0.6	11.6 ± 0.5	15.0 ± 2.0	10.3 ± 0.4	8.4 ± 0.7	11.0 ± 1.0	4.1 ± 0.2
E9	7.6 ± 0.1	11.8 ± 0.6	9.3 ± 0.7	6.3 ± 1.0	13.7 ± 0.9	7.0 ± 0.7	2.7 ± 0.6
E10	9.8 ± 0.1	12.1 ± 0.2	13.4 ± 0.1	10.9 ± 0.5	13.3 ± 0.3	12.2 ± 0.1	5.2 ± 0.3
E11	8.6 ± 0.1	13.0 ± 3.0	11.0 ± 10.0	14.0 ± 1.0	17.0 ± 4.0	10.0 ± 10.0	9.0 ± 1.0
E12	8.6 ± 0.1	11.0 ± 9.0	22.0 ± 3.0	15.8 ± 0.4	10.0 ± 10.0	20.0 ± 3.0	7.8 ± 0.4

n.d.: non determined due to experimental issues.

Table S5: Total pigment content (% w/w) at days 0, 4, 7 and 14, and total pigment average productivity, P_{Avg} (mg L^{-1} d^{-1}) at the end of days 4, 7 and 14.

	Total pigment content (% w/w)				Total pigment P_{Avg} (mg L^{-1} d^{-1})		
	t_0	t_4	t_7	t_{14}	$t_0 \rightarrow t_4$	$t_0 \rightarrow t_7$	$t_0 \rightarrow t_{14}$
E1	1.08 ± 0.07	1.25 ± 0.01	0.34 ± 0.02	0.10 ± 0.01	1.32 ± 0.04	0.10 ± 0.03	-0.07 ± 0.01
E2	1.08 ± 0.07	1.60 ± 0.07	0.63 ± 0.02	0.12 ± 0.01	1.96 ± 0.1	0.43 ± 0.03	-0.06 ± 0.01
E3	0*	0.03 ± 0.01	0.024 ± 0.002	0.20 ± 0.01	0.04 ± 0.01	0.024 ± 0.002	0.10 ± 0.01
E4	0.68 ± 0.04	0.84 ± 0.01	1.37 ± 0.09	0.21 ± 0.01	1.10 ± 0.04	1.5 ± 0.1	0.07 ± 0.01
E5	0.68 ± 0.02	1.32 ± 0.01	0.62 ± 0.06	0.27 ± 0.01	1.86 ± 0.02	0.50 ± 0.07	0.11 ± 0.01
E6	0.68 ± 0.02	0.61 ± 0.01	0.22 ± 0.01	0.06 ± 0.01	0.43 ± 0.01	0.02 ± 0.01	-0.05 ± 0.01
E7	0.60 ± 0.03	1.46 ± 0.05	0.57 ± 0.04	0.11 ± 0.01	2.17 ± 0.07	0.49 ± 0.04	0.01 ± 0.01
E8	0.60 ± 0.03	0.44 ± 0.01	0.34 ± 0.01	0.03 ± 0.01	0.24 ± 0.02	0.18 ± 0.01	-0.06 ± 0.01
E9	0*	0.70 ± 0.20	0.31 ± 0.01	0.14 ± 0.01	1.10 ± 0.20	0.32 ± 0.02	0.09 ± 0.01
E10	0.28 ± 0.04	1.04 ± 0.01	0.55 ± 0.02	0.07 ± 0.01	1.43 ± 0.03	0.53 ± 0.02	0.01 ± 0.01
E11	0.23 ± 0.01	0.93 ± 0.02	0.71 ± 0.09	0.17 ± 0.01	1.41 ± 0.04	0.80 ± 0.10	0.09 ± 0.01
E12	0.23 ± 0.01	0.81 ± 0.08	0.52 ± 0.04	0.24 ± 0.02	1.10 ± 0.10	0.48 ± 0.04	0.10 ± 0.01

* below detection limit

Table S6: Chlorophyll a content (% w/w) at days 0, 4, 7 and 14, and chlorophyll a average productivity, P_{Avg} (mg L^{-1} d^{-1}) at the end of days 4, 7 and 14.

	Chlorophyll a content (% w/w)				Chlorophyll a P_{Avg} (mg L^{-1} d^{-1})		
	t_0	t_4	t_7	t_{14}	$t_0 \rightarrow t_4$	$t_0 \rightarrow t_7$	$t_0 \rightarrow t_{14}$
E1	0.60 ± 0.04	0.64 ± 0.01	0.10 ± 0.01	0.03 ± 0.01	0.65 ± 0.02	-0.04 ± 0.01	-0.06 ± 0.01
E2	0.60 ± 0.04	0.86 ± 0.04	0.24 ± 0.01	0.03 ± 0.01	1.04 ± 0.06	0.11 ± 0.02	-0.05 ± 0.01
E3	0*	0.02 ± 0.01	0.01 ± 0.01	0.07 ± 0.01	0.02 ± 0.01	0.01 ± 0.02	0.04 ± 0.01
E4	0.35 ± 0.02	0.46 ± 0.01	0.78 ± 0.06	0.05 ± 0.01	0.61 ± 0.03	0.87 ± 0.08	-0.01 ± 0.01
E5	0.38 ± 0.01	0.71 ± 0.01	0.32 ± 0.03	0.09 ± 0.01	0.98 ± 0.01	0.26 ± 0.03	0.02 ± 0.01
E6	0.38 ± 0.01	0.31 ± 0.01	0.08 ± 0.01	0.00 ± 0.01	0.21 ± 0.01	-0.03 ± 0.01	-0.05± 0.01
E7	0.30 ± 0.01	0.81 ± 0.02	0.22 ± 0.02	0.03 ± 0.01	1.21 ± 0.04	0.17 ± 0.02	-0.01 ± 0.01
E8	0.30 ± 0.01	0.21 ± 0.01	0.10 ± 0.01	0.00 ± 0.01	0.11 ± 0.01	0.02 ± 0.01	-0.04 ± 0.01
E9	0*	0.29 ± 0.04	0.11 ± 0.01	0.03 ± 0.01	0.45 ± 0.05	0.11 ± 0.01	0.02 ± 0.01
E10	0.13 ± 0.03	0.53 ± 0.01	0.19 ± 0.01	0.02 ± 0.01	0.74 ± 0.01	0.18 ± 0.01	-0.01 ± 0.01
E11	0.09 ± 0.01	0.42 ± 0.02	0.28 ± 0.07	0.05 ± 0.01	0.65 ± 0.03	0.32 ± 0.08	0.02 ± 0.01
E12	0.09 ± 0.01	0.36 ± 0.04	0.18 ± 0.01	0.06 ± 0.01	0.48 ± 0.06	0.16 ± 0.01	0.02 ± 0.01

Table S7: Chlorophyll b content (% w/w) at days 0, 4, 7 and 14, and chlorophyll b average productivity, P_{Avg} (mg L^{-1} d^{-1}) at the end of days 4, 7 and 14.

	Chlorophyll b content (% w/w)				Chlorophyll b P_{Avg} (mg L^{-1} d^{-1})		
	t_0	t_4	t_7	t_{14}	$t_0 \rightarrow t_4$	$t_0 \rightarrow t_7$	$t_0 \rightarrow t_{14}$
E1	0.23 ± 0.02	0.35 ± 0.01	0.08 ± 0.01	0.03 ± 0.01	0.40 ± 0.01	0.027 ± 0.01	-0.01 ± 0.01
E2	0.23 ± 0.02	0.44 ± 0.02	0.15 ± 0.01	0.03 ± 0.01	0.57 ± 0.03	0.11 ± 0.01	-0.01 ± 0.01
E3	0*	-0.02 ± 0.01	-0.03 ± 0.01	0.02 ± 0.01	0.01 ± 0.01	-0.01 ± 0.01	0.01 ± 0.01
E4	0.15 ± 0.01	0.26 ± 0.01	0.19 ± 0.01	0.05 ± 0.01	0.37 ± 0.02	0.19 ± 0.02	0.02 ± 0.01
E5	0.14 ± 0.01	0.32 ± 0.01	0.19 ± 0.02	0.08 ± 0.01	0.46 ± 0.01	0.17 ± 0.02	0.04 ± 0.01
E6	0.14 ± 0.01	0.15 ± 0.01	0.04 ± 0.01	0.01 ± 0.01	0.12 ± 0.01	0.00 ± 0.01	-0.01 ± 0.01
E7	0.123 ± 0.01	0.33 ± 0.01	0.12 ± 0.01	0.03 ± 0.01	0.50 ± 0.01	0.11 ± 0.01	0.01 ± 0.01
E8	0.123 ± 0.01	0.09 ± 0.01	0.07 ± 0.01	0.01 ± 0.01	0.06 ± 0.01	0.04 ± 0.01	-0.01 ± 0.01
E9	0*	0.20 ± 0.10	0.00 ± 0.01	0.00 ± 0.01	0.30 ± 0.20	0.01 ± 0.01	0.01 ± 0.01
E10	0.06 ± 0.01	0.24 ± 0.01	0.12 ± 0.01	0.02 ± 0.01	0.33 ± 0.01	0.11 ± 0.01	0.00 ± 0.02
E11	0.06 ± 0.01	0.26 ± 0.01	0.19 ± 0.01	0.05 ± 0.01	0.39 ± 0.01	0.21 ± 0.01	0.02 ± 0.01
E12	0.06 ± 0.01	0.22 ± 0.01	0.13 ± 0.01	0.05 ± 0.01	0.29 ± 0.02	0.12 ± 0.01	0.02 ± 0.01

*below detection limit

Table S8: Total carotenoid content (% w/w) at days 0, 4, 7 and 14, and total carotenoid average productivity, P_{Avg} (mg L^{-1} d^{-1}) at the end of days 4, 7 and 14.

	Total carotenoid content (% w/w)				Total carotenoid P_{Avg} (mg L^{-1} d^{-1})		
	t_0	t_4	t_7	t_{14}	$t_0 \rightarrow t_4$	$t_0 \rightarrow t_7$	$t_0 \rightarrow t_{14}$
E1	0.25 ± 0.01	0.26 ± 0.01	0.16 ± 0.01	0.04 ± 0.01	0.27 ± 0.02	0.11 ± 0.01	-0.01 ± 0.01
E2	0.25 ± 0.01	0.30 ± 0.01	0.24 ± 0.01	0.05 ± 0.01	0.35 ± 0.01	0.20 ± 0.01	0.00 ± 0.01
E3	0.02 ± 0.01	0.03 ± 0.01	0.04 ± 0.01	0.11 ± 0.01	0.02 ± 0.01	0.02 ± 0.01	0.05 ± 0.01
E4	0.18 ± 0.01	0.13 ± 0.01	0.41 ± 0.02	0.11 ± 0.01	0.12 ± 0.01	0.46 ± 0.03	0.06 ± 0.01
E5	0.16 ± 0.01	0.28 ± 0.01	0.11 ± 0.01	0.10 ± 0.01	0.39 ± 0.01	0.07 ± 0.01	0.05 ± 0.01
E6	0.16 ± 0.01	0.14 ± 0.01	0.10 ± 0.01	0.05 ± 0.01	0.10 ± 0.01	0.05 ± 0.01	0.01 ± 0.01
E7	0.17 ± 0.01	0.32 ± 0.02	0.23 ± 0.01	0.04 ± 0.01	0.45 ± 0.02	0.21 ± 0.01	0.01 ± 0.01
E8	0.17 ± 0.01	0.14 ± 0.01	0.17 ± 0.01	0.02 ± 0.01	0.08 ± 0.01	0.12 ± 0.01	-0.01 ± 0.01
E9	0.02 ± 0.01	0.21 ± 0.03	0.20 ± 0.01	0.10 ± 0.01	0.32 ± 0.04	0.20 ± 0.01	0.06 ± 0.01
E10	0.09 ± 0.01	0.27 ± 0.01	0.24 ± 0.01	0.03 ± 0.01	0.36 ± 0.01	0.24 ± 0.01	0.01 ± 0.01
E11	0.08 ± 0.01	0.25 ± 0.01	0.24 ± 0.01	0.08 ± 0.01	0.37 ± 0.01	0.27 ± 0.01	0.05 ± 0.01
E12	0.08 ± 0.01	0.23 ± 0.03	0.21 ± 0.02	0.13 ± 0.01	0.30 ± 0.04	0.20 ± 0.02	0.06 ± 0.01

Table S9: C16:0 (Palmitic acid) content (% w/w) at days 0, 7 and 14, and C16:0 average productivity, P_{Avg} (mg L^{-1} d^{-1}), at the end of days 7 and 14.

	C16:0 content (% w/w)			C16:0 P_{Avg} (mg L^{-1} d^{-1})	
	t_0	t_7	t_{14}	$t_0 \rightarrow t_7$	$t_0 \rightarrow t_{14}$
E1	2.0 ± 0.2^{a}	3.3 ± 0.4^{bc}	6.8 ± 0.8^{fg}	3.3 ± 0.5^{cd}	4.4 ± 0.6^{de}
E2	2.0 ± 0.2^{a}	3.4 ± 0.2^{bc}	6.5 ± 0.6^{fg}	3.2 ± 0.2^{cd}	3.9 ± 0.4^{d}
E3	2.3 ± 0.2^{a}	3.3 ± 0.1^{bc}	4.0 ± 0.4^{bcd}	-0.2 ± 0.1^{a}	-0.2 ± 0.1^{a}
E4	2.3 ± 0.2^{a}	n.d.	5.5 ± 0.4^{ef}	n.d.	4.3 ± 0.4^{de}
E5	2.6 ± 0.1^{abc}	5.4 ± 0.4^{e}	7.1 ± 0.5^{g}	5.3 ± 0.4^{e}	4.7 ± 0.4^{de}
E6	2.6 ± 0.1^{abc}	4.1 ± 0.2^{cd}	6.3 ± 0.1^{f}	3.1 ± 0.2^{c}	3.3 ± 0.1^{cd}
E7	2.6 ± 0.1^{ab}	3.6 ± 0.4^{bcd}	6.7 ± 0.9^{fg}	3.4 ± 0.5^{cd}	4.8 ± 0.7^{de}
E8	2.6 ± 0.1^{ab}	4.3 ± 0.3^{d}	6.2 ± 0.8^{f}	3.5 ± 0.3^{cd}	3.0 ± 0.4^{cd}
E9	2.7 ± 0.1^{abc}	3.8 ± 0.4^{bcd}	7.0 ± 0.7^{fg}	1.7 ± 0.2^{b}	3.0 ± 0.3^{cd}
E10	2.7 ± 0.1^{abc}	3.9 ± 0.3^{bcd}	6.0 ± 0.2^{ef}	3.5 ± 0.3^{cd}	3.2 ± 0.2^{cd}

Each value is presented in the form: mean ± standard deviation (SD).
Different lowercase letters indicate statistically significant differences between values ($p < 0.05$). For content values, letters were determined from two-way ANOVA followed by Tukey HSD post-hoc comparisons. For productivity values, letters were determined using pairwise z-tests based on propagated standard deviations.

Table S10: C18:0 (Steraic acid) content (% w/w) at days 0, 7 and 14, and C18:0 average productivity, P_{Avg} (mg L^{-1} d^{-1}), at the end of days 7 and 14.

	C18:0 content (% w/w)			C18:0 P_{Avg} (mg L^{-1} d^{-1})	
	t_0	t_7	t_{14}	$t_0 \rightarrow t_7$	$t_0 \rightarrow t_{14}$
E1	0.09 ± 0.01^{a}	0.33 ± 0.05^{c}	0.80 ± 0.10^{h}	0.35 ± 0.05^{de}	0.53 ± 0.07^{f}
E2	0.09 ± 0.01^{a}	0.33 ± 0.02^{c}	0.69 ± 0.06^{fg}	0.35 ± 0.02^{de}	0.44 ± 0.04^{ef}
E3	0.16 ± 0.01^{ab}	0.10 ± 0.01^{a}	0.17 ± 0.02^{b}	-0.03 ± 0.01^{a}	-0.02 ± 0.01^{b}
E4	0.16 ± 0.01^{ab}	n.d.	0.64 ± 0.05^{f}	n.d.	0.51 ± 0.04^{f}
E5	0.15 ± 0.01^{ab}	0.63 ± 0.03^{f}	0.68 ± 0.02^{fg}	0.66 ± 0.04^{g}	0.46 ± 0.02^{f}
E6	0.15 ± 0.01^{ab}	0.45 ± 0.02^{d}	0.57 ± 0.01^{ef}	0.37 ± 0.02^{e}	0.31 ± 0.01^{d}
E7	0.18 ± 0.01^{b}	0.40 ± 0.04^{d}	0.61 ± 0.08^{ef}	0.40 ± 0.05^{def}	0.44 ± 0.06^{ef}
E8	0.18 ± 0.01^{b}	0.45 ± 0.02^{d}	0.56 ± 0.08^{e}	0.39 ± 0.02^{e}	0.28 ± 0.04^{d}
E9	0.20 ± 0.01^{b}	0.28 ± 0.02^{c}	0.67 ± 0.06^{fg}	0.13 ± 0.01^{c}	0.30 ± 0.03^{d}
E10	0.20 ± 0.01^{b}	0.72 ± 0.03^{g}	0.87 ± 0.03^{h}	0.74 ± 0.04^{g}	0.49 ± 0.02^{f}

Different lowercase letters indicate significant differences ($p < 0.05$; see Table S9).

Table S11: C16:1n9 (Palmitoleic acid) content (% w/w) at days 0, 7 and 14, and C16:1n9 average productivity, P_{Avg} (mg L^{-1} d^{-1}), at the end of days 7 and 14.

C16:1n9 content (% w/w)	C16:1n9 P_{Avg} (mg L^{-1} d^{-1})

	t_0	t_7	t_{14}	$t_0 \rightarrow t_7$	$t_0 \rightarrow t_{14}$
E1	0.10 ± 0.04^{a}	0.12 ± 0.02^{abcd}	0.22 ± 0.03^{ef}	0.11 ± 0.02^{cd}	0.13 ± 0.02^{de}
E2	0.10 ± 0.04^{a}	0.12 ± 0.01^{abcd}	0.21 ± 0.02^{ef}	0.11 ± 0.01^{cd}	0.13 ± 0.01^{de}
E3	0.11 ± 0.04^{abc}	0.13 ± 0.01^{abcd}	0.17 ± 0.01^{bcde}	-0.01 ± 0.01^{a}	-0.01 ± 0.01^{a}
E4	0.11 ± 0.04^{abc}	n.d.	0.20 ± 0.01^{cdef}	n.d.	0.15 ± 0.01^{de}
E5	0.14 ± 0.01^{abcde}	0.15 ± 0.01^{bcde}	0.20 ± 0.01^{def}	0.13 ± 0.01^{de}	0.13 ± 0.01^{de}
E6	0.14 ± 0.01^{abcde}	0.11 ± 0.01^{ab}	0.18 ± 0.01^{bcdef}	0.06 ± 0.01^{b}	0.09 ± 0.01^{cd}
E7	0.15 ± 0.01^{bcde}	0.13 ± 0.02^{abcde}	0.32 ± 0.05^{g}	0.11 ± 0.02^{cde}	0.23 ± 0.03^{f}
E8	0.15 ± 0.01^{bcde}	0.16 ± 0.01^{bcde}	0.22 ± 0.03^{f}	0.11 ± 0.01^{cd}	0.10 ± 0.01^{cd}
E9	0.13 ± 0.04^{abcde}	0.16 ± 0.02^{bcde}	0.22 ± 0.02^{ef}	0.07 ± 0.02^{bc}	0.09 ± 0.01^{cd}
E10	0.13 ± 0.04^{abcde}	0.16 ± 0.01^{bcde}	0.28 ± 0.01^{g}	0.14 ± 0.02^{de}	0.15 ± 0.01^{e}

Different lowercase letters indicate significant differences ($p < 0.05$; see Table S9).

Table S12: C16:1n7 (Palmitoleic acid) content (% w/w) at days 0, 7 and 14, and C16:1n7 average productivity, P_{Avg} (mg L^{-1} d^{-1}), at the end of days 7 and 14.

	C16:1n7 content (% w/w)			C16:1n7 P_{Avg} (mg L^{-1} d^{-1})	
	t_0	t_7	t_{14}	$t_0 \rightarrow t_7$	$t_0 \rightarrow t_{14}$
E1	0.058 ± 0.008^{a}	0.063 ± 0.008^{ab}	0.090 ± 0.010^{cde}	0.057 ± 0.010^{def}	0.056 ± 0.008^{de}
E2	0.058 ± 0.008^{a}	0.066 ± 0.003^{ab}	0.099 ± 0.009^{cde}	0.058 ± 0.004^{de}	0.057 ± 0.006^{de}
E3	0.057 ± 0.006^{a}	0.145 ± 0.005^{g}	0.150 ± 0.010^{g}	0.003 ± 0.002^{b}	-0.005 ± 0.001^{a}
E4	0.057 ± 0.006^{a}	n.d.	0.088 ± 0.006^{bcde}	n.d.	0.065 ± 0.006^{ef}
E5	0.065 ± 0.002^{ab}	0.107 ± 0.008^{ef}	0.200 ± 0.010^{h}	0.101 ± 0.009^{g}	0.130 ± 0.010^{h}
E6	0.065 ± 0.002^{ab}	0.098 ± 0.003^{cde}	0.160 ± 0.002^{g}	0.073 ± 0.003^{ef}	0.085 ± 0.002^{g}
E7	0.063 ± 0.002^{ab}	0.059 ± 0.007^{a}	0.100 ± 0.010^{de}	0.051 ± 0.008^{cde}	0.070 ± 0.010^{def}
E8	0.063 ± 0.002^{ab}	0.084 ± 0.007^{bcd}	0.120 ± 0.010^{f}	0.066 ± 0.007^{def}	0.057 ± 0.007^{de}
E9	0.069 ± 0.003^{abc}	0.120 ± 0.010^{f}	0.200 ± 0.020^{h}	0.058 ± 0.008^{def}	0.088 ± 0.009^{fg}
E10	0.069 ± 0.003^{abc}	0.061 ± 0.005^{ab}	0.076 ± 0.003^{bcd}	0.049 ± 0.006^{d}	0.036 ± 0.002^{c}

Different lowercase letters indicate significant differences ($p < 0.05$; see Table S9).

Table S13: C18:1n9c (Oleic acid) content (% w/w) at days 0, 7 and 14, and C18:1n9c average productivity, P_{Avg} (mg L^{-1} d^{-1}), at the end of days 7 and 14.

	C18:1n9c content (% w/w)			C18:1n9c P_{Avg} (mg L^{-1} d^{-1})	
	t_0	t_7	t_{14}	$t_0 \rightarrow t_7$	$t_0 \rightarrow t_{14}$
E1	1.7 ± 0.2^{bc}	2.6 ± 0.4^{bcd}	8.6 ± 1.1^{jkl}	2.6 ± 0.4^{bc}	5.6 ± 0.7^{def}
E2	1.7 ± 0.2^{bc}	2.4 ± 0.1^{bc}	7.4 ± 0.6^{hij}	2.2 ± 0.1^{b}	4.6 ± 0.4^{de}
E3	2.2 ± 0.2^{bc}	1.6 ± 0.1^{b}	3.3 ± 0.3^{cde}	-0.3 ± 0.1^{a}	-0.2 ± 0.0^{a}
E4	2.2 ± 0.2^{bc}	n.d.	9.7 ± 0.7^{kl}	n.d.	7.7 ± 0.7^{fg}
E5	2.7 ± 0.1^{cd}	10.9 ± 0.7^{m}	12.8 ± 0.8^{n}	11.4 ± 0.7^{h}	8.8 ± 0.7^{g}
E6	2.7 ± 0.1^{cd}	4.0 ± 0.2^{def}	8.3 ± 0.2^{ijk}	3.0 ± 0.2^{c}	4.5 ± 0.1^{d}
E7	2.6 ± 0.1^{bcd}	5.0 ± 0.6^{efg}	17.0 ± 1.0^{lm}	4.9 ± 0.6^{def}	8.0 ± 1.0^{fg}
E8	2.6 ± 0.1^{bcd}	3.7 ± 0.2^{cde}	6.9 ± 0.9^{ghi}	2.9 ± 0.2^{c}	3.4 ± 0.5^{c}
E9	3.2 ± 0.1^{cde}	6.0 ± 0.5^{fgh}	14.0 ± 1.0^{o}	3.1 ± 0.4^{c}	6.4 ± 0.7^{ef}
E10	3.2 ± 0.1^{cde}	4.9 ± 0.3^{efg}	9.4 ± 0.4^{jkl}	4.6 ± 0.3^{de}	5.2 ± 0.2^{ef}

Different lowercase letters indicate significant differences ($p < 0.05$; see Table S9).

Table S14: C18:1n7c content (% w/w) at days 0, 7 and 14, and C18:1n7c average productivity, P_{Avg} (mg L^{-1} d^{-1}), at the end of days 7 and 14.

	C18:1n7c content (% w/w)			C18:1n7c P_{Avg} (mg L^{-1} d^{-1})	
	t_0	t_7	t_{14}	$t_0 \rightarrow t_7$	$t_0 \rightarrow t_{14}$

E1	0.16 ± 0.02^{bc}	0.13 ± 0.02^{b}	0.22 ± 0.03^{cde}	0.11 ± 0.02^{bc}	0.13 ± 0.02^{bc}
E2	0.16 ± 0.02^{bc}	0.12 ± 0.01^{b}	0.22 ± 0.02^{cde}	0.09 ± 0.01^{b}	0.12 ± 0.01^{c}
E3	0.16 ± 0.02^{bc}	0.26 ± 0.01^{efg}	0.30 ± 0.03^{fg}	-0.01 ± 0.01^{a}	-0.02 ± 0.01^{a}
E4	0.16 ± 0.02^{bc}	n.d.	0.27 ± 0.02^{efg}	n.d.	0.2 ± 0.02^{de}
E5	0.20 ± 0.01^{cde}	0.36 ± 0.03^{h}	0.46 ± 0.05^{i}	0.34 ± 0.03^{f}	0.31 ± 0.04^{ef}
E6	0.20 ± 0.01^{cde}	0.22 ± 0.01^{cde}	0.34 ± 0.01^{gh}	0.15 ± 0.01^{c}	0.17 ± 0.01^{de}
E7	0.23 ± 0.01^{cdef}	0.19 ± 0.02^{cd}	0.33 ± 0.04^{gh}	0.15 ± 0.02^{cde}	0.23 ± 0.03^{de}
E8	0.23 ± 0.01^{cdef}	0.21 ± 0.02^{cde}	0.29 ± 0.04^{fg}	0.15 ± 0.02^{c}	0.13 ± 0.02^{bc}
E9	0.25 ± 0.01^{def}	0.33 ± 0.03^{gh}	0.52 ± 0.05^{j}	0.15 ± 0.02^{cd}	0.22 ± 0.03^{de}
E10	0.25 ± 0.01^{def}	0.15 ± 0.01^{bc}	0.22 ± 0.01^{cde}	0.10 ± 0.02^{b}	0.10 ± 0.01^{b}

Different lowercase letters indicate significant differences ($p < 0.05$; see Table S9).

Table S15: C16:2 content (% w/w) at days 0, 7 and 14, and C16:2 average productivity, P_{Avg} (mg L^{-1} d^{-1}), at the end of days 7 and 14.

	C16:2 content (% w/w)			C16:2 P_{Avg} (mg L^{-1} d^{-1})	
	t_0	t_7	t_{14}	$t_0 \rightarrow t_7$	$t_0 \rightarrow t_{14}$
E1	0.31 ± 0.05^{bc}	0.90 ± 0.10^{ghi}	1.20 ± 0.10^{j}	0.90 ± 0.10^{gh}	0.80 ± 0.10^{fgh}
E2	0.31 ± 0.05^{bc}	0.88 ± 0.04^{ghi}	1.30 ± 0.20^{j}	0.89 ± 0.05^{h}	0.80 ± 0.10^{fgh}
E3	0.47 ± 0.06^{cde}	0.38 ± 0.01^{bcd}	0.25 ± 0.02^{b}	-0.07 ± 0.01^{a}	-0.05 ± 0.01^{a}
E4	0.47 ± 0.06^{cde}	n.d.	0.71 ± 0.05^{efg}	n.d.	0.52 ± 0.05^{ef}
E5	0.53 ± 0.02^{cde}	0.77 ± 0.03^{fgh}	0.76 ± 0.01^{fgh}	0.71 ± 0.04^{g}	0.47 ± 0.02^{e}
E6	0.53 ± 0.02^{cde}	0.61 ± 0.01^{def}	0.68 ± 0.01^{efg}	0.42 ± 0.01^{d}	0.33 ± 0.01^{c}
E7	0.53 ± 0.02^{cde}	0.77 ± 0.09^{fgh}	1.10 ± 0.20^{ij}	0.70 ± 0.10^{fgh}	0.80 ± 0.20^{fgh}
E8	0.53 ± 0.02^{cde}	0.82 ± 0.06^{fgh}	0.75 ± 0.08^{efgh}	0.66 ± 0.06^{fg}	0.34 ± 0.04^{cd}
E9	0.49 ± 0.02^{cde}	0.43 ± 0.05^{cd}	0.70 ± 0.07^{efg}	0.14 ± 0.03^{b}	0.27 ± 0.03^{c}
E10	0.49 ± 0.02^{cde}	1.00 ± 0.09^{hi}	1.12 ± 0.03^{ij}	0.98 ± 0.1^{h}	0.60 ± 0.02^{f}

Different lowercase letters indicate significant differences ($p < 0.05$; see Table S9).

Table S16: C16:3 content (% w/w) at days 0, 7 and 14, and C16:3 average productivity, P_{Avg} (mg L^{-1} d^{-1}), at the end of days 7 and 14.

	C16:3 content (% w/w)			C16:3 P_{Avg} (mg L^{-1} d^{-1})	
	t_0	t_7	t_{14}	$t_0 \rightarrow t_7$	$t_0 \rightarrow t_{14}$
E1	0.070 ± 0.007^{bcd}	0.058 ± 0.007^{ab}	0.100 ± 0.010^{e}	0.048 ± 0.009^{cde}	0.057 ± 0.009^{e}
E2	0.070 ± 0.007^{bcd}	0.063 ± 0.003^{bcd}	0.074 ± 0.007^{bcd}	0.052 ± 0.004^{e}	0.039 ± 0.005^{cd}
E3	0.075 ± 0.007^{bcd}	0.065 ± 0.002^{bcd}	0.052 ± 0.005^{ab}	-0.010 ± 0.002^{a}	-0.008 ± 0.001^{a}
E4	0.075 ± 0.007^{bcd}	n.d.	0.070 ± 0.003^{bcd}	n.d.	0.047 ± 0.003^{e}
E5	0.089 ± 0.003^{e}	0.056 ± 0.005^{ab}	0.139 ± 0.009^{f}	0.038 ± 0.006^{cd}	0.088 ± 0.008^{f}
E6	0.089 ± 0.003^{e}	0.045 ± 0.008^{a}	0.084 ± 0.001^{cde}	0.018 ± 0.007^{b}	0.037 ± 0.001^{d}
E7	0.087 ± 0.003^{de}	0.053 ± 0.006^{ab}	0.090 ± 0.010^{e}	0.038 ± 0.007^{cde}	0.060 ± 0.010^{e}
E8	0.087 ± 0.003^{de}	0.063 ± 0.004^{bcd}	0.090 ± 0.010^{e}	0.038 ± 0.004^{cd}	0.037 ± 0.006^{cd}
E9	0.073 ± 0.003^{bcd}	0.059 ± 0.007^{abc}	0.170 ± 0.020^{g}	0.018 ± 0.005^{b}	0.074 ± 0.008^{f}
E10	0.073 ± 0.003^{bcd}	0.055 ± 0.004^{ab}	0.073 ± 0.001^{bcd}	0.041 ± 0.004^{cde}	0.034 ± 0.001^{c}

Different lowercase letters indicate significant differences ($p < 0.05$; see Table S9).

Table S17: C16:4 content (% w/w) at days 0, 7 and 14, and C16:4 average productivity, P_{Avg} (mg L^{-1} d^{-1}), at the end of days 7 and 14.

	C16:4 content (% w/w)			C16:4 P_{Avg} (mg L^{-1} d^{-1})	
	t_0	t_7	t_{14}	$t_0 \rightarrow t_7$	$t_0 \rightarrow t_{14}$
E1	0.7 ± 0.1^{bc}	0.9 ± 0.1^{c}	1.5 ± 0.2^{e}	0.8 ± 0.1^{de}	1.0 ± 0.1^{e}

E2	0.7 ± 0.1^{bc}	0.8 ± 0.1^{c}	1.4 ± 0.1^{e}	0.8 ± 0.1^{e}	0.8 ± 0.1^{e}
E3	0.8 ± 0.1^{c}	0.7 ± 0.1^{bc}	0.5 ± 0.1^{b}	-0.1 ± 0.02^{a}	-0.1 ± 0.1^{a}
E4	0.8 ± 0.1^{c}	n.d.	2.1 ± 0.1^{g}	n.d.	1.6 ± 0.1^{f}
E5	0.7 ± 0.1^{bc}	2.2 ± 0.2^{gh}	2.4 ± 0.2^{h}	2.2 ± 0.2^{g}	1.6 ± 0.1^{f}
E6	0.7 ± 0.1^{bc}	0.8 ± 0.1^{c}	1.2 ± 0.1^{d}	0.5 ± 0.1^{c}	0.6 ± 0.1^{d}
E7	0.8 ± 0.1^{c}	1.5 ± 0.2^{e}	2.2 ± 0.3^{gh}	1.5 ± 0.2^{f}	1.6 ± 0.2^{f}
E8	0.8 ± 0.1^{c}	0.6 ± 0.1^{b}	0.7 ± 0.1^{bc}	0.4 ± 0.1^{b}	0.3 ± 0.1^{b}
E9	0.8 ± 0.1^{c}	1.2 ± 0.1^{d}	1.8 ± 0.2^{f}	0.6 ± 0.1^{cd}	0.8 ± 0.1^{e}
E10	0.8 ± 0.1^{c}	0.7 ± 0.1^{c}	1.1 ± 0.1^{d}	0.6 ± 0.1^{cd}	0.6 ± 0.1^{cd}

Different lowercase letters indicate significant differences ($p < 0.05$; see Table S9).

Table S18: C18:2cc (Linoleic acid) content (% w/w) at days 0, 7 and 14, and C18:2cc average productivity, P_{Avg} (mg L^{-1} d^{-1}), at the end of days 7 and 14.

	C18:2cc content (% w/w)			C18:2cc P_{Avg} (mg L^{-1} d^{-1})	
	t_0	t_7	t_{14}	$t_0 \rightarrow t_7$	$t_0 \rightarrow t_{14}$
E1	1.3 ± 0.2^{b}	2.8 ± 0.4^{de}	4.3 ± 0.5^{hi}	2.8 ± 0.4^{d}	2.8 ± 0.4^{d}
E2	1.3 ± 0.2^{b}	2.9 ± 0.2^{def}	4.5 ± 0.4^{i}	2.9 ± 0.2^{d}	2.8 ± 0.3^{d}
E3	1.7 ± 0.2^{b}	2.0 ± 0.1^{c}	2.0 ± 0.2^{c}	-0.2 ± 0.1^{a}	-0.2 ± 0.1^{a}
E4	1.7 ± 0.2^{b}	n.d.	2.8 ± 0.2^{de}	n.d.	2.1 ± 0.2^{c}
E5	2.2 ± 0.1^{c}	2.8 ± 0.2^{def}	3.1 ± 0.2^{def}	2.6 ± 0.2^{d}	1.9 ± 0.2^{c}
E6	2.2 ± 0.1^{c}	2.8 ± 0.1^{de}	3.9 ± 0.1^{gh}	1.9 ± 0.1^{c}	2.0 ± 0.1^{c}
E7	1.8 ± 0.1^{bc}	2.3 ± 0.3^{cd}	3.9 ± 0.5^{gh}	2.1 ± 0.3^{c}	2.7 ± 0.4^{d}
E8	1.8 ± 0.1^{bc}	3.7 ± 0.3^{fgh}	4.2 ± 0.5^{hi}	3.1 ± 0.3^{d}	2.1 ± 0.3^{c}
E9	1.9 ± 0.1^{c}	2.2 ± 0.2^{c}	3.3 ± 0.3^{efg}	0.9 ± 0.1^{b}	1.3 ± 0.2^{b}
E10	1.9 ± 0.1^{c}	3.3 ± 0.2^{fg}	3.9 ± 0.1^{gh}	3.2 ± 0.3^{d}	2.1 ± 0.1^{c}

Different lowercase letters indicate significant differences ($p < 0.05$; see Table S9).

Table S19: C18:3n3 (α-linoleic acid) content (% w/w) at days 0, 7 and 14, and C18:3n3 average productivity, P_{Avg} (mg L^{-1} d^{-1}), at the end of days 7 and 14.

	C18:3n3 content (% w/w)			C18:3n3 P_{Avg} (mg L^{-1} d^{-1})	
	t_0	t_7	t_{14}	$t_0 \rightarrow t_7$	$t_0 \rightarrow t_{14}$
E1	3.1 ± 0.5^{b}	3.2 ± 0.4^{b}	5.4 ± 0.6^{efg}	2.8 ± 0.5^{bc}	3.3 ± 0.4^{c}
E2	3.1 ± 0.5^{b}	3.2 ± 0.2^{b}	5.1 ± 0.5^{def}	2.7 ± 0.2^{c}	2.9 ± 0.3^{c}
E3	3.4 ± 0.4^{bc}	3.5 ± 0.1^{bc}	3.1 ± 0.2^{b}	-0.4 ± 0.1^{a}	-0.4 ± 0.1^{a}
E4	3.4 ± 0.4^{bc}	n.d.	5.7 ± 0.4^{efg}	n.d.	4.3 ± 0.4^{d}
E5	3.4 ± 0.1^{bc}	6.4 ± 0.4^{gh}	7.0 ± 0.5^{h}	6.2 ± 0.5^{e}	4.5 ± 0.4^{d}
E6	3.4 ± 0.1^{bc}	3.3 ± 0.1^{b}	5.3 ± 0.1^{ef}	2.1 ± 0.1^{b}	2.6 ± 0.1^{c}
E7	3.6 ± 0.1^{bc}	4.7 ± 0.5^{cdef}	6.9 ± 0.9^{h}	4.4 ± 0.6^{d}	4.8 ± 0.7^{de}
E8	3.6 ± 0.1^{bc}	3.1 ± 0.2^{b}	4.1 ± 0.5^{cd}	2.0 ± 0.2^{b}	1.8 ± 0.3^{b}
E9	3.6 ± 0.1^{bc}	4.6 ± 0.5^{cde}	6.1 ± 0.6^{fg}	2.0 ± 0.3^{b}	2.4 ± 0.3^{bc}
E10	3.6 ± 0.1^{bc}	3.3 ± 0.3^{bc}	4.5 ± 0.2^{cd}	2.7 ± 0.3^{c}	2.2 ± 0.1^{b}

Different lowercase letters indicate significant differences ($p < 0.05$; see Table S9).

Table S20: Neoxanthin content (mg g_{dw}^{-1}) at days 0, 7 and 14, and neoxanthin average productivity, P_{Avg} (mg L^{-1} d^{-1}), at the end of days 7 and 14.

	Neoxanthin content (% w/w)			Neoxanthin P_{Avg} (mg L^{-1} d^{-1})	
	t_0	t_7	t_{14}	$t_0 \rightarrow t_7$	$t_0 \rightarrow t_{14}$
E1	0.241 ± 0.001^{cdefg}	0.21 ± 0.05^{bcdef}	0.11 ± 0.05^{abc}	0.017 ± 0.005^{cd}	0.005 ± 0.003^{abc}

E2	0.24 ± 0.01^{cdefg}	0.21 ± 0.01^{bcdef}	0.14 ± 0.01^{abcd}	0.02 ± 0.01^{d}	0.01 ± 0.01^{b}
E3	0.10 ± 0.01^{ab}	0.42 ± 0.02^{i}	0.24 ± 0.03^{cdefg}	0.03 ± 0.01^{e}	0.01 ± 0.01^{c}
E4	0.28 ± 0.03^{efgh}	0.24 ± 0.03^{cdefg}	0.13 ± 0.03^{abc}	0.02 ± 0.01^{d}	0.01 ± 0.01^{bc}
E5	0.33 ± 0.05^{fgh}	0.18 ± 0.01^{bcde}	0.08 ± 0.02^{ab}	0.01 ± 0.01^{c}	0.00 ± 0.01^{a}
E6	0.33 ± 0.05^{fgh}	0.12 ± 0.01^{abc}	0.07 ± 0.04^{ab}	0.00 ± 0.01^{a}	0.00 ± 0.01^{a}
E7	0.20 ± 0.09^{bcdef}	0.24 ± 0.03^{cdefg}	0.06 ± 0.01^{a}	0.02 ± 0.01^{d}	0.00 ± 0.01^{a}
E8	0.20 ± 0.09^{bcdef}	0.19 ± 0.02^{bcde}	0.09 ± 0.01^{ab}	0.01 ± 0.01^{c}	0.00 ± 0.01^{a}
E9	0.10 ± 0.01^{ab}	0.23 ± 0.02^{cdefg}	0.15 ± 0.02^{bcd}	0.02 ± 0.01^{d}	0.01 ± 0.01^{bc}
E10	0.20 ± 0.05^{bcdef}	0.21 ± 0.002^{bcdefg}	0.18 ± 0.01^{bcde}	0.02 ± 0.01^{d}	0.01 ± 0.01^{c}
E11	0.26 ± 0.01^{defg}	0.34 ± 0.04^{gh}	0.28 ± 0.02^{efgh}	0.03 ± 0.01^{e}	0.02 ± 0.01^{d}
E12	0.26 ± 0.01^{defg}	0.35 ± 0.07^{hi}	0.17 ± 0.01^{bcde}	0.03 ± 0.01^{de}	0.01 ± 0.01^{b}

Each value is presented in the form: mean ± standard deviation (SD).
Different lowercase letters indicate statistically significant differences between values ($p < 0.05$). For content values, letters were determined from two-way ANOVA followed by Tukey HSD post-hoc comparisons. For productivity values, letters were determined using pairwise z-tests based on propagated standard deviations.

Table S21: Violaxanthin content (mg g_{dw}^{-1}) at days 0, 7 and 14, and violaxanthin average productivity, P_{Avg} (mg L^{-1} d^{-1}), at the end of days 7 and 14.

	Violaxanthin content (% w/w)			Violaxanthin P_{Avg} (mg L^{-1} d^{-1})	
	t_0	t_7	t_{14}	$t_0 \rightarrow t_7$	$t_0 \rightarrow t_{14}$
E1	0.114 ± 0.001^{bcde}	0.080 ± 0.010^{abc}	0.040 ± 0.020^{a}	0.006 ± 0.002^{bcde}	0.001 ± 0.001^{ab}
E2	0.114 ± 0.001^{bcde}	0.089 ± 0.004^{bcd}	0.129 ± 0.007^{bcdefg}	0.007 ± 0.0004^{de}	0.007 ± 0.001^{de}
E3	0.083 ± 0.007^{abc}	0.321 ± 0.001^{j}	0.260 ± 0.020^{i}	0.023 ± 0.001^{h}	0.011 ± 0.001^{f}
E4	0.110 ± 0.020^{bcde}	0.066 ± 0.008^{ab}	0.099 ± 0.006^{bcd}	0.005 ± 0.001^{bcd}	0.006 ± 0.001^{cde}
E5	0.160 ± 0.020^{defgh}	0.054 ± 0.001^{a}	0.056 ± 0.001^{a}	0.002 ± 0.001^{a}	0.002 ± 0.001^{a}
E6	0.160 ± 0.020^{defgh}	0.084 ± 0.006^{abc}	0.110 ± 0.02^{bcd}	0.003 ± 0.001^{b}	0.004 ± 0.001^{bc}
E7	0.130 ± 0.060^{bcdef}	0.220 ± 0.04^{hi}	0.125 ± 0.004^{bcde}	0.022 ± 0.004^{gh}	0.008 ± 0.001^{de}
E8	0.130 ± 0.060^{bcdef}	0.206 ± 0.007^{fgh}	0.172 ± 0.005^{efgh}	0.017 ± 0.002^{g}	0.008 ± 0.001^{de}
E9	0.083 ± 0.007^{abc}	0.160 ± 0.020^{cdefg}	0.102 ± 0.006^{bcd}	0.013 ± 0.002^{fg}	0.005 ± 0.001^{c}
E10	0.130 ± 0.030^{bcdef}	0.200 ± 0.020^{ghi}	0.250 ± 0.008^{i}	0.019 ± 0.003^{gh}	0.0132 ± 0.001^{fg}
E11	0.170 ± 0.004^{defgh}	0.130 ± 0.010^{bcdefg}	0.108 ± 0.009^{bcd}	0.011 ± 0.002^{ef}	0.0054 ± 0.001^{bcd}
E12	0.170 ± 0.004^{defgh}	0.110 ± 0.030^{bcd}	0.107 ± 0.008^{bcd}	0.006 ± 0.004^{abcdef}	0.0036 ± 0.001^{b}

Different lowercase letters indicate statistically significant differences ($p < 0.05$; see Table S20).

Table S22: Lutein content (mg g_{dw}^{-1}) at days 0, 7 and 14, and lutein average productivity, P_{Avg} (mg L^{-1} d^{-1}), at the end of days 7 and 14.

	Lutein content (% w/w)			Lutein P_{Avg} (mg L^{-1} d^{-1})	
	t_0	t_7	t_{14}	$t_0 \rightarrow t_7$	$t_0 \rightarrow t_{14}$
E1	1.44 ± 0.01^{defg}	1.00 ± 0.30^{bcdef}	0.60 ± 0.30^{ab}	0.07 ± 0.04^{abcd}	0.02 ± 0.02^{a}
E2	1.442 ± 0.01^{defg}	0.90 ± 0.10^{bcde}	0.80 ± 0.01^{abcd}	0.06 ± 0.01^{b}	0.03 ± 0.01^{a}
E3	0.49 ± 0.07^{a}	2.35 ± 0.08^{h}	1.33 ± 0.10^{cdefg}	0.17 ± 0.01^{e}	0.06 ± 0.01^{b}

E4	1.40 ± 0.30^{defg}	1.00 ± 0.02^{bcdef}	0.98 ± 0.05^{bcdef}	0.08 ± 0.01^{bc}	0.06 ± 0.01^{b}
E5	1.40 ± 0.20^{defg}	1.01 ± 0.06^{bcdef}	0.72 ± 0.07^{abc}	0.08 ± 0.01^{bc}	0.03 ± 0.01^{a}
E6	1.40 ± 0.20^{defg}	0.73 ± 0.05^{abc}	0.80 ± 0.30^{abcd}	0.03 ± 0.01^{a}	0.03 ± 0.02^{a}
E7	1.00 ± 0.30^{bcdef}	1.09 ± 0.01^{bcdef}	0.66 ± 0.01^{abc}	0.10 ± 0.01^{cd}	0.04 ± 0.01^{a}
E8	1.00 ± 0.30^{bcdef}	1.00 ± 0.20^{bcdef}	0.79 ± 0.06^{abcd}	0.07 ± 0.02^{bc}	0.03 ± 0.01^{a}
E9	0.49 ± 0.07^{a}	1.29 ± 0.08^{cdef}	0.70 ± 0.04^{abc}	0.12 ± 0.01^{cd}	0.04 ± 0.01^{a}
E10	1.02 ± 0.03^{bcdef}	1.00 ± 0.10^{bcdef}	1.40 ± 0.10^{defg}	0.08 ± 0.01^{bcd}	0.07 ± 0.01^{b}
E11	1.22 ± 0.01^{bcdef}	1.60 ± 0.20^{fg}	1.60 ± 0.10^{efg}	0.16 ± 0.02^{de}	0.10 ± 0.01^{cd}
E12	1.22 ± 0.01^{bcdef}	1.70 ± 0.40^{g}	1.01 ± 0.05^{bcdef}	0.14 ± 0.04^{bcde}	0.04 ± 0.01^{a}

Different lowercase letters indicate statistically significant differences ($p < 0.05$; see Table S20).

Table S23: Zeaxanthin content (mg g_{dw}^{-1}) at days 0, 7 and 14, and zeaxanthin average productivity, P_{Avg} (mg L^{-1} d^{-1}), at the end of days 7 and 14.

	Zeaxanthin content (% w/w)			Zeaxanthin P_{Avg} (mg L^{-1} d^{-1})	
	t_0	t_7	t_{14}	$t_0 \rightarrow t_7$	$t_0 \rightarrow t_{14}$
E1	0.10 ± 0.01^{ab}	0.16 ± 0.03^{bcd}	0.14 ± 0.07^{bcd}	0.02 ± 0.01^{cdef}	0.01 ± 0.01^{abcd}
E2	0.10 ± 0.01^{ab}	0.16 ± 0.01^{bcd}	0.16 ± 0.01^{bcd}	0.02 ± 0.01^{d}	0.01 ± 0.01^{b}
E3	0.07 ± 0.01^{a}	0.31 ± 0.02^{ef}	0.18 ± 0.01^{bcde}	0.02 ± 0.01^{fg}	0.01 ± 0.01^{b}
E4	0.12 ± 0.01^{abc}	0.35 ± 0.06^{f}	0.29 ± 0.04^{ef}	0.04 ± 0.01^{gh}	0.02 ± 0.01^{defg}
E5	0.06 ± 0.01^{a}	0.24 ± 0.02^{cde}	0.28 ± 0.01^{def}	0.02 ± 0.01^{fg}	0.02 ± 0.01^{e}
E6	0.06 ± 0.01^{a}	0.14 ± 0.01^{bcd}	0.17 ± 0.06^{bcd}	0.01 ± 0.01^{c}	0.01 ± 0.01^{abc}
E7	0.12 ± 0.09^{abc}	0.12 ± 0.02^{abc}	0.12 ± 0.01^{abc}	0.01 ± 0.01^{bcd}	0.01 ± 0.01^{b}
E8	0.12 ± 0.09^{abc}	0.06 ± 0.03^{a}	0.10 ± 0.01^{ab}	0.00 ± 0.01^{ab}	0.01 ± 0.01^{a}
E9	0.07 ± 0.01^{a}	0.32 ± 0.01^{ef}	0.34 ± 0.02^{f}	0.03 ± 0.01^{h}	0.02 ± 0.01^{ef}
E10	0.16 ± 0.09^{bcd}	n.d.^{abc}	0.26 ± 0.02^{de}	n.d.	0.01 ± 0.01^{cd}
E11	0.07 ± 0.01^{a}	0.18 ± 0.02^{bcd}	0.21 ± 0.02^{bcde}	0.02 ± 0.01^{defg}	0.01 ± 0.01^{cd}
E12	0.07 ± 0.01^{a}	0.21 ± 0.04^{bcde}	0.15 ± 0.01^{bcd}	0.02 ± 0.01^{defg}	0.01 ± 0.01^{b}

Different lowercase letters indicate statistically significant differences ($p < 0.05$; see Table S20).

Table S24: β-carotene content (mg g_{dw}^{-1}) at days 0, 7 and 14, and β-carotene average productivity, P_{Avg} (mg L^{-1} d^{-1}), at the end of days 7 and 14.

	β-carotene content (mg g_{dw}^{-1})			β-carotene P_{Avg} (mg L^{-1} d^{-1})	
	t_0	t_7	t_{14}	$t_0 \rightarrow t_7$	$t_0 \rightarrow t_{14}$
E1	0.133 ± 0.001^{cdefg}	0.090 ± 0.020^{bcde}	0.050 ± 0.030^{ab}	0.007 ± 0.002^{cdefg}	0.002 ± 0.002^{abc}
E2	0.133 ± 0.001^{cdefg}	0.085 ± 0.002^{bcd}	0.075 ± 0.003^{abcd}	0.006 ± 0.001^{d}	0.003 ± 0.001^{ac}
E3	0.081 ± 0.006^{abcd}	0.270 ± 0.010^{i}	0.190 ± 0.030^{h}	0.018 ± 0.001^{i}	0.008 ± 0.001^{defg}
E4	0.130 ± 0.020^{cdefg}	0.170 ± 0.020^{fgh}	0.105 ± 0.009^{bcdef}	0.017 ± 0.003^{ghi}	0.007 ± 0.001^{de}
E5	0.150 ± 0.020^{efg}	0.130 ± 0.005^{cdefg}	0.086 ± 0.008^{bcd}	0.010 ± 0.001^{efgh}	0.004 ± 0.001^{c}
E6	0.150 ± 0.020^{efg}	0.095 ± 0.008^{bcde}	0.040 ± 0.020^{a}	0.005 ± 0.001^{cd}	0.000 ± 0.001^{ab}

E7	0.130 ± 0.050^{cdefg}	0.130 ± 0.010^{cdefg}	0.066 ± 0.003^{abc}	0.011 ± 0.002^{efgh}	0.003 ± 0.001^{abc}
E8	0.130 ± 0.050^{cdefg}	0.112 ± 0.009^{bcdef}	0.090 ± 0.002^{bcde}	0.008 ± 0.002^{def}	0.003 ± 0.001^{abc}
E9	0.081 ± 0.006^{abcd}	0.180 ± 0.020^{gh}	0.130 ± 0.010^{cdefg}	0.016 ± 0.002^{hi}	0.007 ± 0.001^{de}
E10	0.130 ± 0.020^{cdefg}	0.130 ± 0.005^{cdefg}	0.190 ± 0.010^{h}	0.011 ± 0.001^{fgh}	0.010 ± 0.001^{efg}
E11	0.127 ± 0.001^{cdefg}	0.140 ± 0.010^{defg}	0.138 ± 0.002^{defg}	0.014 ± 0.002^{gh}	0.008 ± 0.001^{ef}
E12	0.127 ± 0.001^{cdefg}	0.110 ± 0.030^{bcdef}	0.067 ± 0.010^{abc}	$0.007 \pm 0.003^{bcdefgh}$	0.002 ± 0.001^{ab}

Different lowercase letters indicate statistically significant differences ($p < 0.05$; see Table S20).

Table S25: ALA content (mg g_{dw}^{-1}), at days 0, 4, 7 and 14, and ALA average productivity, P_{Avg} (mg L^{-1} d^{-1}), at the end of days 4, 7 and 14.

	ALA content (mg g_{dw}^{-1})				ALA P_{Avg} (mg L^{-1} d^{-1})		
	t_0	t_4	t_7	t_{14}	$t_0 \rightarrow t_4$	$t_0 \rightarrow t_7$	$t_0 \rightarrow t_{14}$
E1	3.56 ± 0.13^{c}	3.90 ± 0.30^{bcd}	2.50 ± 0.40^{b}	2.70 ± 0.07^{fg}	0.01 ± 0.02^{d}	0.19 ± 0.05^{ab}	0.14 ± 0.01^{e}
E2	3.56 ± 0.13^{c}	3.16 ± 0.04^{bc}	2.67 ± 0.08^{b}	2.30 ± 0.04^{def}	-0.02 ± 0.01^{c}	0.20 ± 0.01^{b}	0.10 ± 0.01^{d}
E3	2.30 ± 0.30^{a}	3.00 ± 0.50^{abc}	3.00 ± 0.40^{bc}	2.18 ± 0.16^{def}	0.05 ± 0.04^{cd}	0.15 ± 0.03^{a}	0.07 ± 0.01^{c}
E4	4.51 ± 0.07^{d}	4.49 ± 0.04^{d}	3.43 ± 0.02^{cd}	2.04 ± 0.2^{cde}	-0.00 ± 0.01^{d}	0.29 ± 0.02^{c}	0.10 ± 0.02^{d}
E5	5.11 ± 0.10^{e}	3.50 ± 0.30^{bc}	3.79 ± 0.19^{cd}	1.83 ± 0.04^{cd}	-0.08 ± 0.01^{b}	0.28 ± 0.02^{c}	0.06 ± 0.01^{c}
E6	5.11 ± 0.10^{e}	3.10 ± 0.30^{bc}	2.80 ± 0.18^{b}	1.53 ± 0.04^{bc}	-0.10 ± 0.02^{ab}	0.12 ± 0.02^{a}	0.02 ± 0.01^{b}
E7	5.57 ± 0.14^{e}	2.96 ± 0.05^{abc}	3.12 ± 0.11^{bc}	2.24 ± 0.08^{def}	-0.11 ± 0.01^{a}	0.21 ± 0.02^{b}	0.10 ± 0.01^{d}
E8	5.57 ± 0.14^{e}	3.98 ± 0.03^{cd}	4.43 ± 0.12^{e}	0.96 ± 0.03^{a}	-0.07 ± 0.01^{b}	0.29 ± 0.01^{c}	-0.02 ± 0.01^{a}
E9	2.30 ± 0.30^{a}	2.70 ± 0.20^{ab}	1.80 ± 0.30^{a}	1.40 ± 0.04^{b}	0.02 ± 0.02^{cd}	0.10 ± 0.03^{a}	0.05 ± 0.01^{c}
E10	3.62 ± 0.17^{c}	2.23 ± 0.04^{a}	2.94 ± 0.08^{bc}	1.33 ± 0.07^{b}	-0.07 ± 0.01^{b}	0.23 ± 0.01^{b}	0.03 ± 0.01^{b}
E11	2.90 ± 0.40^{b}	3.10 ± 0.30^{bc}	3.50 ± 0.30^{cd}	2.44 ± 0.18^{ef}	0.01 ± 0.03^{cd}	0.34 ± 0.04^{c}	0.14 ± 0.02^{de}
E12	2.90 ± 0.40^{b}	3.50 ± 0.70^{bc}	4.20 ± 0.30^{de}	2.90 ± 0.30^{g}	0.03 ± 0.05^{cd}	0.35 ± 0.04^{c}	0.13 ± 0.02^{de}

Each value is presented in the form: mean ± standard deviation (SD).
Within the same column, different lowercase letters indicate statistically significant differences between values ($p < 0.05$).
Amino acid data were only subject to comparisons across the set of 12 conditions for each individual time point, for better readability of the results (since more data was available).
For content values, letters were determined from one-way ANOVA followed by Tukey-Kramer post-hoc comparisons.
For productivity values, letters were determined using pairwise z-tests based on propagated standard deviations.

Table S26: ARG content (mg g_{dw}^{-1}), at days 0, 4, 7 and 14, and ARG average productivity, P_{Avg} (mg L^{-1} d^{-1}), at the end of days 4, 7 and 14.

	ARG content (mg g_{dw}^{-1})				ARG P_{Avg} (mg L^{-1} d^{-1})		
	t_0	t_4	t_7	t_{14}	$t_0 \rightarrow t_4$	$t_0 \rightarrow t_7$	$t_0 \rightarrow t_{14}$
E1	12.0 ± 0.7^{b}	7.2 ± 0.6^{a}	19.1 ± 0.1^{c}	15.0 ± 0.2^{c}	-0.2 ± 0.04^{b}	1.9 ± 0.1^{e}	0.9 ± 0.1^{e}
E2	12.0 ± 0.7^{b}	9.7 ± 0.2^{b}	19.2 ± 0.3^{c}	10.6 ± 0.3^{b}	-0.1 ± 0.03^{cd}	1.8 ± 0.1^{e}	0.5 ± 0.1^{c}
E3	9.7 ± 1.8^{ab}	9.7 ± 1.7^{b}	9.9 ± 1.5^{b}	13.3 ± 1.6^{bc}	0.0 ± 0.2^{bcde}	0.4 ± 0.1^{bc}	0.5 ± 0.1^{c}
E4	15.0 ± 1.2^{c}	12.9 ± 0.2^{b}	11.0 ± 0.2^{b}	16.2 ± 0.4^{c}	-0.1 ± 0.1^{bcd}	0.9 ± 0.1^{d}	1.1 ± 0.1^{fg}
E5	15.6 ± 0.4^{c}	6.1 ± 0.2^{a}	11.5 ± 0.6^{b}	13.5 ± 0.2^{bc}	-0.5 ± 0.1^{a}	0.9 ± 0.1^{d}	0.8 ± 0.1^{d}
E6	15.6 ± 0.4^{c}	5.9 ± 0.1^{a}	9.0 ± 1.4^{b}	13.4 ± 0.8^{bc}	-0.5 ± 0.1^{a}	0.4 ± 0.1^{bc}	0.6 ± 0.1^{c}
E7	14.1 ± 0.3^{bc}	13.5 ± 0.7^{bc}	7.8 ± 0.5^{ab}	16.8 ± 0.5^{c}	-0.0 ± 0.1^{cd}	0.5 ± 0.1^{c}	1.1 ± 0.1^{f}
E8	14.1 ± 0.3^{bc}	29.9 ± 1.4^{d}	7.5 ± 0.4^{ab}	1.9 ± 0.1^{a}	0.7 ± 0.1^{f}	0.4 ± 0.1^{b}	-0.1 ± 0.1^{a}
E9	9.7 ± 1.8^{ab}	4.8 ± 0.4^{a}	3.7 ± 0.2^{a}	2.7 ± 0.3^{a}	-0.3 ± 0.1^{abc}	0.1 ± 0.1^{a}	0.0 ± 0.1^{b}
E10	11.3 ± 0.5^{ab}	17.7 ± 0.6^{c}	10.7 ± 0.1^{b}	2.8 ± 0.1^{a}	0.3 ± 0.1^{e}	0.9 ± 0.1^{d}	0.0 ± 0.1^{b}
E11	8.1 ± 1.2^{a}	10.6 ± 1.5^{b}	24.0 ± 4.0^{c}	21 ± 3^{d}	0.1 ± 0.1^{de}	2.7 ± 0.5^{ef}	1.5 ± 0.2^{fg}
E12	8.1 ± 1.2^{a}	11.0 ± 2.0^{b}	24.0 ± 2.0^{c}	26 ± 2^{e}	0.2 ± 0.1^{de}	2.3 ± 0.2^{f}	1.4 ± 0.1^{g}

Within the same column, different lowercase letters indicate statistically significant differences ($p < 0.05$; see Table S25).

Table S27: ASP content (mg g_{dw}^{-1}), at days 0, 4, 7 and 14, and ASP average productivity, P_{Avg} (mg L^{-1} d^{-1}), at the end of days 4, 7 and 14.

	ASP content (mg g_{dw}^{-1})				ASP P_{Avg} (mg L^{-1} d^{-1})		
	t_0	t_0	t_7	t_{14}	$t_0 \rightarrow t_4$	$t_0 \rightarrow t_7$	$t_0 \rightarrow t_{14}$
E1	9.7 ± 0.6^{b}	10.8 ± 0.8^{cd}	10.1 ± 0.3^{bcd}	7.5 ± 0.3^{de}	0.05 ± 0.05^{c}	0.90 ± 0.04^{c}	0.38 ± 0.02^{e}
E2	9.7 ± 0.6^{b}	9.0 ± 0.3^{bc}	8.2 ± 0.5^{abc}	6.3 ± 0.3^{bcd}	-0.03 ± 0.03^{c}	0.66 ± 0.05^{b}	0.29 ± 0.02^{d}
E3	8.0 ± 1.0^{a}	10.2 ± 1.4^{bc}	10.2 ± 1.0^{bcd}	7.9 ± 0.9^{e}	0.20 ± 0.10^{c}	0.52 ± 0.09^{ab}	0.25 ± 0.05^{cd}
E4	12.9 ± 0.2^{c}	12.3 ± 0.2^{d}	9.6 ± 0.2^{bcd}	6.3 ± 0.3^{bcd}	-0.03 ± 0.02^{c}	0.80 ± 0.06^{c}	0.33 ± 0.03^{de}
E5	14.6 ± 0.6^{d}	8.4 ± 0.6^{bc}	10.9 ± 0.6^{cd}	5.6 ± 0.2^{bcd}	-0.30 ± 0.04^{a}	0.82 ± 0.07^{c}	0.21 ± 0.02^{c}
E6	14.6 ± 0.6^{d}	9.1 ± 0.4^{bc}	7.5 ± 1.5^{ab}	5.1 ± 0.3^{bc}	-0.26 ± 0.04^{a}	0.30 ± 0.10^{a}	0.11 ± 0.02^{b}
E7	15.1 ± 0.5^{d}	8.2 ± 0.5^{ab}	8.6 ± 0.2^{bc}	6.5 ± 0.2^{cd}	-0.30 ± 0.03^{a}	0.59 ± 0.04^{b}	0.31 ± 0.02^{d}
E8	15.1 ± 0.5^{d}	12.0 ± 0.3^{cd}	12.5 ± 0.5^{de}	3.1 ± 0.2^{a}	-0.14 ± 0.03^{b}	0.82 ± 0.05^{c}	-0.02 ± 0.01^{a}
E9	8.0 ± 1.0^{a}	9.1 ± 0.6^{bc}	6.5 ± 1.2^{a}	5.1 ± 0.5^{bc}	0.07 ± 0.07^{c}	0.40 ± 0.10^{a}	0.19 ± 0.04^{c}
E10	10.2 ± 0.6^{b}	6.4 ± 0.4^{a}	8.5 ± 0.1^{abc}	4.3 ± 0.3^{ab}	-0.19 ± 0.04^{b}	0.67 ± 0.02^{b}	0.12 ± 0.02^{b}
E11	9.9 ± 0.7^{b}	11.0 ± 1.0^{cd}	12.9 ± 1.2^{de}	9.2 ± 0.6^{f}	0.05 ± 0.07^{c}	1.30 ± 0.20^{d}	0.53 ± 0.06^{f}
E12	9.9 ± 0.7^{b}	11.0 ± 1.0^{cd}	13.8 ± 1.1^{e}	11.1 ± 0.8^{g}	0.05 ± 0.08^{c}	1.20 ± 0.10^{d}	0.49 ± 0.05^{f}

Table S28: GLU content (mg g_{dw}^{-1}), at days 0, 4, 7 and 14, and GLU average productivity, P_{Avg} (mg L^{-1} d^{-1}), at the end of days 4, 7 and 14.

	GLU content (mg g_{dw}^{-1})				GLU P_{Avg} (mg L^{-1} d^{-1})		
	t_0	t_0	t_7	t_{14}	$t_0 \rightarrow t_4$	$t_0 \rightarrow t_7$	$t_0 \rightarrow t_{14}$
E1	11.0 ± 0.8^{b}	13.1 ± 0.7^{bc}	7.8 ± 0.3^{ab}	9.5 ± 0.4^{c}	0.1 ± 0.1^{c}	0.6 ± 0.1^{ab}	0.5 ± 0.1^{e}
E2	11.0 ± 0.8^{b}	10.3 ± 0.4^{ab}	9.0 ± 0.5^{bc}	6.7 ± 0.6^{b}	-0.0 ± 0.1^{b}	0.7 ± 0.1^{b}	0.3 ± 0.1^{d}
E3	7.3 ± 0.9^{a}	10.3 ± 1.6^{ab}	9.3 ± 1.2^{bc}	7.8 ± 0.6^{bc}	0.2 ± 0.1^{bc}	0.5 ± 0.1^{a}	0.2 ± 0.1^{c}
E4	14.7 ± 0.19^{c}	14.5 ± 1.3^{c}	11.2 ± 0.2^{bc}	9.5 ± 1.9^{bcd}	-0.0 ± 0.1^{bc}	0.9 ± 0.1^{c}	0.6 ± 0.2^{de}
E5	16.9 ± 1.1^{cd}	9.3 ± 0.5^{ab}	12.5 ± 0.7^{cd}	7.7 ± 0.9^{bc}	-0.4 ± 0.1^{a}	0.9 ± 0.1^{c}	0.3 ± 0.1^{cd}
E6	16.9 ± 1.1^{cd}	10.0 ± 0.5^{ab}	9.2 ± 1.3^{bc}	5.7 ± 0.3^{ab}	-0.3 ± 0.1^{a}	0.4 ± 0.1^{a}	0.1 ± 0.1^{b}
E7	17.6 ± 1.1^{d}	9.7 ± 0.9^{ab}	9.9 ± 1.1^{bc}	7.4 ± 0.2^{bc}	-0.4 ± 0.1^{a}	0.7 ± 0.1^{abc}	0.4 ± 0.1^{d}
E8	17.6 ± 1.1^{d}	15.0 ± 2.0^{c}	14.2 ± 0.7^{de}	3.9 ± 0.2^{a}	-0.1 ± 0.1^{bc}	0.9 ± 0.1^{c}	-0.0 ± 0.1^{a}
E9	7.3 ± 0.9^{a}	9.0 ± 0.8^{ab}	6.0 ± 1.0^{a}	5.0 ± 0.2^{ab}	0.1 ± 0.1^{bc}	0.4 ± 0.1^{a}	0.2 ± 0.1^{c}
E10	12.1 ± 1.4^{b}	7.8 ± 0.3^{a}	10.2 ± 0.5^{bc}	4.5 ± 0.3^{ab}	-0.2 ± 0.1^{a}	0.8 ± 0.1^{bc}	0.1 ± 0.1^{b}
E11	9.6 ± 1.4^{ab}	11.1 ± 1.5^{b}	13.9 ± 1.7^{d}	8.8 ± 1.3^{bc}	0.1 ± 0.1^{bc}	1.4 ± 0.2^{d}	0.5 ± 0.1^{de}
E12	9.6 ± 1.4^{ab}	14.0 ± 2.0^{bc}	16.5 ± 1.7^{e}	11.7 ± 1.3^{d}	0.2 ± 0.1^{bc}	1.4 ± 0.2^{d}	0.5 ± 0.1^{e}

Table S29: GLY content (mg g_{dw}^{-1}), at days 0, 4, 7 and 14, and GLY average productivity, P_{Avg} (mg L^{-1} d^{-1}), at the end of days 4, 7 and 14.

	GLY content (mg g_{dw}^{-1})				GLY P_{Avg} (mg L^{-1} d^{-1})		
	t_0	t_0	t_7	t_{14}	$t_0 \rightarrow t_4$	$t_0 \rightarrow t_7$	$t_0 \rightarrow t_{14}$
E1	5.2 ± 0.3^{bc}	6.1 ± 0.4^{c}	3.8 ± 0.2^{ab}	4.0 ± 0.2^{bc}	0.04 ± 0.03^{b}	0.30 ± 0.03^{ab}	0.20 ± 0.01^{d}
E2	5.2 ± 0.3^{bc}	5.0 ± 0.1^{b}	4.2 ± 0.4^{bc}	3.6 ± 0.3^{b}	-0.01 ± 0.02^{b}	0.33 ± 0.05^{b}	0.17 ± 0.02^{d}
E3	4.0 ± 0.5^{a}	5.5 ± 0.7^{bc}	4.7 ± 0.6^{bcd}	3.4 ± 0.2^{b}	0.10 ± 0.06^{b}	0.23 ± 0.05^{a}	0.10 ± 0.02^{c}
E4	6.5 ± 0.3^{cd}	6.4 ± 0.4^{c}	5.4 ± 0.1^{cd}	3.2 ± 0.3^{b}	-0.01 ± 0.03^{b}	0.47 ± 0.03^{cd}	0.16 ± 0.02^{d}
E5	7.2 ± 0.4^{de}	5.2 ± 0.3^{b}	5.9 ± 0.2^{de}	3.1 ± 0.2^{b}	-0.10 ± 0.03^{a}	0.46 ± 0.03^{c}	0.13 ± 0.02^{c}
E6	7.2 ± 0.4^{de}	4.7 ± 0.6^{ab}	5.2 ± 0.3^{cd}	2.4 ± 0.1^{a}	-0.12 ± 0.04^{a}	0.29 ± 0.03^{a}	0.05 ± 0.01^{b}
E7	7.8 ± 0.2^{e}	4.9 ± 0.4^{ab}	4.9 ± 0.2^{bcd}	3.7 ± 0.6^{b}	-0.13 ± 0.02^{a}	0.36 ± 0.02^{b}	0.19 ± 0.05^{cd}
E8	7.8 ± 0.2^{e}	5.3 ± 0.2^{bc}	7.7 ± 0.4^{f}	2.0 ± 0.1^{a}	-0.12 ± 0.01^{a}	0.54 ± 0.04^{cd}	0.01 ± 0.01^{a}
E9	4.0 ± 0.5^{a}	4.9 ± 0.3^{b}	3.2 ± 0.5^{a}	2.8 ± 0.3^{ab}	0.05 ± 0.04^{b}	0.19 ± 0.05^{a}	0.11 ± 0.02^{c}
E10	5.8 ± 0.3^{bcd}	3.7 ± 0.3^{a}	4.7 ± 0.3^{bcd}	2.4 ± 0.4^{a}	-0.11 ± 0.02^{a}	0.36 ± 0.03^{b}	0.07 ± 0.03^{bc}
E11	4.7 ± 0.5^{ab}	5.4 ± 0.7^{bc}	5.2 ± 0.6^{cd}	4.6 ± 0.8^{c}	0.04 ± 0.05^{b}	0.50 ± 0.08^{bcd}	0.27 ± 0.06^{d}
E12	4.7 ± 0.5^{ab}	5.7 ± 0.7^{bc}	6.54 ± 0.12^{e}	4.5 ± 0.3^{c}	0.06 ± 0.05^{b}	0.55 ± 0.02^{d}	0.19 ± 0.02^{d}

Table S30: HIS content (mg g_{dw}^{-1}), at days 0, 4, 7 and 14, and HIS average productivity, P_{Avg} (mg L^{-1} d^{-1}), at the end of days 4, 7 and 14.

	HIS content (mg g_{dw}^{-1})				HIS P_{Avg} (mg L^{-1} d^{-1})		
	t_0	t_0	t_7	t_{14}	$t_0 \rightarrow t_4$	$t_0 \rightarrow t_7$	$t_0 \rightarrow t_{14}$
E1	2.9 ± 0.1^{a}	2.6 ± 0.4^{ab}	2.3 ± 0.2^{a}	2.6 ± 0.1^{bc}	-0.01 ± 0.02^{abc}	0.19 ± 0.02^{ab}	0.14 ± 0.01^{d}
E2	2.9 ± 0.1^{a}	2.5 ± 0.1^{ab}	2.4 ± 0.1^{a}	2.3 ± 0.1^{abc}	-0.02 ± 0.01^{b}	0.19 ± 0.01^{a}	0.11 ± 0.01^{c}
E3	3.1 ± 0.1^{a}	3.9 ± 0.5^{c}	3.5 ± 0.4^{c}	2.7 ± 0.2^{bc}	0.06 ± 0.04^{c}	0.17 ± 0.03^{ab}	0.08 ± 0.01^{b}
E4	3.3 ± 0.1^{ab}	3.1 ± 0.2^{b}	2.9 ± 0.1^{b}	2.0 ± 0.1^{ab}	-0.01 ± 0.01^{b}	0.26 ± 0.02^{bc}	0.12 ± 0.01^{c}
E5	3.4 ± 0.3^{ab}	2.6 ± 0.1^{ab}	2.9 ± 0.1^{b}	2.1 ± 0.1^{abc}	-0.04 ± 0.02^{ab}	0.23 ± 0.01^{b}	0.10 ± 0.01^{c}
E6	3.4 ± 0.3^{ab}	2.6 ± 0.3^{ab}	3.0 ± 0.2^{b}	2.0 ± 0.1^{a}	-0.04 ± 0.02^{ab}	0.18 ± 0.02^{a}	0.08 ± 0.01^{b}
E7	3.7 ± 0.1^{b}	2.6 ± 0.2^{ab}	2.5 ± 0.1^{a}	2.5 ± 0.1^{bc}	-0.05 ± 0.01^{a}	0.19 ± 0.01^{a}	0.15 ± 0.01^{d}
E8	3.7 ± 0.1^{b}	2.7 ± 0.1^{ab}	3.7 ± 0.1^{cd}	1.9 ± 0.1^{a}	-0.05 ± 0.01^{a}	0.26 ± 0.01^{c}	0.05 ± 0.01^{a}
E9	3.1 ± 0.1^{a}	3.5 ± 0.2^{bc}	2.6 ± 0.1^{ab}	2.3 ± 0.2^{abc}	0.02 ± 0.01^{c}	0.16 ± 0.02^{a}	0.10 ± 0.01^{bc}
E10	3.0 ± 0.1^{a}	2.2 ± 0.1^{a}	2.5 ± 0.1^{a}	1.9 ± 0.2^{a}	-0.04 ± 0.01^{a}	0.19 ± 0.01^{a}	0.07 ± 0.01^{ab}
E11	3.3 ± 0.1^{ab}	3.5 ± 0.5^{bc}	3.6 ± 0.3^{c}	3.1 ± 0.3^{cd}	0.01 ± 0.03^{bc}	0.34 ± 0.03^{d}	0.18 ± 0.03^{d}
E12	3.3 ± 0.1^{ab}	3.6 ± 0.4^{bc}	4.1 ± 0.2^{d}	3.2 ± 0.3^{d}	0.02 ± 0.02^{bc}	0.33 ± 0.02^{d}	0.14 ± 0.02^{cd}

Table S31: ILE content (mg g_{dw}^{-1}), at days 0, 4, 7 and 14, and ILE average productivity, P_{Avg} (mg L^{-1} d^{-1}), at the end of days 4, 7 and 14.

	ILE content (mg g_{dw}^{-1})				ILE P_{Avg} (mg L^{-1} d^{-1})		
	t_0	t_0	t_7	t_{14}	$t_0 \rightarrow t_4$	$t_0 \rightarrow t_7$	$t_0 \rightarrow t_{14}$
E1	6.0 ± 0.6^{b}	5.9 ± 0.5^{b}	4.3 ± 0.4^{b}	4.4 ± 0.15^{de}	0.00 ± 0.04^{b}	0.34 ± 0.04^{ab}	0.22 ± 0.01^{e}
E2	6.0 ± 0.6^{b}	5.1 ± 0.2^{ab}	4.7 ± 0.4^{bc}	3.8 ± 0.2^{bcd}	-0.04 ± 0.03^{ab}	0.37 ± 0.05^{b}	0.17 ± 0.02^{d}
E3	3.9 ± 0.6^{a}	5.0 ± 0.7^{ab}	4.9 ± 0.7^{bc}	3.4 ± 0.4^{bcd}	0.07 ± 0.06^{bc}	0.25 ± 0.06^{ab}	0.10 ± 0.02^{c}
E4	7.3 ± 0.3^{c}	7.3 ± 0.5^{c}	5.9 ± 0.1^{cd}	3.3 ± 0.4^{bcd}	0.00 ± 0.03^{b}	0.51 ± 0.04^{cd}	0.16 ± 0.03^{cd}
E5	7.8 ± 0.5^{c}	5.6 ± 0.4^{b}	6.5 ± 0.2^{d}	3.1 ± 0.5^{bc}	-0.11 ± 0.03^{a}	0.51 ± 0.02^{c}	0.12 ± 0.04^{bcd}
E6	7.8 ± 0.5^{c}	5.0 ± 0.9^{ab}	4.6 ± 0.7^{bc}	2.4 ± 0.0^{ab}	-0.13 ± 0.05^{a}	0.22 ± 0.06^{a}	0.04 ± 0.01^{b}
E7	8.0 ± 0.4^{c}	5.4 ± 0.6^{b}	5.1 ± 0.3^{bc}	3.9 ± 0.3^{cd}	-0.11 ± 0.03^{a}	0.38 ± 0.03^{b}	0.20 ± 0.02^{de}
E8	8.0 ± 0.4^{c}	6.0 ± 0.3^{b}	8.4 ± 0.4^{e}	1.9 ± 0.1^{a}	-0.10 ± 0.03^{a}	0.61 ± 0.04^{d}	0.00 ± 0.01^{a}
E9	3.9 ± 0.6^{a}	5.0 ± 0.5^{ab}	3.2 ± 0.5^{a}	2.6 ± 0.1^{ab}	0.07 ± 0.05^{bc}	0.20 ± 0.06^{a}	0.10 ± 0.01^{c}
E10	5.7 ± 0.4^{b}	4.0 ± 0.4^{a}	5.3 ± 0.3^{bc}	2.3 ± 0.4^{ab}	-0.09 ± 0.03^{ab}	0.44 ± 0.03^{bc}	0.06 ± 0.03^{bc}
E11	4.8 ± 0.8^{ab}	5.2 ± 0.7^{ab}	5.3 ± 0.4^{c}	4.4 ± 0.3^{de}	0.02 ± 0.06^{b}	0.51 ± 0.05^{cd}	0.26 ± 0.03^{e}
E12	4.8 ± 0.8^{ab}	8.3 ± 0.2^{c}	6.2 ± 0.4^{cd}	5.0 ± 0.4^{e}	0.19 ± 0.04^{c}	0.51 ± 0.05^{cd}	0.22 ± 0.03^{de}

Table S32: LEU content (mg g_{dw}^{-1}), at days 0, 4, 7 and 14, and LEU average productivity, P_{Avg} (mg L^{-1} d^{-1}), at the end of days 4, 7 and 14.

	LEU content (mg g_{dw}^{-1})				LEU P_{Avg} (mg L^{-1} d^{-1})		
	t_0	t_0	t_7	t_{14}	$t_0 \rightarrow t_4$	$t_0 \rightarrow t_7$	$t_0 \rightarrow t_{14}$
E1	7.7 ± 0.5^{b}	8.6 ± 0.8^{bc}	5.4 ± 0.3^{ab}	5.6 ± 0.2^{ef}	0.04 ± 0.05^{cd}	0.41 ± 0.03^{b}	0.28 ± 0.01^{e}
E2	7.7 ± 0.5^{b}	7.1 ± 0.4^{b}	5.9 ± 0.6^{bc}	4.7 ± 0.3^{cdef}	-0.03 ± 0.03^{cd}	0.46 ± 0.07^{bc}	0.21 ± 0.02^{d}
E3	5.2 ± 0.8^{a}	6.8 ± 1.0^{ab}	6.7 ± 0.9^{bc}	4.6 ± 0.4^{cde}	0.11 ± 0.09^{cd}	0.34 ± 0.08^{abc}	0.13 ± 0.02^{c}
E4	10.2 ± 0.1^{c}	9.5 ± 0.4^{c}	7.9 ± 0.1^{cd}	4.1 ± 0.5^{bcde}	-0.04 ± 0.03^{c}	0.67 ± 0.05^{d}	0.18 ± 0.04^{cd}
E5	11.3 ± 0.1^{cd}	7.7 ± 0.8^{b}	8.8 ± 0.3^{d}	4.0 ± 0.6^{bcd}	-0.18 ± 0.04^{ab}	0.67 ± 0.03^{d}	0.13 ± 0.04^{cd}
E6	11.3 ± 0.1^{cd}	6.7 ± 1.3^{ab}	6.4 ± 0.3^{bc}	3.1 ± 0.1^{ab}	-0.22 ± 0.06^{ab}	0.29 ± 0.02^{a}	0.03 ± 0.01^{b}
E7	11.8 ± 0.3^{d}	6.6 ± 0.3^{ab}	6.6 ± 0.3^{bc}	4.9 ± 0.3^{def}	-0.23 ± 0.02^{a}	0.45 ± 0.04^{bc}	0.23 ± 0.03^{d}
E8	11.8 ± 0.3^{d}	7.8 ± 0.2^{bc}	10.9 ± 0.3^{e}	2.5 ± 0.1^{a}	-0.19 ± 0.02^{ab}	0.75 ± 0.03^{d}	-0.02 ± 0.01^{a}
E9	5.2 ± 0.8^{a}	6.8 ± 0.6^{ab}	4.3 ± 0.8^{a}	3.4 ± 0.1^{bc}	0.10 ± 0.06^{d}	0.27 ± 0.09^{ab}	0.13 ± 0.01^{c}
E10	8.5 ± 0.3^{b}	5.2 ± 0.4^{a}	6.7 ± 0.2^{bc}	2.9 ± 0.6^{ab}	-0.17 ± 0.03^{b}	0.51 ± 0.03^{c}	0.06 ± 0.04^{bc}
E11	6.6 ± 1.0^{a}	7.2 ± 0.9^{b}	7.6 ± 0.7^{cd}	5.8 ± 0.1^{fg}	0.03 ± 0.07^{cd}	0.74 ± 0.09^{d}	0.33 ± 0.03^{e}
E12	6.6 ± 1.0^{a}	7.3 ± 0.8^{b}	8.6 ± 0.3^{d}	6.4 ± 0.4^{g}	0.04 ± 0.07^{cd}	0.70 ± 0.04^{d}	0.27 ± 0.03^{de}

Table S33: LYS content (mg g_{dw}^{-1}), at days 0, 4, 7 and 14, and LYS average productivity, P_{Avg} (mg L^{-1} d^{-1}), at the end of days 4, 7 and 14.

	LYS content (mg g_{dw}^{-1})				LYS P_{Avg} (mg L^{-1} d^{-1})		
	t_0	t_0	t_7	t_{14}	$t_0 \rightarrow t_4$	$t_0 \rightarrow t_7$	$t_0 \rightarrow t_{14}$
E1	2.8 ± 0.2^{a}	3.8 ± 0.4^{bc}	2.4 ± 0.4^{ab}	1.5 ± 0.1^{bc}	0.05 ± 0.02^{bc}	0.19 ± 0.05^{bc}	0.06 ± 0.01^{d}
E2	2.8 ± 0.2^{a}	2.9 ± 0.3^{ab}	2.5 ± 0.4^{ab}	1.2 ± 0.1^{b}	0.00 ± 0.02^{b}	0.20 ± 0.04^{b}	0.05 ± 0.01^{c}
E3	3.0 ± 0.5^{a}	3.6 ± 0.6^{bc}	4.3 ± 0.6^{cd}	3.0 ± 0.4^{d}	0.05 ± 0.05^{bc}	0.23 ± 0.05^{bc}	0.09 ± 0.02^{d}
E4	3.7 ± 0.3^{b}	3.6 ± 0.2^{bc}	3.3 ± 0.1^{bc}	1.1 ± 0.1^{ab}	0.00 ± 0.02^{b}	0.29 ± 0.02^{c}	0.03 ± 0.01^{c}
E5	4.5 ± 0.4^{b}	3.0 ± 0.2^{ab}	3.5 ± 0.3^{bcd}	1.0 ± 0.2^{ab}	-0.08 ± 0.02^{a}	0.27 ± 0.03^{c}	0.01 ± 0.02^{b}
E6	4.5 ± 0.4^{b}	2.4 ± 0.3^{a}	2.1 ± 0.0^{a}	0.7 ± 0.1^{a}	-0.10 ± 0.02^{a}	0.07 ± 0.01^{a}	-0.02 ± 0.01^{ab}
E7	4.8 ± 0.7^{b}	2.4 ± 0.1^{ab}	2.2 ± 0.3^{a}	1.3 ± 0.1^{b}	-0.11 ± 0.03^{a}	0.13 ± 0.04^{ab}	0.04 ± 0.01^{c}
E8	4.8 ± 0.7^{b}	3.3 ± 0.5^{b}	3.4 ± 0.6^{bcd}	0.5 ± 0.1^{a}	-0.07 ± 0.04^{ab}	0.21 ± 0.06^{bc}	-0.03 ± 0.01^{a}
E9	3.0 ± 0.5^{a}	3.7 ± 0.4^{bc}	2.6 ± 0.5^{abc}	1.9 ± 0.1^{c}	0.05 ± 0.04^{bc}	0.17 ± 0.05^{b}	0.07 ± 0.01^{d}
E10	3.27 ± 0.2^{a}	2.3 ± 0.3^{a}	2.0 ± 0.1^{a}	0.7 ± 0.2^{a}	-0.05 ± 0.02^{a}	0.14 ± 0.02^{b}	-0.00 ± 0.01^{b}
E11	3.6 ± 0.6^{ab}	4.2 ± 0.6^{c}	4.9 ± 0.3^{de}	3.3 ± 0.3^{d}	0.04 ± 0.05^{bc}	0.49 ± 0.05^{d}	0.19 ± 0.03^{e}
E12	3.6 ± 0.6^{ab}	5.9 ± 0.1^{d}	5.6 ± 0.4^{e}	4.2 ± 0.4^{e}	0.13 ± 0.03^{c}	0.48 ± 0.04^{d}	0.19 ± 0.03^{e}

Table S34: MET content (mg g_{dw}^{-1}), at days 0, 4, 7 and 14, and MET average productivity, P_{Avg} (mg L^{-1} d^{-1}), at the end of days 4, 7 and 14.

	MET content (mg g_{dw}^{-1})				MET P_{Avg} (mg L^{-1} d^{-1})		
	t_0	t_0	t_7	t_{14}	$t_0 \rightarrow t_4$	$t_0 \rightarrow t_7$	$t_0 \rightarrow t_{14}$
E1	2.2 ± 0.2^{b}	2.6 ± 0.3^{bc}	1.4 ± 0.2^{b}	1.6 ± 0.1^{d}	0.02 ± 0.02^{b}	0.10 ± 0.02^{ab}	0.08 ± 0.01^{d}
E2	2.2 ± 0.2^{b}	2.1 ± 0.1^{ab}	1.7 ± 0.1^{bc}	1.3 ± 0.3^{bcd}	-0.00 ± 0.01^{b}	0.13 ± 0.02^{bc}	0.06 ± 0.02^{cd}
E3	1.2 ± 0.2^{a}	1.8 ± 0.3^{ab}	1.6 ± 0.2^{bc}	1.1 ± 0.1^{abc}	0.04 ± 0.02^{b}	0.08 ± 0.02^{a}	0.03 ± 0.01^{bc}
E4	2.8 ± 0.1^{c}	2.9 ± 0.3^{c}	2.3 ± 0.1^{de}	1.2 ± 0.1^{bc}	0.00 ± 0.02^{b}	0.20 ± 0.01^{d}	0.06 ± 0.01^{cd}
E5	3.1 ± 0.4^{c}	2.2 ± 0.1^{b}	2.5 ± 0.1^{e}	1.2 ± 0.2^{bc}	-0.04 ± 0.02^{a}	0.20 ± 0.01^{d}	0.04 ± 0.01^{bc}
E6	3.1 ± 0.4^{c}	1.9 ± 0.2^{ab}	2.0 ± 0.1^{cd}	1.0 ± 0.1^{ab}	-0.06 ± 0.02^{a}	0.11 ± 0.01^{ab}	0.02 ± 0.01^{b}
E7	3.4 ± 0.1^{c}	1.9 ± 0.1^{ab}	2.1 ± 0.1^{cd}	1.5 ± 0.2^{cd}	-0.06 ± 0.01^{a}	0.15 ± 0.01^{bc}	0.07 ± 0.01^{d}
E8	3.4 ± 0.1^{c}	2.3 ± 0.1^{b}	3.3 ± 0.1^{f}	0.8 ± 0.1^{a}	-0.05 ± 0.01^{a}	0.23 ± 0.01^{e}	0.00 ± 0.01^{a}
E9	1.2 ± 0.2^{a}	1.7 ± 0.2^{a}	1.1 ± 0.2^{a}	0.9 ± 0.1^{a}	0.03 ± 0.02^{b}	0.07 ± 0.02^{a}	0.04 ± 0.01^{c}
E10	2.4 ± 0.1^{b}	1.6 ± 0.1^{a}	2.0 ± 0.1^{cd}	0.8 ± 0.1^{a}	-0.04 ± 0.01^{a}	0.16 ± 0.01^{bc}	0.02 ± 0.01^{b}
E11	1.5 ± 0.3^{a}	1.8 ± 0.3^{ab}	1.8 ± 0.2^{cd}	1.3 ± 0.1^{bc}	0.02 ± 0.02^{b}	0.17 ± 0.02^{bcd}	0.07 ± 0.01^{d}
E12	1.5 ± 0.3^{a}	1.9 ± 0.4^{ab}	2.1 ± 0.1^{cd}	1.5 ± 0.1^{bcd}	0.02 ± 0.02^{b}	0.17 ± 0.01^{cd}	0.06 ± 0.01^{d}

Table S35: PHE content (mg g_{dw}^{-1}), at days 0, 4, 7 and 14, and PHE average productivity, P_{Avg} (mg L^{-1} d^{-1}), at the end of days 4, 7 and 14.

	PHE content (mg g_{dw}^{-1})				PHE P_{Avg} (mg L^{-1} d^{-1})		
	t_0	t_0	t_7	t_{14}	$t_0 \rightarrow t_4$	$t_0 \rightarrow t_7$	$t_0 \rightarrow t_{14}$
E1	4.6 ± 0.5^{b}	5.3 ± 0.5^{b}	6.4 ± 0.3^{d}	3.1 ± 0.2^{b}	0.04 ± 0.03^{bc}	0.61 ± 0.04^{d}	0.15 ± 0.01^{e}
E2	4.6 ± 0.5^{b}	4.4 ± 0.4^{b}	3.3 ± 0.6^{ab}	2.8 ± 0.4^{b}	-0.01 ± 0.03^{b}	0.25 ± 0.07^{ab}	0.12 ± 0.03^{de}
E3	1.9 ± 0.1^{a}	5.3 ± 0.2^{b}	3.2 ± 0.5^{ab}	1.8 ± 0.3^{a}	0.23 ± 0.01^{d}	0.19 ± 0.04^{ab}	0.05 ± 0.01^{c}
E4	5.8 ± 0.2^{c}	5.3 ± 0.2^{b}	4.9 ± 0.1^{bc}	2.3 ± 0.2^{b}	-0.03 ± 0.02^{b}	0.44 ± 0.03^{c}	0.10 ± 0.02^{d}
E5	6.5 ± 0.4^{cd}	4.5 ± 0.6^{b}	5.4 ± 0.2^{cd}	2.3 ± 0.4^{b}	-0.10 ± 0.04^{a}	0.43 ± 0.03^{c}	0.08 ± 0.03^{cd}
E6	6.5 ± 0.4^{cd}	3.3 ± 0.5^{a}	4.7 ± 0.6^{bc}	1.3 ± 0.1^{a}	-0.15 ± 0.03^{a}	0.26 ± 0.06^{ab}	-0.01 ± 0.01^{b}
E7	7.0 ± 0.2^{d}	4.1 ± 0.2^{ab}	4.7 ± 0.3^{bc}	3.0 ± 0.5^{b}	-0.13 ± 0.01^{a}	0.35 ± 0.03^{bc}	0.14 ± 0.04^{de}
E8	7.0 ± 0.2^{d}	5.0 ± 0.3^{b}	8.0 ± 1.3^{e}	1.0 ± 0.1^{a}	-0.09 ± 0.02^{a}	0.60 ± 0.10^{cd}	-0.03 ± 0.01^{a}
E9	1.9 ± 0.1^{a}	3.5 ± 0.4^{a}	2.1 ± 0.2^{a}	1.8 ± 0.1^{a}	0.10 ± 0.02^{c}	0.15 ± 0.02^{a}	0.08 ± 0.01^{cd}
E10	5.5 ± 0.8^{c}	3.1 ± 0.5^{a}	4.1 ± 0.4^{bc}	2.0 ± 0.2^{a}	-0.12 ± 0.05^{a}	0.30 ± 0.05^{b}	0.04 ± 0.02^{c}
E11	3.6 ± 0.1^{b}	4.6 ± 0.9^{b}	3.7 ± 0.5^{bc}	2.8 ± 0.6^{b}	0.06 ± 0.05^{bc}	0.35 ± 0.06^{bc}	0.15 ± 0.04^{de}
E12	3.6 ± 0.1^{b}	3.8 ± 0.6^{a}	4.6 ± 0.2^{bc}	2.6 ± 0.2^{b}	0.01 ± 0.03^{b}	0.37 ± 0.02^{bc}	0.10 ± 0.01^{d}

Table S36: PRO content (mg g_{dw}^{-1}), at days 0, 4, 7 and 14, and PRO average productivity, P_{Avg} (mg L^{-1} d^{-1}), at the end of days 4, 7 and 14.

	PRO content (mg g_{dw}^{-1})				PRO P_{Avg} (mg L^{-1} d^{-1})		
	t_0	t_0	t_7	t_{14}	$t_0 \rightarrow t_4$	$t_0 \rightarrow t_7$	$t_0 \rightarrow t_{14}$
E1	5.3 ± 0.1^{a}	6.5 ± 1.3^{b}	3.6 ± 0.3^{a}	4.9 ± 0.4^{cd}	0.06 ± 0.06^{cd}	0.27 ± 0.03^{a}	0.27 ± 0.03^{g}
E2	5.3 ± 0.1^{a}	5.0 ± 0.4^{a}	3.6 ± 0.1^{a}	4.2 ± 0.1^{bc}	-0.01 ± 0.02^{c}	0.27 ± 0.01^{a}	0.21 ± 0.01^{f}
E3	6.1 ± 0.8^{a}	6.1 ± 0.8^{a}	5.9 ± 0.7^{bc}	4.2 ± 0.2^{bc}	0.00 ± 0.08^{bcd}	0.25 ± 0.06^{a}	0.10 ± 0.02^{cd}
E4	6.6 ± 0.1^{a}	7.7 ± 0.5^{b}	5.6 ± 0.2^{b}	2.7 ± 0.1^{ab}	0.06 ± 0.03^{d}	0.49 ± 0.04^{bc}	0.12 ± 0.01^{de}
E5	8.3 ± 1.3^{b}	8.7 ± 0.2^{b}	6.1 ± 0.1^{bc}	2.5 ± 0.5^{a}	0.02 ± 0.07^{cd}	0.45 ± 0.04^{bc}	0.07 ± 0.04^{bcd}
E6	8.3 ± 1.3^{b}	8.4 ± 0.1^{b}	4.7 ± 0.7^{ab}	3.2 ± 0.1^{bc}	0.01 ± 0.06^{bcd}	0.22 ± 0.07^{a}	0.08 ± 0.02^{c}
E7	9.6 ± 0.3^{b}	4.5 ± 0.2^{a}	5.6 ± 0.3^{b}	3.6 ± 0.7^{bc}	-0.22 ± 0.02^{a}	0.39 ± 0.03^{b}	0.16 ± 0.05^{cdef}
E8	9.6 ± 0.3^{b}	6.6 ± 1.4^{b}	8.7 ± 0.6^{d}	1.6 ± 0.2^{a}	-0.14 ± 0.07^{abc}	0.59 ± 0.06^{c}	-0.03 ± 0.01^{a}
E9	6.1 ± 0.8^{a}	5.5 ± 0.7^{a}	4.1 ± 0.6^{a}	4.2 ± 0.3^{bc}	-0.04 ± 0.07^{bcd}	0.21 ± 0.07^{a}	0.16 ± 0.02^{de}
E10	6.7 ± 0.1^{a}	4.4 ± 0.4^{a}	4.3 ± 0.3^{a}	1.7 ± 0.1^{a}	-0.12 ± 0.02^{b}	0.29 ± 0.03^{a}	0.01 ± 0.01^{b}
E11	5.6 ± 0.9^{a}	6.1 ± 0.7^{a}	6.6 ± 0.9^{bc}	5.5 ± 0.6^{d}	0.02 ± 0.06^{cd}	0.60 ± 0.10^{c}	0.33 ± 0.05^{g}
E12	5.6 ± 0.9^{a}	6.4 ± 0.8^{b}	7.0 ± 0.4^{c}	5.0 ± 0.6^{cd}	0.04 ± 0.07^{cd}	0.57 ± 0.06^{c}	0.21 ± 0.04^{efg}

Table S37: SER content (mg g_{dw}^{-1}), at days 0, 4, 7 and 14, and SER average productivity, P_{Avg} (mg L^{-1} d^{-1}), at the end of days 4, 7 and 14.

	SER content (mg g_{dw}^{-1})				SER P_{Avg} (mg L^{-1} d^{-1})		
	t_0	t_0	t_7	t_{14}	$t_0 \rightarrow t_4$	$t_0 \rightarrow t_7$	$t_0 \rightarrow t_{14}$
E1	5.0 ± 0.2^{b}	4.7 ± 0.4^{bc}	3.0 ± 0.1^{a}	3.8 ± 0.12^{de}	-0.01 ± 0.02^{d}	0.21 ± 0.01^{a}	0.20 ± 0.01^{e}
E2	5.0 ± 0.2^{b}	3.8 ± 0.1^{ab}	3.5 ± 0.4^{ab}	3.1 ± 0.5^{bcd}	-0.05 ± 0.01^{cd}	0.26 ± 0.05^{abc}	0.14 ± 0.03^{cde}
E3	3.3 ± 0.4^{a}	4.8 ± 0.6^{bc}	4.8 ± 0.1^{bc}	3.3 ± 0.2^{cde}	0.10 ± 0.05^{e}	0.27 ± 0.02^{b}	0.10 ± 0.01^{cd}
E4	6.3 ± 0.1^{c}	5.2 ± 0.1^{c}	4.1 ± 0.1^{bc}	2.9 ± 0.3^{bcd}	-0.06 ± 0.01^{c}	0.32 ± 0.03^{bc}	0.14 ± 0.02^{cd}
E5	7.1 ± 0.4^{cd}	3.8 ± 0.5^{ab}	4.8 ± 0.2^{bc}	2.7 ± 0.3^{bcd}	-0.16 ± 0.03^{ab}	0.34 ± 0.02^{c}	0.10 ± 0.02^{cd}
E6	7.1 ± 0.4^{cd}	4.3 ± 0.6^{bc}	4.6 ± 0.1^{bc}	2.1 ± 0.1^{abc}	-0.13 ± 0.03^{ab}	0.24 ± 0.01^{ab}	0.03 ± 0.006^{b}
E7	7.7 ± 0.2^{d}	3.7 ± 0.2^{ab}	4.5 ± 0.6^{bc}	3.1 ± 0.2^{bcd}	-0.17 ± 0.01^{a}	0.31 ± 0.06^{abc}	0.15 ± 0.02^{d}
E8	7.7 ± 0.2^{d}	4.6 ± 0.2^{bc}	6.4 ± 0.1^{d}	1.5 ± 0.0^{a}	-0.14 ± 0.01^{ab}	0.42 ± 0.02^{d}	-0.01 ± 0.01^{a}
E9	3.3 ± 0.4^{a}	4.0 ± 0.4^{b}	2.8 ± 0.4^{a}	2.3 ± 0.2^{bcd}	0.04 ± 0.03^{de}	0.18 ± 0.04^{ab}	0.09 ± 0.01^{c}
E10	5.3 ± 0.1^{b}	2.8 ± 0.4^{a}	4.0 ± 0.5^{b}	1.9 ± 0.4^{ab}	-0.13 ± 0.02^{b}	0.30 ± 0.06^{abc}	0.04 ± 0.02^{b}
E11	4.0 ± 0.7^{a}	4.5 ± 0.6^{bc}	4.9 ± 0.4^{c}	3.6 ± 0.5^{cde}	0.02 ± 0.05^{cde}	0.48 ± 0.06^{d}	0.21 ± 0.04^{de}
E12	4.0 ± 0.7^{a}	5.0 ± 0.8^{c}	5.8 ± 0.3^{d}	4.0 ± 0.4^{e}	0.06 ± 0.06^{de}	0.49 ± 0.04^{d}	0.17 ± 0.03^{de}

Table S38: THR content (mg g_{dw}^{-1}), at days 0, 4, 7 and 14, and THR average productivity, P_{Avg} (mg L^{-1} d^{-1}), at the end of days 4, 7 and 14.

	THR content (mg g_{dw}^{-1})				THR P_{Avg} (mg L^{-1} d^{-1})		
	t_0	t_0	t_7	t_{14}	$t_0 \rightarrow t_4$	$t_0 \rightarrow t_7$	$t_0 \rightarrow t_{14}$
E1	4.2 ± 0.2^{b}	4.3 ± 0.4^{b}	2.7 ± 0.1^{a}	2.9 ± 0.1^{b}	0.00 ± 0.02^{cd}	0.20 ± 0.01^{a}	0.14 ± 0.01^{d}
E2	4.2 ± 0.2^{b}	3.6 ± 0.2^{ab}	3.0 ± 0.3^{ab}	2.5 ± 0.2^{b}	-0.03 ± 0.01^{c}	0.22 ± 0.03^{ab}	0.11 ± 0.01^{c}
E3	2.9 ± 0.4^{a}	4.6 ± 0.8^{b}	3.6 ± 0.7^{bc}	2.7 ± 0.3^{b}	0.11 ± 0.06^{d}	0.18 ± 0.06^{ab}	0.08 ± 0.02^{bc}
E4	5.5 ± 0.1^{c}	4.8 ± 0.3^{b}	3.7 ± 0.1^{bc}	2.2 ± 0.3^{ab}	-0.04 ± 0.02^{c}	0.29 ± 0.02^{b}	0.09 ± 0.02^{bcd}
E5	6.1 ± 0.1^{cd}	3.7 ± 0.3^{b}	4.1 ± 0.2^{bc}	2.1 ± 0.2^{a}	-0.11 ± 0.02^{ab}	0.29 ± 0.02^{b}	0.07 ± 0.02^{bc}
E6	6.1 ± 0.1^{cd}	3.7 ± 0.8^{b}	3.5 ± 0.3^{bc}	1.7 ± 0.1^{a}	-0.11 ± 0.04^{ab}	0.16 ± 0.03^{a}	0.02 ± 0.01^{a}
E7	6.4 ± 0.1^{d}	3.4 ± 0.2^{a}	3.3 ± 0.1^{bc}	2.6 ± 0.3^{b}	-0.13 ± 0.01^{a}	0.21 ± 0.01^{a}	0.12 ± 0.02^{cd}
E8	6.4 ± 0.1^{d}	3.8 ± 0.1^{b}	5.0 ± 0.1^{d}	1.5 ± 0.2^{a}	-0.12 ± 0.01^{ab}	0.32 ± 0.01^{bc}	0.00 ± 0.01^{a}
E9	2.9 ± 0.4^{a}	3.5 ± 0.5^{a}	2.4 ± 0.3^{a}	2.0 ± 0.2^{a}	0.03 ± 0.04^{cd}	0.15 ± 0.04^{a}	0.08 ± 0.01^{bc}
E10	4.5 ± 0.1^{b}	2.5 ± 0.3^{a}	3.1 ± 0.2^{ab}	2.0 ± 0.1^{a}	-0.10 ± 0.01^{b}	0.22 ± 0.02^{a}	0.06 ± 0.01^{b}
E11	3.4 ± 0.5^{a}	3.8 ± 0.7^{b}	4.0 ± 0.5^{bc}	2.9 ± 0.4^{b}	0.03 ± 0.05^{cd}	0.38 ± 0.07^{bc}	0.17 ± 0.03^{cd}
E12	3.4 ± 0.5^{a}	4.2 ± 0.8^{b}	4.6 ± 0.3^{cd}	3.6 ± 0.6^{c}	0.05 ± 0.05^{cd}	0.38 ± 0.03^{c}	0.16 ± 0.04^{cd}

Table S39: TYR content (mg g_{dw}^{-1}), at days 0, 4, 7 and 14, and TYR average productivity, P_{Avg} (mg L^{-1} d^{-1}), at the end of days 4, 7 and 14.

	TYR content (mg g_{dw}^{-1})				TYR P_{Avg} (mg L^{-1} d^{-1})		
	t_0	t_0	t_7	t_{14}	$t_0 \rightarrow t_4$	$t_0 \rightarrow t_7$	$t_0 \rightarrow t_{14}$
E1	4.8 ± 0.4^{bc}	5.2 ± 0.4^{b}	3.5 ± 0.5^{ab}	3.8 ± 0.1^{de}	0.02 ± 0.03^{bc}	0.27 ± 0.06^{ab}	0.20 ± 0.01^{f}
E2	4.8 ± 0.4^{bc}	4.2 ± 0.1^{a}	3.6 ± 0.1^{b}	3.2 ± 0.1^{bcde}	-0.03 ± 0.02^{b}	0.28 ± 0.01^{b}	0.14 ± 0.01^{e}
E3	3.5 ± 0.5^{a}	5.2 ± 0.8^{b}	4.2 ± 0.5^{bc}	3.1 ± 0.2^{bcd}	0.11 ± 0.07^{c}	0.20 ± 0.05^{ab}	0.09 ± 0.01^{cd}
E4	5.9 ± 0.3^{bcd}	5.6 ± 0.1^{b}	4.7 ± 0.1^{c}	2.8 ± 0.2^{bc}	-0.02 ± 0.02^{bc}	0.40 ± 0.03^{c}	0.14 ± 0.02^{de}
E5	6.3 ± 0.7^{cd}	4.6 ± 0.1^{ab}	4.8 ± 0.1^{c}	2.7 ± 0.1^{bc}	-0.09 ± 0.04^{ab}	0.37 ± 0.02^{c}	0.11 ± 0.01^{cd}
E6	6.3 ± 0.7^{cd}	4.3 ± 0.5^{a}	4.4 ± 0.4^{bc}	2.5 ± 0.1^{bc}	-0.10 ± 0.04^{ab}	0.24 ± 0.04^{ab}	0.06 ± 0.01^{bc}
E7	7.1 ± 0.2^{d}	4.1 ± 0.2^{a}	4.0 ± 0.2^{b}	3.6 ± 0.2^{cde}	-0.13 ± 0.01^{a}	0.28 ± 0.02^{b}	0.19 ± 0.01^{f}
E8	7.1 ± 0.2^{d}	5.0 ± 0.3^{b}	6.4 ± 0.3^{d}	1.7 ± 0.1^{a}	-0.09 ± 0.02^{a}	0.44 ± 0.03^{cd}	0.00 ± 0.01^{a}
E9	3.5 ± 0.5^{a}	4.1 ± 0.4^{a}	2.8 ± 0.4^{a}	2.3 ± 0.1^{ab}	0.04 ± 0.04^{bc}	0.17 ± 0.05^{a}	0.09 ± 0.01^{cd}
E10	5.3 ± 0.3^{bc}	3.4 ± 0.1^{a}	3.8 ± 0.1^{b}	2.1 ± 0.1^{ab}	-0.09 ± 0.02^{a}	0.28 ± 0.02^{b}	0.05 ± 0.01^{b}
E11	4.0 ± 0.6^{ab}	4.8 ± 0.7^{b}	4.7 ± 0.4^{c}	4.5 ± 0.7^{f}	0.04 ± 0.05^{bc}	0.46 ± 0.05^{cd}	0.27 ± 0.06^{f}
E12	4.0 ± 0.6^{ab}	5.0 ± 0.8^{b}	5.8 ± 0.04^{d}	4.2 ± 0.3^{ef}	0.06 ± 0.05^{bc}	0.49 ± 0.02^{d}	0.18 ± 0.02^{f}

Table S40: VAL content (mg g_{dw}^{-1}), at days 0, 4, 7 and 14, and VAL average productivity, P_{Avg} (mg L^{-1} d^{-1}), at the end of days 4, 7 and 14.

	VAL content (mg g_{dw}^{-1})				VAL P_{Avg} (mg L^{-1} d^{-1})		
	t_0	t_0	t_7	t_{14}	$t_0 \rightarrow t_4$	$t_0 \rightarrow t_7$	$t_0 \rightarrow t_{14}$
E1	5.7 ± 0.3^{b}	6.8 ± 0.6^{b}	4.6 ± 0.2^{b}	4.2 ± 0.1^{d}	0.05 ± 0.03^{c}	0.37 ± 0.03^{b}	0.21 ± 0.01^{e}
E2	5.7 ± 0.3^{b}	5.7 ± 0.3^{a}	5.0 ± 0.5^{bc}	3.6 ± 0.3^{bcd}	0.00 ± 0.02^{c}	0.41 ± 0.06^{b}	0.16 ± 0.02^{d}
E3	4.6 ± 0.6^{a}	5.8 ± 0.9^{a}	5.8 ± 0.8^{cd}	4.0 ± 0.5^{cd}	0.08 ± 0.08^{c}	0.30 ± 0.07^{ab}	0.11 ± 0.03^{cd}
E4	7.5 ± 0.1^{c}	7.6 ± 0.5^{b}	6.4 ± 0.1^{cde}	3.1 ± 0.4^{bc}	0.01 ± 0.03^{c}	0.56 ± 0.04^{c}	0.14 ± 0.03^{cde}
E5	8.4 ± 0.1^{cd}	6.1 ± 0.4^{b}	7.1 ± 0.3^{de}	2.9 ± 0.5^{bc}	-0.11 ± 0.02^{ab}	0.56 ± 0.03^{c}	0.10 ± 0.04^{cd}
E6	8.4 ± 0.1^{cd}	4.9 ± 0.9^{a}	4.9 ± 0.2^{bc}	2.3 ± 0.1^{abc}	-0.17 ± 0.04^{ab}	0.23 ± 0.01^{a}	0.02 ± 0.01^{b}
E7	8.7 ± 0.1^{d}	5.3 ± 0.2^{a}	5.2 ± 0.2^{bc}	3.7 ± 0.3^{cd}	-0.15 ± 0.01^{a}	0.37 ± 0.03^{b}	0.18 ± 0.02^{de}
E8	8.7 ± 0.1^{d}	6.4 ± 0.2^{b}	8.6 ± 0.2^{f}	1.8 ± 0.1^{a}	-0.10 ± 0.01^{b}	0.61 ± 0.02^{c}	-0.01 ± 0.01^{a}
E9	4.6 ± 0.6^{a}	5.7 ± 0.5^{a}	3.5 ± 0.7^{a}	2.9 ± 0.2^{bc}	0.07 ± 0.05^{c}	0.20 ± 0.08^{a}	0.11 ± 0.02^{c}
E10	6.1 ± 0.2^{b}	4.4 ± 0.4^{a}	5.4 ± 0.2^{bc}	2.1 ± 0.4^{ab}	-0.09 ± 0.02^{b}	0.42 ± 0.03^{b}	0.04 ± 0.03^{b}
E11	5.6 ± 0.9^{ab}	6.1 ± 0.8^{b}	6.4 ± 0.5^{cde}	5.0 ± 0.2^{e}	0.03 ± 0.07^{c}	0.62 ± 0.07^{c}	0.29 ± 0.03^{f}
E12	5.6 ± 0.9^{ab}	6.6 ± 1.3^{b}	7.5 ± 0.5^{ef}	5.8 ± 0.5^{f}	0.06 ± 0.08^{bc}	0.63 ± 0.06^{c}	0.25 ± 0.03^{ef}
